# Socioeconomic Status and the Gut Microbiome: A TwinsUK Cohort Study

**DOI:** 10.3390/microorganisms7010017

**Published:** 2019-01-11

**Authors:** Ruth C. E. Bowyer, Matthew A. Jackson, Caroline I. Le Roy, Mary Ni Lochlainn, Tim D. Spector, Jennifer B. Dowd, Claire J. Steves

**Affiliations:** 1 The Department of Twin Research, Kings College London, 3-4th Floor South Wing Block D, St Thomas’ Hospital, Westminster Bridge Road, London SE1 7EH, UK; ruth.c.bowyer@kcl.ac.uk (R.C.E.B.); matthew.jackson@kennedy.ox.ac.uk (M.A.J.); caroline.le_roy@kcl.ac.uk (C.I.L.R.); mary.ni_lochlainn@kcl.ac.uk (M.N.L.); tim.spector@kcl.ac.uk (T.D.S.); 2 Kennedy Institute of Rheumatology, University of Oxford, Oxford OX1 3QR, UK; 3 Clinical Age Research Unit, Kings College Hospital Foundation Trust, London SE5 9RS, UK; 4 Department of Global Health & Social Medicine, King’s Building, King’s College London, Strand, London WC2R 2LS, UK; jennifer.dowd@kcl.ac.uk; 5 CUNY Graduate School of Public Health and Health Policy, 55 W 125th Street, New York, NY 10027, USA; 6 Department of Ageing and Health, St Thomas’ Hospital, 9th floor, North Wing, Westminster Bridge Road, London SE1 7EH, UK

**Keywords:** microbiome, microbiota, sociobiome, socioeconomic status, SES

## Abstract

Socioeconomic inequalities in health and mortality are well established, but the biological mechanisms underlying these associations are less understood. In parallel, the gut microbiome is emerging as a potentially important determinant of human health, but little is known about its broader environmental and social determinants. We test the association between gut microbiota composition and individual- and area-level socioeconomic factors in a well-characterized twin cohort. In this study, 1672 healthy volunteers from twin registry TwinsUK had data available for at least one socioeconomic measure, existing fecal 16S rRNA microbiota data, and all considered co-variables. Associations with socioeconomic status (SES) were robust to adjustment for known health correlates of the microbiome; conversely, these health-microbiome associations partially attenuated with adjustment for SES. Twins discordant for IMD (Index of Multiple Deprivation) were shown to significantly differ by measures of compositional dissimilarity, with suggestion the greater the difference in twin pair IMD, the greater the dissimilarity of their microbiota. Future research should explore how SES might influence the composition of the gut microbiota and its potential role as a mediator of differences associated with SES.

## 1. Introduction

There is increasing evidence that the human gut microbiota play an important role in a broad range of physiological functions, including immune system maturation, metabolic and inflammatory processes, and health deficits [1,2,3]. Despite rapid advances, scientific knowledge of the sources of inter-individual variation in the microbiome and how this evolves over the life course is in its infancy. Recent findings suggest that genetic factors explain a limited amount of variation in the microbiome, pointing to “environmental” factors as the primary driver of microbiome composition [4]. While work on specific nutritional and environmental exposures is rapidly accelerating [5,6], we know little about how broader social and environmental conditions influence the structure and function of the microbiome [7].

The social environment may influence the human microbiome across the life course through a variety of pathways [8]. Early life exposures such as mode of delivery (vaginal or caesarean section), initiation and duration of breastfeeding, antibiotic use, interactions with the indoor and outdoor environment, and dietary habits are likely to be highly influenced by social status and relationships [9,10,11]. Recent studies in primates suggest that social relationships impact the composition of the gut microbiota through microbial sharing between individuals [12,13,14,15]. There is also evidence of interactions between social and physical environments. Cohabiting humans have more similar microbial communities compared to those living apart and shifts in older adults’ gut microbiota composition are observed upon moving from community dwelling to a nursing home [16,17]. 

In addition to direct microbial sharing, psychosocial stressors, which are positively associated with higher deprivation, may modulate the microbiome [18,19]. Exposure to social stressors has been shown to alter homeostatic interactions between the intestinal microbiota and the immune system in mice, leading to increased susceptibility to enteric infection and overproduction of inflammatory mediators that induce anxiety-like behavior [20]. Prenatal maternal stress, associated with lower subjective socioeconomic status, and maternal neglect has also been shown to impact the gut microbiota of offspring mice [21,22,23,24,25]. In rhesus monkeys whose mothers were exposed to startle stressors during pregnancy, lactobacilli levels in the gut microbiota were lower during the first six months of life, which in turn disrupted the development of natural resistance to the enteric pathogen *Shigella flexneri* (48). Evidence from germ-free mice also suggests that the microbiome may directly influence social behavior through host-microbiome interactions during early brain development, particularly in the amygdala [26]. Overall, the animal evidence is suggestive of important pathways linking social factors to the microbiome, motivating the need for studies of these dynamics in human populations.

Thus far, research on social factors and the microbiome in humans is limited. A small study of forty-four healthy volunteers in Chicago found that lower area-level socioeconomic status (SES) was associated with reduced alpha-diversity, greater abundance of *Bacteroides*, and lower abundance of *Prevotella* in the colonic microbiota, providing preliminary evidence of associations between SES and the microbiome [27]. To our knowledge, no existing studies have tested the association of individual-level socioeconomic factors and the composition of the gut microbiome. The current study will test the association between both individual- and area-level socioeconomic measures and the composition of the gut microbiome in a well characterized cohort of twins. It will also examine whether any observed associations are explained by known correlates of microbiome composition including diet and existing health deficits. 

## 2. Materials and Methods 

### 2.1. Data

Data come from the TwinsUK study, the UK’s largest research cohort of adult twins (http://www.twinsuk.ac.uk/) [28]. The study was started in 1992 and now incorporates roughly 13,000 male and female twins aged 18–103 who have been extensively studied for a wide range of clinical and behavioral outcomes. All sociodemographic and health variables were matched to the nearest microbiome sample date. Here, 1672 individuals had data available for at least one socioeconomic measure and all included co-variables. The analytical sample size varies depending on the availability of the SES variable for each respondent (ranging from 1672 to 799); missing SES data was due to differences in questionnaires dependent on the year the twin joined the study, as shown in Table 1. 

The European Bioinformatics Institute (EBI) accession number for the 16S sequences reported in this paper is ERP015317. Metadata used in this analysis is provided upon application to our data access committee website http://twinsuk.ac.uk/resources-for-researchers/access-our-data/.

#### Ethics Approval and Consent to Participate

Favorable ethical opinion was granted by the formerly known St. Thomas’ Hospital Research Ethics Committee (REC). Following restructure and merging of REC, subsequent amendments were approved by the NRES Committee London—Westminster (TwinsUK, REC ref: EC04/015, 1 November 2011); use of microbiota samples was granted NRES Committee London—Westminster (The Flora Twin Study, REC ref: 12/LO/0227, 1 November 2011)

### 2.2. Microbiota Sample Processing

This study used a subset of samples from a previous study [29]. Fecal sample collection, bacterial DNA extraction, amplification, and sequencing have previously been described [29]. Briefly, samples were stored by participants in sealed ice packs and either received by the research department during clinical visits or via post. Samples were stored at −80 °C and were subsequently shipped frozen to Cornell University (Ithaca, NY, USA), where DNA was extracted and the V4 region of the 16S rRNA gene was amplified. The Illumina MiSeq platform (Illumina, San Diego, CA, USA) was used to sequence the amplicons via a multiplexed approach. Subsequent to demultiplexing, sample read paired ends were merged using a 200 nt minimum overlap. USEARCH was used for de novo identification of per sample chimeric sequences, which were subsequently removed. A similarity threshold of 97% was used for picking de novo operational taxonomic units (OTUs) using SUMACLUST within QIIME version 1.9.1. The phylogenetic tree required for calculation of weighted-UniFrac distances was also created in QIIME version 1.9.1 using the make_phylogeny command and default parameters. 

### 2.3. Measures

#### 2.3.1. Socioeconomic Status (SES) 

SES was measured using two individual-level and one area-level indicator. Area-level SES was measured using the Index of Multiple Deprivation 2015 (IMD), a composite of seven different domains representing income, employment, education, skills and training, health deprivation and disability, crime, barriers to housing and services, and living environment deprivation, based on the postcode (or UK grid reference mapped to postcode) where a participant lived at the time of sample collection [30]. Scottish and English/Welsh datasets, provided online by the Scottish government and Public Health England were mapped to participants using RStudio [31] or QGis [32]. Quintiles of the IMD were generated, and reverse-coded to match the other two measures (1 = most deprived, 5 = least deprived, *n* = 1672). Education level was self-reported in 2014–2015 by the respondent (*n* = 1426) as the highest academic credential they had received and grouped into four categories: No qualification or NVQ1/SVQ; O-Level, GCSE, NVQ2/SVQ2, or Scottish Intermediate; Scottish Higher, NVQ3, city and guilds, Pitman, A Level, Scottish Advanced Higher, or Higher Vocational training; University degree, Postgraduate degree, NVQ5, or SVQ5. Annual household income was assessed from questionnaire data with seven response categories ranging from <£5000 to >£50,000 (*n* = 799). The nearest time point of available data to the microbiome collection was chosen from self-responses taken over the period 2004–2014. For analysis, income was grouped into four categories of roughly equal number of individuals: 1. <£14,999, 2. £15,000–£24,999, 3. £25,000–£49,999, and 4. >£50,000. 

#### 2.3.2. Covariates

Current health deficit was measured using a single composite measure, the Frailty Index (FI), which comprises the proportion of health deficits from a total of 39 binary domains of physical and mental health. The FI relates deficit accumulation to an individual’s risk of death [33]. The measure has been previously associated with microbiota composition in this cohort [3], and this approach reduces multiple testing of individual health deficits. The FI was root normalized, as is hereafter referred to as “health deficit”. Dietary composition was measured via Food Frequency Questionnaire data and summarized using the Healthy Eating Index 2010 (HEI), which has been previously used within this cohort in diet and microbiota association studies and was shown to best capture diet variance associated with gut microbiota [34]. Smoking has not been previously observed as correlating with the microbiota within this cohort and so was not considered as a covariate [3].

### 2.4. Microbiome analysis

Microbiome composition was analyzed with respect to (1) alpha diversity, (2) beta diversity, and (3) differential abundance of OTUs. All analysis was carried out in RStudio [31].

Three measures of alpha diversity (Chao1, Shannon diversity, and Simpson index) were calculated on the full untrimmed OTU tables using phyloseq [35]. Linear mixed effects models were constructed using the lme4 and ImerTest packages [36,37] with these measures as response variables. Biological covariates included the HEI, health deficit, Body Mass Index (BMI) (kg/m^2^), and age. Hierarchical models were constructed for each SES variable with and without biological covariates. Technical covariates including log10 transformed library size (i.e., total reads per sample), familial relatedness, mode of collection, and sequencing run were included in all models. 

Bray–Curtis and weighted UniFrac distances were calculated on variance stabilized OTU tables (as outlined in Reference [38]) using R packages vegan and phyloseq [35,39]). Non-parametric multivariate analysis of variance tests (NPMANOVA) were performed for each SES variable in crude and adjusted models. Five thousand permutations were run for each NPMANOVA. Tests for homogeneity of dispersion were performed for each SES variable at 999 permutations. 

DeSeq2 v.1.16.1 was used to calculate differential abundance of OTUs between the least deprived and most deprived group of each SES variable [40]. Models were run with and without biological covariates. A Benjamini–Hochberg false discovery rate transformation was applied to the resulting p-values. OTUs were collapsed to family, order, and phylum levels, and hierarchical models run for each SES variable, adjusted for each potential mediator individually (health deficit, age, BMI, diet), then fully adjusted. As a comparative measure, BMI and health deficit were transformed to factor variables using appropriate thresholds [41,42], as shown in Additional File 1.1, and used in models of differential abundance at OTU level in crude models and separately adjusted for each SES variable. 

Analysis of twins discordant for SES was also performed. Twin discordance was calculated in two ways. In a conservative approach, twins were coded as discordant for a particular SES variable where one twin was in the most deprived category and their co-twin was in the top two least-deprived categories. Analysis was repeated using a less-stringent threshold of discordance, where pairs were treated as discordant if their SES grouping differed by more than one category. Differences in the three measures of alpha diversity were assessed via paired Wilcox rank sum tests. Paired tests of OTU abundances were performed using edgeR [43]. To reduce multiple testing burden, only OTUs significant for each SES factor as above were used within the models, again with a Benjamini–Hochberg false discovery rate transformation. 

All twins within each SES variable were used in paired tests to assess if microbiota dissimilarity (captured via Bray–Curtis and weighted UniFrac measures) increased with differences in SES. For this analysis, the beta diversity distance between each pair was used as the response variable in a regression model. The difference of values between each twin pair’s SES, BMI, diet, health deficit, and library size was calculated and used as covariates. 

## 3. Results

Descriptive statistics for the sample are shown in Table 1. Ethnicity data was available for at least 92% of each subset, with all subsets >96% white British. Overall, lower SES was associated with less healthy diet, more health deficits, and higher BMI, as shown in Additional File 2. 

**Figure 1:**
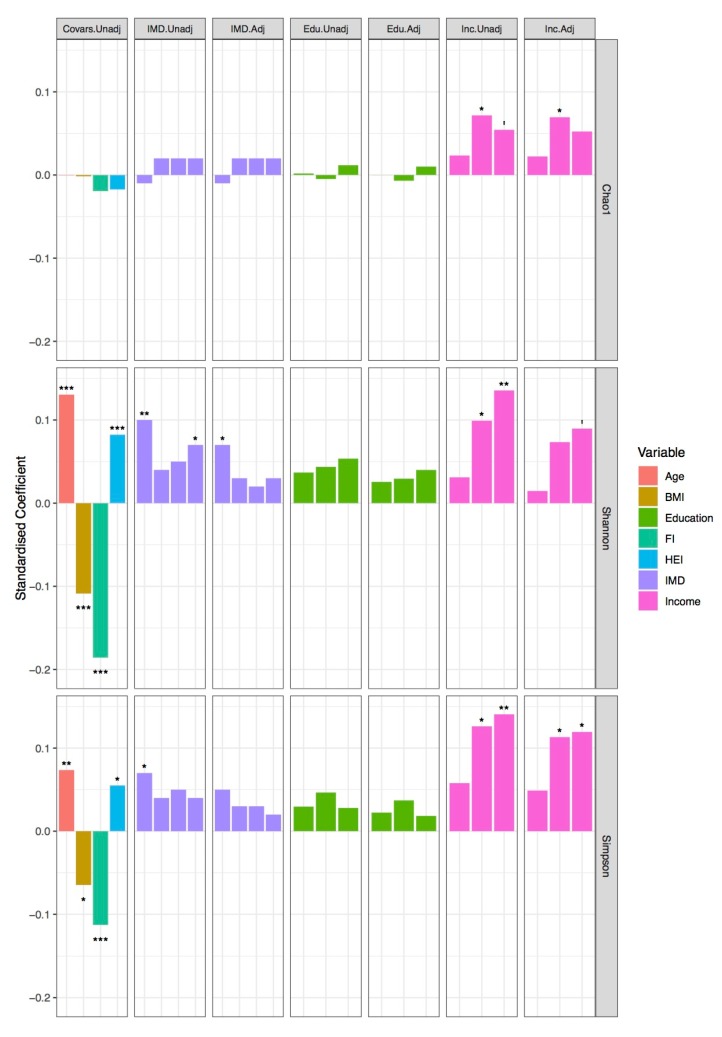
Alpha diversity and socioeconomic status. Bars represent the standardized coefficients extracted from hierarchical linear mixed effects models of alpha diversity (Chao1, Shannon diversity index, and Simpson’s diversity index): i. Covariate model, where model variables were age, Body Mass Index (BMI kg/m^2^), health deficit (FI), and diet (HEI); ii. crude income model, iii. adjusted income model, iv. crude IMD model, and v. IMD-adjusted model. All models were adjusted for technical covariates modelled as random effects. Education models are not included due to non-significance. *p*-values indicated as: ‘ < 0.1, * < 0.05, ** < 0.01, *** < 0.001.

We modelled alpha diversity measures versus SES, as shown in Figure 1, Additional File 1.2, and Additional File 3. Higher levels of income and area-level SES were broadly positively associated with measures of alpha diversity, while a non-significant but positive association with higher levels of education was found. The coefficients for BMI, age, and diet were attenuated in models including income, suggesting that income could explain some of the variance attributed to these factors. For IMD, coefficients were reduced in adjusted models, but the general trend remained.

Examining intra-individual (beta) microbiome diversity, we found significant differences across education and IMD groups in crude and adjusted NPMANOVA for Bray–Curtis dissimilarity (Education: crude *F*(3, 1425) = 1.71, *p* = 0.0004; adjusted *F*(3, 1425) = 1.75, *p* = 0.0004, IMD: crude *F*(4, 1671) = 1.37, *p* = 0.008; adjusted *F*(4, 1671) = 1.41, *p* = 0.006, permutations = 5000) and weighted UniFrac (Education: crude *F*(3, 1425) = 1.74, *p* = 0.0022; adjusted *F*(3, 1425) = 1.73, *p* = 0.0028, IMD: crude *F*(4, 1671) = 1.35, *p* = 0.03, adjusted *F*(4, 1671) = 1.38, *p* = 0.02). This suggests a difference in microbiota community composition between each education and IMD group, with the highest education level being the most dissimilar to the other groups. Difference in group centroids for both the crude and adjusted Income–Bray distance model neared the significance threshold; however, the latter is to be interpreted with caution as tests for homogeneity of variance also neared significance, as shown in Additional File 4. All *R*^2^ values were low; similar values were observed for covariates in adjusted models, as shown in Additional File 4. 

**Figure 2:**
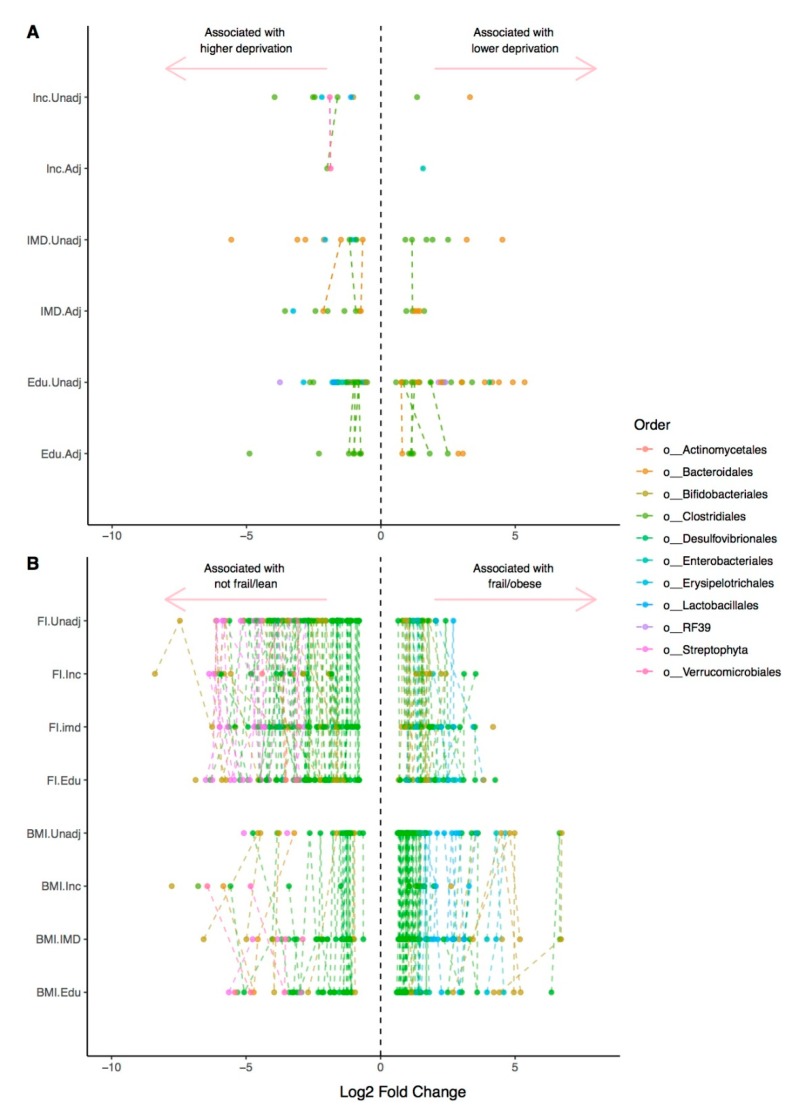
Differential abundance of OTUs with socioeconomic variables and covariates. DeSeq2 was used to calculate the differential abundance of OTUs in: (**A**). Between the lowest and highest levels of deprivation for education, income and the IMD, and in models adjusted for age, Body Mass Index (BMI), health deficit (FI) and diet (HEI); (**B**). Between lowest and highest levels of BMI and health deficit (FI), and in models adjusted for education (Edu), income (inc) and the Index of Multiple deprivation (imd). The phyla assigned to each denovo OTU is indicated. Dashed lines connect the same OTU ids in each hierarchical model; therefore, where there are no connecting lines, the associate was not observed in the corresponding model.

Of the 2126 OTUs considered for this analysis, we found a total of 76 unique OTUs that had a significant (FDR-adjusted *q* < 0.01) differential abundance between the lowest and highest levels of deprivation for all three SES variables, as shown in Figure 2A and Additional File 1.3–1.8, in unadjusted models.

Unadjusted, education models had the highest numbers of FDR-significant OTUs at 57 OTUs (17 in models adjusted for diet, age, BMI, and health deficit), followed by IMD (18 unadjusted, 15 in adjusted models), and income (10 unadjusted, 3 adjusted). To benchmark, comparisons were made to the associations of established correlates of the gut microbiome, BMI, and frailty, coded as factor variables using published thresholds, to be consistent with the SES measures. A total of 128 OTUs where observed to be differentially abundant (*q* < 0.01) in crude models between not frail and very frail individuals, and 90 OTUs between underweight and obese individuals. The number of associations for both traits diminished with adjustment for household income in particular: to 36 for frailty and 21 for body mass, as shown in Figure 2B and Additional File 1.9–1.16. 

**Figure 3:**
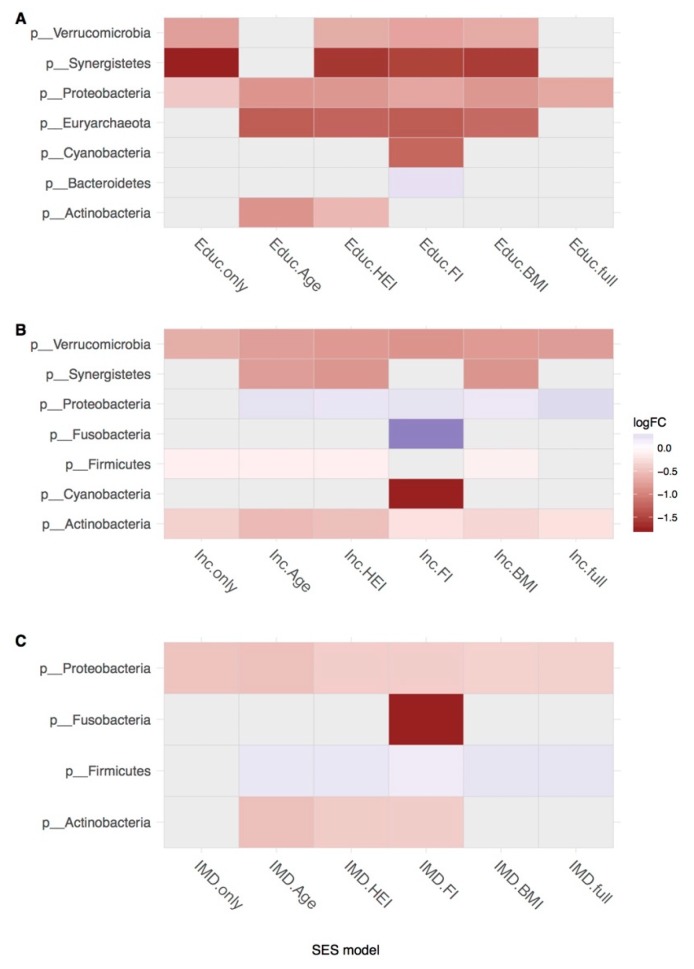
Differential abundance of phyla in hierarchical models. DeSeq2 was used to calculate the differential abundance of OTUs collapsed to phylum level in hierarchical models that were crude, adjusted for age, diet (HEI), health deficit (FI), and Body Mass Index (BMI) separately and together in three socioeconomic status (SES) measures: (**A**) education, (**B**) income, and (**C**) IMD. Only FDR-adjusted results significant above *q* < 0.05 are shown.

Results of OTU counts collapsed by phylum-level taxonomic assignment are shown in Figure 3. Interestingly, more associations with SES were found in models adjusted for individual covariates rather than alone or in models adjusted for all covariates together. Most of the differences at collapsed levels were observed across all three SES measures when adjusted for health deficit; results here and in previous studies within this cohort suggest that health deficit is a key correlate of the microbiota and therefore may have been suppressing the observed crude associations. IMD had the highest number of FDR-significant associations as family level; income had the least.

**Figure 4:**
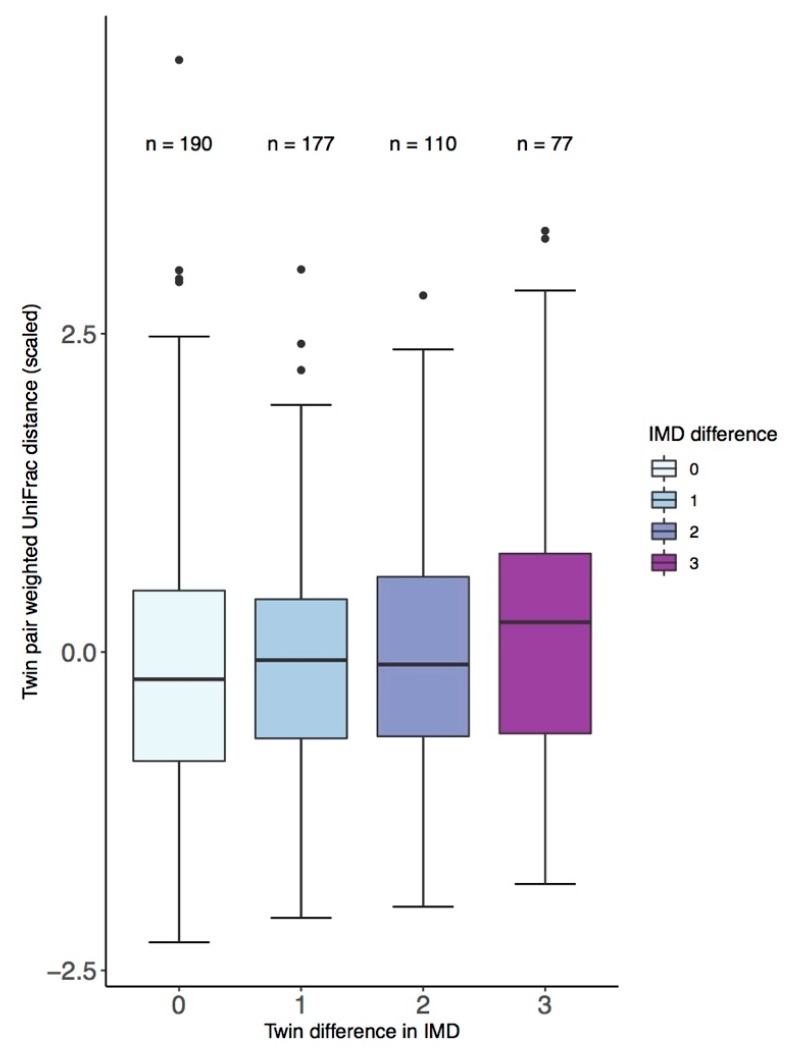
Between twin-pair weighted UniFrac distance and difference in quintile grouping of the Index of Multiple Deprivation (IMD).

Under a stringent definition of discordance, 22 twin pairs were discordant for education, 24 for income, and 48 for the IMD. There were no significant differences between alpha diversity of twins discordant for the SES measure. Within twin pairs, difference in IMD was significantly associated with Bray–Curtis dissimilarity (ANOVA *F*(3,546) = 2.9, *p* = 0.03) with factor-level significance, suggesting that the association was driven by twins with the greatest difference in IMD (*β* = 0.36, *p* = 0.008), as shown in Figure 4. Although only significant at factor-level, the same trend was seen for IMD and difference in weighted-UniFrac distance (*β* = 0.3, *p* = 0.02) as shown in Figure 4. No differences were observed between OTUs in discordant twin pairs. Analysis using a less stringent definition of discordance found similar but smaller differences, other than discordance for education where there was greater effect observed for the same de novo Clostridiales OTU observed in the more stringent analysis (logFC = 1.09, *q* = 0.019), as shown in Additional File 5. 

## 4. Discussion

Differences in health status by socioeconomic factors are well established, with an emerging focus on understanding the biological mechanisms underlying these relationships [44,45]. Current knowledge of the potential role of the microbiome in health inequalities is limited. To the best of our knowledge, this study is the first to examine the association between individual SES and the composition of the gut microbiome. We found associations between three different measures of SES and the composition of the gut microbiota in adulthood. Lower individual income was associated with reduced alpha diversity measures. Alpha diversity captures the evenness and richness of microbial composition within an individual, with lower alpha diversity generally seen in combination with worse health status [46]. In this study, adjustment for individual health status (BMI and proportion of health deficits) and healthy diet did not fully attenuate the association. This suggests that there are microbiome differences independent of these effects, although there are limits to the variation captured by the summary measures used. The associations found for area-level SES suggest that spatial- or community-level exposures may contribute to human gut microbiome composition, though the specific mechanisms through which these influence the microbiome requires further investigation.

Our study is consistent with one previous study that found an association between lower neighborhood-level SES and reduced alpha diversity in the colonic microbiota among forty-four healthy volunteers [27]. Our study expands upon these findings using a much larger sample with a wider range of health phenotypes and SES measured at the individual as well as area level. We similarly observed a greater abundance of *Bacteroides* with higher SES. Unlike this study, we generally observed a higher abundance of *Prevotella* within the least deprived groups. Miller et al. (2016) [27] posit that diet underlies differences in microbiota composition; our results indicate that whilst diet may be a mediating factor, specifically in taxa abundance, it does not completely explain variance of alpha and beta diversity associated with SES, at least when using the HEI as a summary of dietary intakes. 

The rich measurement of our cohort allowed us to control for potential mediators of the relationship between SES and the microbiome, specifically existing health deficits, BMI, and diet, which have previously been shown to be associated with the gut microbiome. Importantly, some SES associations remained upon adjustment for these factors, while the significance of diet and BMI in particular was attenuated by adjustment for SES. This suggests that SES may be an important confounding factor in microbiome studies that has not been previously accounted for and should be explored in future analysis. The lack of mediation of SES by current measures of diet or frailty may, in part, reflect the imperfect representation of the HEI and FI. Future studies should consider the limitation of such measures, for example, elements of diet not captured by the HEI, such as food quality, diversity, or a specific dietary constituent (e.g., meat intake or fiber) could be driving the different associations observed with SES. Such limitations could be overcome by utilizing dietary and health matched individuals in future comparisons. In addition to diet and health status, there are a range of SES-associated factors that were unexplored in this analysis and could also contribute to the associations observed, such as historical medication use, pet ownership [47], social relationships and stressors, and host environment [48,49].

There are some limitations of our data. Associations observed here were small in magnitude, but the range of SES may have been restricted by the TwinsUK volunteer sample. This cohort exhibits a volunteer bias and disproportionately comprises individuals with average income and educational levels, thus is both more socially homogeneous and more affluent on average than the general population [50]. Further population-based studies are needed, as those of differing cultural backgrounds may exhibit distinct socio-microbiome associations from this British cohort.

Caution should be applied when attempting to interpret OTU-level results, due to the limitations in 16S taxonomic assignment [51]. Bearing this in mind, some observations which may be indicative of community changes in differing socioeconomic groups warrant further investigation. Many of the identified OTU-level associations with SES markers are consistent with existing health associations for specific OTUs, summarized in Table 2. For example, lower levels of deprivation correlate with key health-associated bacteria such as *Faecalibacterium prausnitzii* and *Akkermansia muciniphila*, both proposed to reduce host inflammation [52,53], whereas higher levels of deprivation were associated with *Streptococcus anginosus*, which has been identified as a member of a dysbiotic microbiota [54]. OTUs assigned to paraphyletic taxa such as Clostridiales, Lachnospiraceae, and Ruminococcaceae, members of which are nearly ubiquitous in the gut assemblages [55], are both health-associated (e.g., *Prevotella* are reduced in obesity and diabetes associated microbiotas) and pathogenic (e.g., *Prevotella stercorea* is associated with carcinomas); therefore it is unsurprising that OTUs assigned to these taxa above species-level conflict in the direction of their relationship with socioeconomic variables.

A strength of the current study is the analysis of the gut microbiome in a large, well-characterized cohort and use of both individual and area-level SES measures. Building on the descriptive analysis provided here, future work could measure and explore important mediating factors to further elucidate the relationship between social factors and the microbiome. A further area of interest is determination of the influences of SES on microbiota composition throughout the human life course. Early SES and later life SES indicators are correlated [56], consistent with the low number of twin pairs discordant for SES; we hypothesize that adult SES measures are capturing cumulative exposures shaped by the social environment across the life course. As the colonization of the microbiome is influenced by early life exposures [57], these could be inducing founder-effects that drive differences in the microbiota between socioeconomic classes. Further longitudinal SES–microbiome studies should resolve these effects. In addition, geospatial analysis of the spatial structure of 16S data may elucidate how environmental or ecological factors contribute to the area-level differences identified here. 

## 5. Conclusions

This study found significant relationships between both area-based and individual socioeconomic factors, and stool 16S rRNA microbiota composition in a large sample of British twins. Associations were observed in models adjusting for health-related factors known to impact the microbiome, with suggestion that individual-level SES attenuate microbiota–health associations. These findings support the hypothesis that differences in the microbiota between social groups might be a novel biological mediator of the well-documented differences in health outcomes across the socioeconomic spectrum. 

## Figures and Tables

**Table 1:** Descriptive statistics by socioeconomic factors.

	Group	*n*	%MZ	(%Female)	Age μ	BMI μ	HEI μ	FI μ
IMD	***Q1***	336	57	89	60.35	26.9	59.53	0.19
	***Q2***	333	58	91	61.37	25.52	61.09	0.18
	***Q3***	334	55	90	61.35	26.3	60.22	0.19
	***Q4***	334	59	90	62.57	25.38	60.46	0.19
	***Q5***	335	54	93	63.7	25.53	60.46	0.18
	**Total**	1672	56	91	61.89	25.92	60.33	0.19
Education	***Q1***	224	50	91	68.92	27.18	58.92	0.24
	***Q2***	336	52	94	62.33	26.02	60.38	0.19
	***Q3***	486	57	89	61.2	25.81	60.32	0.19
	***Q4***	359	65	83	55.99	24.93	61.3	0.18
	**Total**	1426	57	89	61.48	25.87	60.34	0.2
Income	***Q1***	139	52	97	67.22	26.38	58.83	0.24
	***Q2***	203	45	93	64.62	26.32	59.55	0.21
	***Q3***	310	51	97	62.11	26.01	59.99	0.2
	***Q4***	147	52	86	60.53	25	61.72	0.16
	**Total**	799	50	92	63.35	25.97	59.99	0.2

IMD = Index of Multiple Deprivation, % MZ = % monozygotic; BMI = Body Mass Index; HEI = Healthy Eating Index; FI = Frailty Index; Q1 = most deprived category.

**Table 2:** Summary of taxa assigned to OTUs found to be differentially abundant between the most-deprived and least-deprived measures of socioeconomic status in at least two models. Only taxa with multiple OTUs assigned to it, or with multiple SES factors associated with it, and with *q*-value < 0.01 are discussed. OTUs relatively enriched in the least deprived compared to highest for each SES variable are indicated with (+); those enriched in the most deprived compared to the least indicated with (−); where multiple directions of association were observed, this is indicated with (+/−). The lowest assigned taxa level is indicated; number of OTUs assigned within this taxa at the lowest level is included. Categories refer to the current general consensus of the genera’s relationship with health where (A) generally positive health associations, (B) generally positive health associations, but opportunistic pathogens, (C) generally negative health associations, and (D) paraphyletic taxa/mixed consensus/not enough information. Where relationships are shown in red, they are contrary to the literature consensus on direction of health association.

Assigned Taxa	#	i. IMD	ii. IMD Adj	iii. Education	iv. Ed. Adj	v. Income	vi. Inc. Adj	Category	Health Associations
*Akkermansia muciniphila* (s)	1					−	−	A	Disrupts obesity-associated host metabolism [58]. Associated with reduction of inflammation [53].
*Anaerofustis* (g)	1			−	−			D	Decreases where soluble maize fiber used as supplement in adolescents [59,60]. Positively associated with infection in rabbit models [41].
*Anaerostipes* (g)	1			+				A	Butyrate producers that co-occur with other beneficial microbes [61]. Suggestion that excessive short chain fatty acids (SCFA) production promotes gastrointestinal symptoms associated with Rett syndrome [62].
*Bacteroides* (g) *Bacteroides coprophilus* (s)	6	−	+	+	+	+		B	Member of core microbiome [63]. Degrade dietary polysaccharides (glycans) generating beneficial SCFA [64]. Depletion associated with irritable bowel disorder IBD [65].
Barnesiellaceae (f)	4		+	+				C	Associated with the mucosal microbiota in patients with primary sclerosis cholangitis (PSC) [66] and potentially Parkinson’s disease [67]. Negatively associated with bacteraemia [68].
*Blautia* (g) *Blautia producta* (s)	4		−	−	+/−			D	Converts plant lignan precursors to enterolactone [69] which may explain its negative association with cancers, such as colorectal cancer (CRC) [70]. Species in genera have been linked with both obese and lean [71].
Christensenellaceae (f)	2			−				A	Implicated in human longevity [72] and better represented in lean and older individuals [73,74].
Clostridiales (o) Clostridiaceae (f) *Clostridium* (g)	13	+/−	−	+/−	+/−			D	Decreased abundance correlates with inflammatory bowel disease [75] and colorectal cancer [76]. Some members associated with promotion of obesity [69].
3	4			−			+	C	Phyla contains *Escherichia coli* and opportunistic pathogen *Klebsiella pneumoniae*. Associated with the development of ulcerative colitis [77]. Bacterially produced β-lactamases are responsible for pyogenic liver abscess [78].
*Eubacterium dolichum* (s)	2		−	−				C	Previously identified within this cohort as being associated with health deficit and higher visceral fat mass [3,79]. Associated with the “obese” gut microbiota [80,81].
*Faecalibacterium prausnitzii* (s)	1			+	+	+		A	Key butyrate producer to the colonic epithelium [82]. Negatively associated with pathogenesis of Crohn’s disease, inflammatory bowel disease, and prostate cancer [52,82,83,84]. Proposed mechanism of action is via production of the anti-inflammatory 15 kDa protein [52].
Lachnospiraceae (f) *Lachnospira* (g)	11	−	+/−	+/−	+	−	−	D	Murine models observe improvement to colonization resistance [85]. Induce hyperglycemia in obese mice [86] and negatively associated with resistant starch [87]. Implicated in deficit of caspase-1 which is suggested as having a protective effect in modulation of gut microbiota–brain pathways [88].
*Prevotella* (g) *Prevotella copri* (s) *Prevotella stercorea* (s)	5	+/−		+	+	+		B	Reduced in obese patients compared to healthy controls [89]. *P. copri* has been inferred in the pathogenesis of rheumatoid arthritis [90] and *P. stercorea* has been observed as associated with carcinoma-in-adenoma [91].
RF39 (o)	3			+/−				D	Correlates with *E. coli* under certain dietary conditions in bovine models [92].
Rikenellaceae (f)	2	+		+			−	A	Lower abundances associated with lean subjects [74]; depleted in patients with chronic HIV [93]. Target for microbial intervention in obesity management [94].
Rumminococcaceae (f) *Ruminococcus* (g) *Ruminococcus gnavus* (s)	11	+/−	+/−	+/−	+/−	−		D	Dominant and prevalent members of the non-individual specific gut microbiota [55,95]. Associated with diets high in resistant starch [96]. *R. gnavus* observed to be enriched in Crohn’s disease (CD) patients [97]. Keystone member of the mucus associated microbiome [98].
S24-7 (f)	3	−		+				D	Mouse models suggest role in collagen induced arthritis [99,100]—although the cited papers note different directions of effect.
Streptophyta (o) *Streptococcus* (g) *Streptococcus anginosus* (s)	4		−	−		−		C	*S. anginosus* a feature of negatively health-associated community assemblages [54]. Increased in colorectal cancer patients [101] and individuals suffering from non-alcoholic fatty liver disease [102].

## Supplementary Materials

The following are available online at http://www.mdpi.com/2076-2607/7/1/17/s1, Additional File 1: Supplementary tables; Additional File 2: SES and the microbiome: 1. Variables + Correlations—exploration of key analytical variables; Additional File 3: SES and the microbiome: 2. Alpha Diversity—R script and full output; Additional File 4: SES and the microbiome 3.1: Beta Diversity and Permanova—R script and full output; Additional File 5: SES and the microbiome 4: Twin discordance—R script and output for twin discordance analysis under the “less stringent” definition of twin discordance.
