# Supplementary material for: Socioeconomic Status and the Gut Microbiome: A TwinsUK Cohort Study
Source: Microorganisms. 2019 Jan 11;7(1):17. doi: 10.3390/microorganisms7010017 (PMC6351927; doi:10.3390/microorganisms7010017)
Supplement: Supplementary file 1 [file microorganisms-07-00017-s001.zip › Additional File 2.pdf]

SES & the microbiome: 1. Variables + Correlations

December 04, 2018

Socioeconomic variables

Education

Table 1: Highest education level (self-reported)

	n
No qualification	210
NVQ1/SVQ1	35
O-Level/GCSE/NVQ2/SVQ2/Scottish Intermediate	336
Scottish higher, NVQ3, city and qulds, Pitman	95
A Level, Scottish Advanced Higher	95
Higher Vocational training (e.g. Diploma, NVQ4, SVQ4)	296
University degree	252
Postgraduate degree (e.g. Masters or PhD), NVQ5, SVQ5	107

Table 2: Education - n per factor level

	n
1	245
2	336
3	486
4	359

IMD

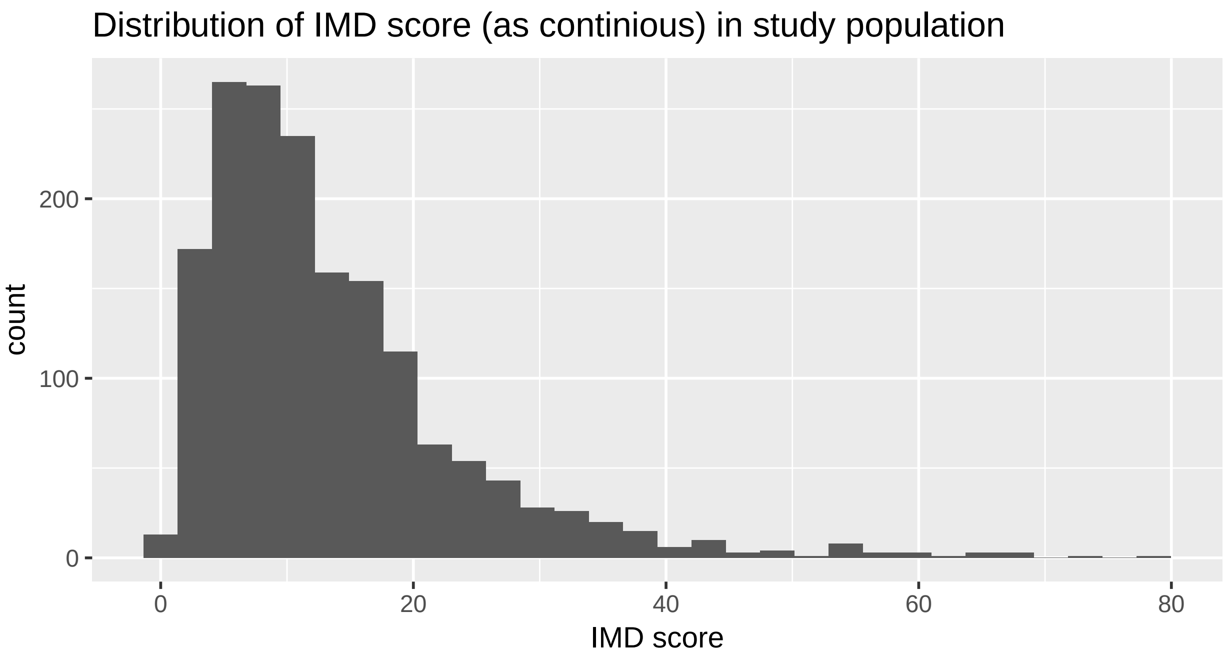

IMD set to quintiles recoded to match direction of other SES variables: 1= most deprived, 5 = least deprived

Table 3: IMD (quintiles) - n

	n
1	336
2	333
3	334
4	334
5	335

Income

Table 4: Income

	n
< £5000	14
£5000 - £9999	49
£10,000 - 14,999	76
£15,000 - £19,999	90
£20,000 - £24,999	113
£25,000 - £49,999	310
£50,000<	147

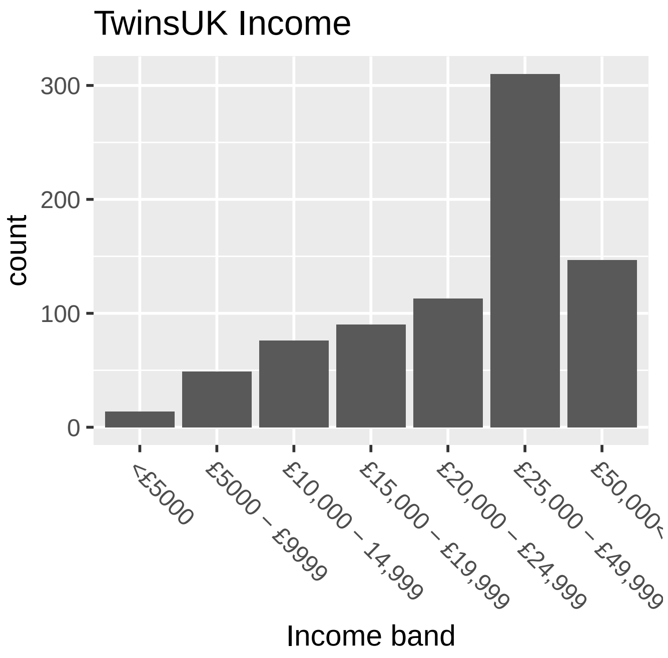

Income grouped into four factors

Table 5: Income (4 factors)

	n
< £14,999	139
£15,000 - £24,999	203
£25,000 - £49,999	310
£50,000<	147

Metadata

Table 6: Covariates

	n	mean	sd	median	min	max
BMI	1672	25.9249703	4.7119462	25.1690159	16.16647	47.0782509
Age	1672	61.8881579	11.1859878	63.0000000	19.00000	90.0000000
HEI	1672	60.3313397	10.0105999	60.0000000	23.00000	93.0000000
FI	1672	0.1870248	0.1244325	0.1580645	0.00000	0.7068965

Table 7: Gender

	n
F	1518
M	154

Table 8: Zygosity

	n
DZ	729
MZ	943

Table 9: Ethnic Origin

		Freq
2	Asian	7
3	Black	10
4	Chinese	0
5	Mixed	9
6	Other	2
7	White	1631
1	No data	13

Correlation of socio-econmic factors

Correlation of SES factors with covariates

```
ses_microbiome$IMD5f <- as.factor(ses_microbiome$IMD5f)
ses_microbiome$eduff <- as.factor(ses_microbiome$eduff)
ses_microbiome$Income4F <- as.factor(ses_microbiome$Income4F)

toscale <- c("Age","HEI", "BMI")

ses_microbiome[,which(names(ses_microbiome)%in%toscale)] <- sapply(ses_microbiome[,whic
 scale(x, center=T)
})

ses_microbiome$FIsqrt <- sqrt(ses_microbiome$FI)

ses_microbiome1 <- ses_microbiome[complete.cases(ses_microbiome$IMD5f),]
```

IMD quantiles vs Covars

```
IMD.mods <- apply(ses_microbiome1[c("Age", "BMI", "FIsqrt", "HEI")],2, function(x){
  a<- lm(x ~ IMD5f, data=ses_microbiome1)
  b<-summary(a)
  c <- anova(a)
 return(list(b,c))
})


IMD.mods
```

```
## $Age
## $Age[[1]]
##
## Call:
## lm(formula = x ~ IMD5f, data = ses_microbiome1)
##
## Residuals:
##     Min      1Q  Median      3Q     Max
## -3.8947 -0.5100  0.1158  0.6522  2.4715
##
## Coefficients:
##             Estimate Std. Error t value Pr(>|t|)
## (Intercept) -0.13714    0.05433  -2.524 0.011693 *
## IMD5f2       0.09076    0.07701   1.179 0.238757
```

```
## IMD5f3       0.09788    0.07695   1.272 0.203538
## IMD5f4       0.19772    0.07695   2.569 0.010273 *
## IMD5f5       0.29951    0.07689   3.895 0.000102 ***
## ---
## Signif. codes:  0 '***' 0.001 '**' 0.01 '*' 0.05 '.' 0.1 ' ' 1
##
## Residual standard error: 0.9959 on 1667 degrees of freedom
## Multiple R-squared:  0.01054,    Adjusted R-squared:  0.008163
## F-statistic: 4.438 on 4 and 1667 DF,  p-value: 0.001432
##
##
## $Age[[2]]
## Analysis of Variance Table
##
## Response: x
##             Df  Sum Sq Mean Sq F value   Pr(>F)
## IMD5f        4   17.61  4.4020  4.4383 0.001432 **
## Residuals 1667 1653.39  0.9918
## ---
## Signif. codes:  0 '***' 0.001 '**' 0.01 '*' 0.05 '.' 0.1 ' ' 1
##
##
## $BMI
## $BMI[[1]]
##
## Call:
## lm(formula = x ~ IMD5f, data = ses_microbiome1)
##
## Residuals:
##     Min      1Q  Median      3Q     Max
## -1.9888 -0.6969 -0.1305  0.5137  4.6056
##
## Coefficients:
##             Estimate Std. Error t value Pr(>|t|)
## (Intercept)  0.20672    0.05420   3.814 0.000142 ***
## IMD5f2      -0.29283    0.07682  -3.812 0.000143 ***
## IMD5f3      -0.12740    0.07676  -1.660 0.097155 .
## IMD5f4      -0.32303    0.07676  -4.208 2.71e-05 ***
## IMD5f5      -0.29157    0.07670  -3.801 0.000149 ***
## ---
## Signif. codes:  0 '***' 0.001 '**' 0.01 '*' 0.05 '.' 0.1 ' ' 1
##
## Residual standard error: 0.9934 on 1667 degrees of freedom
## Multiple R-squared:  0.01548,    Adjusted R-squared:  0.01311
## F-statistic: 6.551 on 4 and 1667 DF,  p-value: 3.143e-05
```

```
##
##
## $BMI[[2]]
## Analysis of Variance Table
##
## Response: x
##             Df  Sum Sq Mean Sq F value    Pr(>F)
## IMD5f        4   25.86  6.4648  6.5507 3.143e-05 ***
## Residuals 1667 1645.14  0.9869
## ---
## Signif. codes:  0 '***' 0.001 '**' 0.01 '*' 0.05 '.' 0.1 ' ' 1
##
##
## $FIsqrt
## $FIsqrt[[1]]
##
## Call:
## lm(formula = x ~ IMD5f, data = ses_microbiome1)
##
## Residuals:
##      Min       1Q   Median       3Q      Max
## -0.41657 -0.09821 -0.01023  0.09333  0.43027
##
## Coefficients:
##               Estimate Std. Error t value Pr(>|t|)
## (Intercept)  0.4104986  0.0079149  51.864   <2e-16 ***
## IMD5f2      -0.0161334  0.0112185  -1.438    0.151
## IMD5f3       0.0060714  0.0112101   0.542    0.588
## IMD5f4       0.0001363  0.0112101   0.012    0.990
## IMD5f5      -0.0055669  0.0112017  -0.497    0.619
## ---
## Signif. codes:  0 '***' 0.001 '**' 0.01 '*' 0.05 '.' 0.1 ' ' 1
##
## Residual standard error: 0.1451 on 1667 degrees of freedom
## Multiple R-squared:  0.002656,   Adjusted R-squared:  0.0002624
## F-statistic:  1.11 on 4 and 1667 DF,  p-value: 0.3503
##
##
## $FIsqrt[[2]]
## Analysis of Variance Table
##
## Response: x
##             Df Sum Sq  Mean Sq F value Pr(>F)
## IMD5f        4  0.093 0.023357  1.1096 0.3503
## Residuals 1667 35.088 0.021049
```

```
##
##
## $HEI
## $HEI[[1]]
##
## Call:
## lm(formula = x ~ IMD5f, data = ses_microbiome1)
##
## Residuals:
##     Min      1Q  Median      3Q     Max
## -3.7182 -0.6526 -0.0361  0.6771  3.2744
##
## Coefficients:
##             Estimate Std. Error t value Pr(>|t|)
## (Intercept) -0.07978    0.05455  -1.462    0.144
## IMD5f2       0.15587    0.07732   2.016    0.044 *
## IMD5f3       0.06881    0.07726   0.891    0.373
## IMD5f4       0.09214    0.07726   1.193    0.233
## IMD5f5       0.08276    0.07721   1.072    0.284
## ---
## Signif. codes:  0 '***' 0.001 '**' 0.01 '*' 0.05 '.' 0.1 ' ' 1
##
## Residual standard error: 1 on 1667 degrees of freedom
## Multiple R-squared:  0.00249,    Adjusted R-squared:  9.643e-05
## F-statistic:  1.04 on 4 and 1667 DF,  p-value: 0.385
##
##
## $HEI[[2]]
## Analysis of Variance Table
##
## Response: x
##             Df  Sum Sq Mean Sq F value Pr(>F)
## IMD5f        4    4.16  1.0402  1.0403  0.385
## Residuals 1667 1666.84  0.9999
```

```
chisq.test(ses_microbiome1$IMD5f, ses_microbiome1$Ethnic_Origin)
```

```
##
##  Pearson's Chi-squared test
##
## data:  ses_microbiome1$IMD5f and ses_microbiome1$Ethnic_Origin
## X-squared = 21.301, df = 20, p-value = 0.3796
```

Education quantiles vs Covars

```
ses_microbiome2 <- ses_microbiome[complete.cases(ses_microbiome$eduff),]

edu.mods <- apply(ses_microbiome2[c("Age", "BMI", "FIsqrt", "HEI")],2, function(x){
  a<- lm(x ~ eduff, data=ses_microbiome2)
  b<-summary(a)
  c <- anova(a)
 return(list(b,c))
})

edu.mods
```

```
## $Age
## $Age[[1]]
##
## Call:
## lm(formula = x ~ eduff, data = ses_microbiome2)
##
## Residuals:
##     Min      1Q  Median      3Q     Max
## -3.8739 -0.5356  0.0712  0.6076  2.5933
##
## Coefficients:
##             Estimate Std. Error t value Pr(>|t|)
## (Intercept)  0.62885    0.06067  10.365  < 2e-16 ***
## eduff2      -0.58905    0.07978  -7.384 2.61e-13 ***
## eduff3      -0.69004    0.07441  -9.274  < 2e-16 ***
## eduff4      -1.15598    0.07869 -14.690  < 2e-16 ***
## ---
## Signif. codes:  0 '***' 0.001 '**' 0.01 '*' 0.05 '.' 0.1 ' ' 1
##
## Residual standard error: 0.9496 on 1422 degrees of freedom
## Multiple R-squared:  0.1332, Adjusted R-squared:  0.1314
## F-statistic: 72.87 on 3 and 1422 DF,  p-value: < 2.2e-16
##
##
## $Age[[2]]
## Analysis of Variance Table
##
## Response: x
##             Df  Sum Sq Mean Sq F value    Pr(>F)
## eduff        3  197.13  65.710  72.868 < 2.2e-16 ***
## Residuals 1422 1282.30   0.902
```

```
## ---
## Signif. codes:  0 '***' 0.001 '**' 0.01 '*' 0.05 '.' 0.1 ' ' 1
##
##
## $BMI
## $BMI[[1]]
##
## Call:
## lm(formula = x ~ eduff, data = ses_microbiome2)
##
## Residuals:
##     Min      1Q  Median      3Q     Max
## -2.0913 -0.6713 -0.1316  0.5580  4.7000
##
## Coefficients:
##             Estimate Std. Error t value Pr(>|t|)
## (Intercept)  0.26556    0.06167   4.306 1.78e-05 ***
## eduff2      -0.24525    0.08110  -3.024 0.002538 **
## eduff3      -0.28968    0.07564  -3.830 0.000134 ***
## eduff4      -0.47628    0.07999  -5.954 3.29e-09 ***
## ---
## Signif. codes:  0 '***' 0.001 '**' 0.01 '*' 0.05 '.' 0.1 ' ' 1
##
## Residual standard error: 0.9653 on 1422 degrees of freedom
## Multiple R-squared:  0.02464,    Adjusted R-squared:  0.02258
## F-statistic: 11.97 on 3 and 1422 DF,  p-value: 9.614e-08
##
##
## $BMI[[2]]
## Analysis of Variance Table
##
## Response: x
##             Df  Sum Sq Mean Sq F value    Pr(>F)
## eduff        3   33.47 11.1574  11.973 9.614e-08 ***
## Residuals 1422 1325.09  0.9318
## ---
## Signif. codes:  0 '***' 0.001 '**' 0.01 '*' 0.05 '.' 0.1 ' ' 1
##
##
## $FIsqrt
## $FIsqrt[[1]]
##
## Call:
## lm(formula = x ~ eduff, data = ses_microbiome2)
##
```

```
## Residuals:
##      Min       1Q   Median       3Q      Max
## -0.41462 -0.09439 -0.00544  0.09051  0.39921
##
## Coefficients:
##              Estimate Std. Error t value Pr(>|t|)
## (Intercept)  0.469937   0.008978  52.346  < 2e-16 ***
## eduff2      -0.051240   0.011805  -4.340 1.52e-05 ***
## eduff3      -0.055316   0.011010  -5.024 5.70e-07 ***
## eduff4      -0.068847   0.011645  -5.912 4.22e-09 ***
## ---
## Signif. codes:  0 '***' 0.001 '**' 0.01 '*' 0.05 '.' 0.1 ' ' 1
##
## Residual standard error: 0.1405 on 1422 degrees of freedom
## Multiple R-squared:  0.02601,    Adjusted R-squared:  0.02396
## F-statistic: 12.66 on 3 and 1422 DF,  p-value: 3.616e-08
##
##
## $FIsqrt[[2]]
## Analysis of Variance Table
##
## Response: x
##             Df  Sum Sq  Mean Sq F value    Pr(>F)
## eduff        3  0.7499 0.249983   12.66 3.616e-08 ***
## Residuals 1422 28.0787 0.019746
## ---
## Signif. codes:  0 '***' 0.001 '**' 0.01 '*' 0.05 '.' 0.1 ' ' 1
##
##
## $HEI
## $HEI[[1]]
##
## Call:
## lm(formula = x ~ eduff, data = ses_microbiome2)
##
## Residuals:
##     Min      1Q  Median      3Q     Max
## -3.5880 -0.6778 -0.0321  0.6672  3.3047
##
## Coefficients:
##             Estimate Std. Error t value Pr(>|t|)
## (Intercept) -0.14115    0.06337  -2.227  0.02609 *
## eduff2       0.14640    0.08334   1.757  0.07917 .
## eduff3       0.14011    0.07772   1.803  0.07164 .
## eduff4       0.23744    0.08220   2.888  0.00393 **
```

```
## ---
## Signif. codes:  0 '***' 0.001 '**' 0.01 '*' 0.05 '.' 0.1 ' ' 1
##
## Residual standard error: 0.992 on 1422 degrees of freedom
## Multiple R-squared:  0.005839,   Adjusted R-squared:  0.003742
## F-statistic: 2.784 on 3 and 1422 DF,  p-value: 0.03965
##
##
## $HEI[[2]]
## Analysis of Variance Table
##
## Response: x
##             Df  Sum Sq Mean Sq F value  Pr(>F)
## eduff        3    8.22 2.73945   2.784 0.03965 *
## Residuals 1422 1399.24 0.98399
## ---
## Signif. codes:  0 '***' 0.001 '**' 0.01 '*' 0.05 '.' 0.1 ' ' 1
```

```
chisq.test(ses_microbiome2$eduff, ses_microbiome2$Ethnic_Origin)
```

```
##
##  Pearson's Chi-squared test
##
## data:  ses_microbiome2$eduff and ses_microbiome2$Ethnic_Origin
## X-squared = 20.935, df = 15, p-value = 0.1389
```

Income quantiles vs Covars

```
ses_microbiome3 <- ses_microbiome[complete.cases(ses_microbiome$Income4F),]

inc.mods <- apply(ses_microbiome3[c("Age", "BMI", "FIsqrt", "HEI")],2, function(x){
  a<- lm(x ~ Income4F, data=ses_microbiome3)
  b<-summary(a)
  c <- anova(a)
 return(list(b,c))
})

inc.mods
```

```
## $Age
## $Age[[1]]
##
## Call:
## lm(formula = x ~ Income4F, data = ses_microbiome3)
```

```
##
## Residuals:
##      Min       1Q   Median       3Q      Max
## -2.79062 -0.43486  0.04196  0.52629  2.18750
##
## Coefficients:
##             Estimate Std. Error t value Pr(>|t|)
## (Intercept)  0.47628    0.07119   6.690 4.20e-11 ***
## Income4F2   -0.23244    0.09240  -2.516   0.0121 *
## Income4F3   -0.45619    0.08567  -5.325 1.32e-07 ***
## Income4F4   -0.59764    0.09930  -6.019 2.68e-09 ***
## ---
## Signif. codes:  0 '***' 0.001 '**' 0.01 '*' 0.05 '.' 0.1 ' ' 1
##
## Residual standard error: 0.8393 on 795 degrees of freedom
## Multiple R-squared:  0.05458,    Adjusted R-squared:  0.05101
## F-statistic:  15.3 on 3 and 795 DF,  p-value: 1.099e-09
##
##
## $Age[[2]]
## Analysis of Variance Table
##
## Response: x
##            Df Sum Sq Mean Sq F value    Pr(>F)
## Income4F    3  32.33 10.7770  15.299 1.099e-09 ***
## Residuals 795 560.02  0.7044
## ---
## Signif. codes:  0 '***' 0.001 '**' 0.01 '*' 0.05 '.' 0.1 ' ' 1
##
##
## $BMI
## $BMI[[1]]
##
## Call:
## lm(formula = x ~ Income4F, data = ses_microbiome3)
##
## Residuals:
##     Min      1Q  Median      3Q     Max
## -2.0888 -0.6781 -0.1760  0.5687  4.1513
##
## Coefficients:
##             Estimate Std. Error t value Pr(>|t|)
## (Intercept)  0.09606    0.08121   1.183    0.237
## Income4F2   -0.01272    0.10540  -0.121    0.904
## Income4F3   -0.07826    0.09773  -0.801    0.424
```

```
## Income4F4   -0.29234    0.11327  -2.581    0.010 *
## ---
## Signif. codes:  0 '***' 0.001 '**' 0.01 '*' 0.05 '.' 0.1 ' ' 1
##
## Residual standard error: 0.9574 on 795 degrees of freedom
## Multiple R-squared:  0.01139,    Adjusted R-squared:  0.007657
## F-statistic: 3.053 on 3 and 795 DF,  p-value: 0.02785
##
##
## $BMI[[2]]
## Analysis of Variance Table
##
## Response: x
##            Df Sum Sq Mean Sq F value  Pr(>F)
## Income4F    3   8.39 2.79808  3.0525 0.02785 *
## Residuals 795 728.73 0.91664
## ---
## Signif. codes:  0 '***' 0.001 '**' 0.01 '*' 0.05 '.' 0.1 ' ' 1
##
##
## $FIsqrt
## $FIsqrt[[1]]
##
## Call:
## lm(formula = x ~ Income4F, data = ses_microbiome3)
##
## Residuals:
##      Min       1Q   Median       3Q      Max
## -0.43123 -0.09664 -0.00825  0.09635  0.39132
##
## Coefficients:
##             Estimate Std. Error t value Pr(>|t|)
## (Intercept)  0.46285    0.01222  37.869  < 2e-16 ***
## Income4F2   -0.03162    0.01586  -1.993   0.0466 *
## Income4F3   -0.03627    0.01471  -2.466   0.0139 *
## Income4F4   -0.08431    0.01705  -4.945 9.28e-07 ***
## ---
## Signif. codes:  0 '***' 0.001 '**' 0.01 '*' 0.05 '.' 0.1 ' ' 1
##
## Residual standard error: 0.1441 on 795 degrees of freedom
## Multiple R-squared:  0.03082,    Adjusted R-squared:  0.02717
## F-statistic: 8.428 on 3 and 795 DF,  p-value: 1.616e-05
##
##
## $FIsqrt[[2]]
```

```
## Analysis of Variance Table
##
## Response: x
##            Df Sum Sq  Mean Sq F value    Pr(>F)
## Income4F    3  0.525 0.175005  8.4279 1.616e-05 ***
## Residuals 795 16.508 0.020765
## ---
## Signif. codes:  0 '***' 0.001 '**' 0.01 '*' 0.05 '.' 0.1 ' ' 1
##
##
## $HEI
## $HEI[[1]]
##
## Call:
## lm(formula = x ~ Income4F, data = ses_microbiome3)
##
## Residuals:
##     Min      1Q  Median      3Q     Max
## -2.6959 -0.6767  0.0013  0.6363  3.3130
##
## Coefficients:
##             Estimate Std. Error t value Pr(>|t|)
## (Intercept) -0.14952    0.08078  -1.851   0.0645 .
## Income4F2    0.07115    0.10485   0.679   0.4976
## Income4F3    0.11513    0.09722   1.184   0.2366
## Income4F4    0.28767    0.11267   2.553   0.0109 *
## ---
## Signif. codes:  0 '***' 0.001 '**' 0.01 '*' 0.05 '.' 0.1 ' ' 1
##
## Residual standard error: 0.9524 on 795 degrees of freedom
## Multiple R-squared:  0.009085,   Adjusted R-squared:  0.005346
## F-statistic:  2.43 on 3 and 795 DF,  p-value: 0.06405
##
##
## $HEI[[2]]
## Analysis of Variance Table
##
## Response: x
##            Df Sum Sq Mean Sq F value  Pr(>F)
## Income4F    3   6.61 2.20368  2.4296 0.06405 .
## Residuals 795 721.08 0.90703
## ---
## Signif. codes:  0 '***' 0.001 '**' 0.01 '*' 0.05 '.' 0.1 ' ' 1
```

```r
chisq.test(ses_microbiome3$Income4F, ses_microbiome3$Income4F)
```

```
##
##  Pearson's Chi-squared test
##
## data:  ses_microbiome3$Income4F and ses_microbiome3$Income4F
## X-squared = 2397, df = 9, p-value < 2.2e-16
```
