# Supplementary material for: Socioeconomic Status and the Gut Microbiome: A TwinsUK Cohort Study
Source: Microorganisms. 2019 Jan 11;7(1):17. doi: 10.3390/microorganisms7010017 (PMC6351927; doi:10.3390/microorganisms7010017)
Supplement: Supplementary file 1 [file microorganisms-07-00017-s001.zip › Additional File 3.pdf]

SES & the microbiome: 2. Alpha Diversity

December 04, 2018

Data

```r
setwd("~/Documents/PhD/Data_+_analy/SES/Data")

alphas <- read.table("~/Documents/PhD/Data_+_analy/SES/Data/alphas_phyloseq_ALLRAWOTUSINCSINGS.txt")
m1 <- read.csv("~/Documents/PhD/Data_+_analy/SES/Data/mapping_ses_correctpcdes041218.csv")
m1$eduff <- as.factor(m1$eduff)
m1$IMD5f <- as.factor(m1$IMD5f)
m1$Income4F <- as.factor(m1$Income4F)

mapalph <- merge(m1, alphas, by.x = "SequencingSpecificName",
    by.y = "row.names")

alphas <- mapalph[, c("Chao1", "Shannon", "Simpson")]
alphas <- sapply(alphas, function(x) {
    scale(x, center = T)
})
alphas <- as.data.frame(alphas)

mapalph$FIsqrt <- sqrt(mapalph$FI)

covars <- mapalph[, c("BMI", "HEI", "FIsqrt", "Age", "zyg")]
covars <- as.data.frame(apply(mapalph[, c("BMI", "HEI", "FIsqrt",
    "Age", "zyg")], 2, function(x) {
    scale(x, center = T)
}))
mapalph[, c("BMI", "HEI", "FIsqrt", "Age", "zyg")] <- covars
```

```r
# Function for standarising coefficients

stdCoef.merMod <- function(object) {
    sdy <- sd(getME(object, "y"))
    sdx <- apply(getME(object, "X"), 2, sd)
    sc <- fixef(object) * sdx/sdy
    se.fixef <- coef(summary(object))[, "Std. Error"]
    se <- se.fixef * sdx/sdy
    return(data.frame(stdcoef = sc, stdse = se))
}
```

Covariate model

```r
require(lmerTest)
nonses <- apply(alphas, 2, function(x) {
    mod <- lmer(x ~ Library_sizelog10 + BMI + HEI + FIsqrt +
        Age + zyg + (1 | HEIv) + (1 | Frailty_Index_Vers) + (1 |
```

```r
        SequencingRunID) + (1 | ExtractionPlateLoadedby) + (1 |
        Extractedby) + (1 | CollectionMethod), REML = F, data = mapalph)
    beta <- stdCoef.merMod(mod)
    sum <- summary(mod)
    list <- list(sum, beta)
    return(list)
})

nonses
```

```
## $Chao1
## $Chao1[[1]]
## Linear mixed model fit by maximum likelihood . t-tests use
##   Satterthwaite's method [lmerModLmerTest]
## Formula: x ~ Library_sizelog10 + BMI + HEI + FIsqrt + Age + zyg + (1 |
##     HEIv) + (1 | Frailty_Index_Vers) + (1 | SequencingRunID) +
##     (1 | ExtractionPlateLoadedby) + (1 | Extractedby) + (1 |
##     CollectionMethod)
##    Data: mapalph
##
##      AIC      BIC   logLik deviance df.resid
##   3329.1   3405.0  -1650.6   3301.1     1658
##
## Scaled residuals:
##     Min      1Q  Median      3Q     Max
## -3.4233 -0.5525 -0.0972  0.4014  7.5556
##
## Random effects:
##  Groups                  Name        Variance  Std.Dev.
##  SequencingRunID         (Intercept) 0.2085536 0.45668
##  ExtractionPlateLoadedby (Intercept) 0.0337901 0.18382
##  CollectionMethod        (Intercept) 0.0013328 0.03651
##  Extractedby             (Intercept) 0.0000000 0.00000
##  Frailty_Index_Vers      (Intercept) 0.0002214 0.01488
##  HEIv                    (Intercept) 0.0000000 0.00000
##  Residual                            0.3935009 0.62730
## Number of obs: 1672, groups:
## SequencingRunID, 33; ExtractionPlateLoadedby, 10; CollectionMethod, 2; Extractedby, 2; Frailty_Index_Ve
##
## Fixed effects:
##                    Estimate Std. Error         df t value Pr(>|t|)
## (Intercept)       -1.353e+01  4.951e-01  1.151e+03 -27.340   <2e-16 ***
## Library_sizelog10  2.748e+00  9.855e-02  1.596e+03  27.884   <2e-16 ***
## BMI               -2.107e-03  1.629e-02  1.083e+03  -0.129    0.897
## HEI               -1.729e-02  1.576e-02  1.640e+03  -1.097    0.273
## FIsqrt            -1.721e-02  1.739e-02  1.366e+01  -0.990    0.339
## Age               -9.796e-04  1.654e-02  1.231e+02  -0.059    0.953
## zyg               -1.639e-02  1.601e-02  1.649e+03  -1.024    0.306
## ---
## Signif. codes:  0 '***' 0.001 '**' 0.01 '*' 0.05 '.' 0.1 ' ' 1
##
## Correlation of Fixed Effects:
##             (Intr) Lbr_10 BMI    HEI    FIsqrt Age
## Lbrry_szl10 -0.975
```

```
## BMI          0.007 -0.006
## HEI          0.024 -0.022  0.102
## FIsqrt      -0.006  0.003 -0.254  0.065
## Age         -0.057  0.063  0.004  0.033 -0.248
## zyg         -0.021  0.020 -0.033 -0.023 -0.004 -0.096
##
## $Chao1[[2]]
##                         stdcoef      stdse
## (Intercept)        0.0000000000 0.00000000
## Library_sizelog10  0.5167372423 0.01853180
## BMI               -0.0021072558 0.01629348
## HEI               -0.0172913795 0.01575775
## FIsqrt            -0.0172148054 0.01738802
## Age               -0.0009795809 0.01654441
## zyg               -0.0163942771 0.01601182
##
##
## $Shannon
## $Shannon[[1]]
## Linear mixed model fit by maximum likelihood . t-tests use
##   Satterthwaite's method [lmerModLmerTest]
## Formula: x ~ Library_sizelog10 + BMI + HEI + FIsqrt + Age + zyg + (1 |
##     HEIv) + (1 | Frailty_Index_Vers) + (1 | SequencingRunID) +
##     (1 | ExtractionPlateLoadedby) + (1 | Extractedby) + (1 |
##     CollectionMethod)
##    Data: mapalph
##
##      AIC      BIC   logLik deviance df.resid
##   4662.7   4738.6  -2317.3   4634.7     1658
##
## Scaled residuals:
##     Min      1Q  Median      3Q     Max
## -5.6123 -0.5088  0.1221  0.6951  2.2889
##
## Random effects:
##  Groups                  Name        Variance  Std.Dev.
##  SequencingRunID         (Intercept) 1.339e-02 1.157e-01
##  ExtractionPlateLoadedby (Intercept) 1.781e-14 1.335e-07
##  CollectionMethod        (Intercept) 0.000e+00 0.000e+00
##  Extractedby             (Intercept) 1.487e-02 1.219e-01
##  Frailty_Index_Vers      (Intercept) 2.209e-02 1.486e-01
##  HEIv                    (Intercept) 0.000e+00 0.000e+00
##  Residual                            9.228e-01 9.606e-01
## Number of obs: 1672, groups:
## SequencingRunID, 33; ExtractionPlateLoadedby, 10; CollectionMethod, 2; Extractedby, 2; Frailty_Index_Ve[illegible]
##
## Fixed effects:
##                    Estimate Std. Error         df t value Pr(>|t|)
## (Intercept)         0.30109    0.67221  346.07220   0.448 0.654496
## Library_sizelog10  -0.05618    0.13491  479.23554  -0.416 0.677292
## BMI                -0.10847    0.02496 1666.51717  -4.346 1.47e-05 ***
## HEI                 0.08261    0.02393 1667.19602   3.452 0.000571 ***
## FIsqrt             -0.18612    0.02930 1173.59223  -6.353 3.02e-10 ***
## Age                 0.13190    0.02582 1580.33395   5.108 3.66e-07 ***
```

```
## zyg                  0.02220    0.02399 1645.76759   0.925 0.354961
## ---
## Signif. codes:  0 '***' 0.001 '**' 0.01 '*' 0.05 '.' 0.1 ' ' 1
##
## Correlation of Fixed Effects:
##             (Intr) Lbr_10 BMI    HEI    FIsqrt Age
## Lbrry_szl10 -0.976
## BMI          0.005 -0.006
## HEI          0.013 -0.010  0.100
## FIsqrt      -0.006  0.012 -0.287  0.073
## Age         -0.045  0.045  0.039  0.022 -0.331
## zyg         -0.055  0.052 -0.032 -0.029 -0.018 -0.083
##
## $Shannon[[2]]
##                       stdcoef      stdse
## (Intercept)        0.00000000 0.00000000
## Library_sizelog10 -0.01056405 0.02536900
## BMI               -0.10846862 0.02495752
## HEI                0.08260612 0.02393262
## FIsqrt            -0.18612294 0.02929772
## Age                0.13190147 0.02582450
## zyg                0.02219831 0.02399114
##
##
## $Simpson
## $Simpson[[1]]
## Linear mixed model fit by maximum likelihood . t-tests use
##   Satterthwaite's method [lmerModLmerTest]
## Formula: x ~ Library_sizelog10 + BMI + HEI + FIsqrt + Age + zyg + (1 |
##     HEIv) + (1 | Frailty_Index_Vers) + (1 | SequencingRunID) +
##     (1 | ExtractionPlateLoadedby) + (1 | Extractedby) + (1 |
##     CollectionMethod)
##    Data: mapalph
##
##      AIC      BIC   logLik deviance df.resid
##   4732.9   4808.8  -2352.4   4704.9     1658
##
## Scaled residuals:
##      Min       1Q   Median       3Q      Max
## -10.8113  -0.1743   0.2644   0.5313   1.1480
##
## Random effects:
##  Groups                  Name        Variance  Std.Dev.
##  SequencingRunID         (Intercept) 2.467e-03 4.966e-02
##  ExtractionPlateLoadedby (Intercept) 2.434e-15 4.934e-08
##  CollectionMethod        (Intercept) 1.834e-14 1.354e-07
##  Extractedby             (Intercept) 2.493e-02 1.579e-01
##  Frailty_Index_Vers      (Intercept) 8.704e-03 9.330e-02
##  HEIv                    (Intercept) 0.000e+00 0.000e+00
##  Residual                            9.700e-01 9.849e-01
## Number of obs: 1672, groups:
## SequencingRunID, 33; ExtractionPlateLoadedby, 10; CollectionMethod, 2; Extractedby, 2; Frailty_Index_Ve
##
## Fixed effects:
```

```
##                      Estimate Std. Error        df t value Pr(>|t|)
## (Intercept)        3.263e-01  6.555e-01  1.430e+02   0.498 0.619357
## Library_sizelog10 -4.677e-02  1.317e-01  1.578e+02  -0.355 0.722926
## BMI               -6.425e-02  2.547e-02  1.642e+03  -2.523 0.011733 *
## HEI                5.509e-02  2.446e-02  1.670e+03   2.252 0.024433 *
## FIsqrt            -1.144e-01  2.946e-02  4.010e+02  -3.883 0.000121 ***
## Age                7.501e-02  2.614e-02  1.104e+03   2.870 0.004189 **
## zyg                9.289e-03  2.441e-02  1.540e+03   0.381 0.703581
## ---
## Signif. codes:  0 '***' 0.001 '**' 0.01 '*' 0.05 '.' 0.1 ' ' 1
##
## Correlation of Fixed Effects:
##             (Intr) Lbr_10 BMI    HEI    FIsqrt Age
## Lbrry_szl10 -0.976
## BMI          0.000 -0.001
## HEI          0.014 -0.010  0.101
## FIsqrt       0.001  0.004 -0.284  0.073
## Age         -0.042  0.042  0.036  0.023 -0.317
## zyg         -0.062  0.059 -0.033 -0.029 -0.016 -0.083
##
## $Simpson[[2]]
##                       stdcoef      stdse
## (Intercept)        0.000000000 0.00000000
## Library_sizelog10 -0.008794709 0.02476094
## BMI               -0.064246926 0.02546545
## HEI                0.055085757 0.02445755
## FIsqrt            -0.114402237 0.02946273
## Age                0.075009789 0.02613962
## zyg                0.009288892 0.02440840
```

Crude Models

Education

```r
alphaaovsEDUF <- apply(alphas, 2, function(x) {
    mod <- lmer(x ~ eduff + Library_sizelog10 + zyg + (1 | SequencingRunID) +
        (1 | ExtractionPlateLoadedby) + (1 | Extractedby) + (1 |
        CollectionMethod), REML = F, data = mapalph)
    beta <- stdCoef.merMod(mod)
    sum <- summary(mod)
    list <- list(sum, beta)
    return(list)
})

alphaaovsEDUF
```

```
## $Chao1
## $Chao1[[1]]
## Linear mixed model fit by maximum likelihood . t-tests use
##   Satterthwaite's method [lmerModLmerTest]
## Formula: x ~ eduff + Library_sizelog10 + zyg + (1 | SequencingRunID) +
##     (1 | ExtractionPlateLoadedby) + (1 | Extractedby) + (1 |
```

```
##     CollectionMethod)
##    Data: mapalph
##
##      AIC      BIC   logLik deviance df.resid
##   2873.9   2931.7  -1425.9   2851.9     1415
##
## Scaled residuals:
##     Min      1Q  Median      3Q     Max
## -3.0276 -0.5606 -0.0996  0.3980  7.4747
##
## Random effects:
##  Groups                 Name        Variance  Std.Dev.
##  SequencingRunID        (Intercept) 2.312e-01 4.808e-01
##  ExtractionPlateLoadedby (Intercept) 3.028e-02 1.740e-01
##  Extractedby            (Intercept) 1.564e-15 3.954e-08
##  CollectionMethod       (Intercept) 0.000e+00 0.000e+00
##  Residual                           4.008e-01 6.331e-01
## Number of obs: 1426, groups:
## SequencingRunID, 33; ExtractionPlateLoadedby, 10; Extractedby, 2; CollectionMethod, 2
##
## Fixed effects:
##                     Estimate Std. Error         df t value Pr(>|t|)
## (Intercept)       -1.371e+01  5.376e-01  1.008e+03 -25.509   <2e-16 ***
## eduff2             4.372e-03  5.391e-02  1.396e+03   0.081    0.935
## eduff3            -1.035e-02  5.028e-02  1.395e+03  -0.206    0.837
## eduff4             2.739e-02  5.348e-02  1.393e+03   0.512    0.609
## Library_sizelog10  2.786e+00  1.075e-01  1.329e+03  25.923   <2e-16 ***
## zyg               -1.224e-02  1.768e-02  1.402e+03  -0.692    0.489
## ---
## Signif. codes:  0 '***' 0.001 '**' 0.01 '*' 0.05 '.' 0.1 ' ' 1
##
## Correlation of Fixed Effects:
##             (Intr) eduff2 eduff3 eduff4 Lbr_10
## eduff2      -0.053
## eduff3      -0.043  0.616
## eduff4      -0.021  0.580  0.628
## Lbrry_szl10 -0.977 -0.003 -0.020 -0.041
## zyg         -0.027  0.008  0.051  0.085  0.023
##
## $Chao1[[2]]
##                        stdcoef      stdse
## (Intercept)        0.000000000 0.00000000
## eduff2             0.001839026 0.02267690
## eduff3            -0.004862308 0.02362287
## eduff4             0.011782500 0.02300679
## Library_sizelog10  0.523411724 0.02019084
## zyg               -0.011995119 0.01733118
##
##
## $Shannon
## $Shannon[[1]]
## Linear mixed model fit by maximum likelihood . t-tests use
##   Satterthwaite's method [lmerModLmerTest]
## Formula: x ~ eduff + Library_sizelog10 + zyg + (1 | SequencingRunID) +
```

```
##     (1 | ExtractionPlateLoadedby) + (1 | Extractedby) + (1 |
##     CollectionMethod)
##    Data: mapalph
##
##      AIC      BIC   logLik deviance df.resid
##   4086.5   4144.4  -2032.3   4064.5     1415
##
## Scaled residuals:
##     Min      1Q  Median      3Q     Max
## -5.2129 -0.5409  0.1168  0.7137  2.1513
##
## Random effects:
##  Groups                  Name        Variance Std.Dev.
##  SequencingRunID         (Intercept) 0.01159  0.1076
##  ExtractionPlateLoadedby (Intercept) 0.00000  0.0000
##  Extractedby             (Intercept) 0.00000  0.0000
##  CollectionMethod        (Intercept) 0.00000  0.0000
##  Residual                            1.00359  1.0018
## Number of obs: 1426, groups:
## SequencingRunID, 33; ExtractionPlateLoadedby, 10; Extractedby, 2; CollectionMethod, 2
##
## Fixed effects:
##                    Estimate Std. Error         df t value Pr(>|t|)
## (Intercept)         0.53295    0.73091  325.93589   0.729    0.466
## eduff2              0.08756    0.08444 1416.41992   1.037    0.300
## eduff3              0.09292    0.07888 1418.72704   1.178    0.239
## eduff4              0.12440    0.08376 1416.86916   1.485    0.138
## Library_sizelog10  -0.12619    0.14912  307.19493  -0.846    0.398
## zyg                 0.01275    0.02730 1399.28566   0.467    0.640
##
## Correlation of Fixed Effects:
##             (Intr) eduff2 eduff3 eduff4 Lbr_10
## eduff2      -0.079
## eduff3      -0.062  0.619
## eduff4      -0.022  0.582  0.629
## Lbrry_szl10 -0.996  0.012 -0.010 -0.046
## zyg         -0.060  0.010  0.052  0.092  0.055
##
## $Shannon[[2]]
##                       stdcoef      stdse
## (Intercept)        0.00000000 0.00000000
## eduff2             0.03687380 0.03555921
## eduff3             0.04370607 0.03709924
## eduff4             0.05357895 0.03607500
## Library_sizelog10 -0.02373280 0.02804467
## zyg                0.01251310 0.02678320
##
##
## $Simpson
## $Simpson[[1]]
## Linear mixed model fit by maximum likelihood . t-tests use
##   Satterthwaite's method [lmerModLmerTest]
## Formula: x ~ eduff + Library_sizelog10 + zyg + (1 | SequencingRunID) +
##     (1 | ExtractionPlateLoadedby) + (1 | Extractedby) + (1 |
```

```
##     CollectionMethod)
##    Data: mapalph
##
##      AIC      BIC   logLik deviance df.resid
##   4128.1   4185.9  -2053.0   4106.1     1415
##
## Scaled residuals:
##      Min       1Q   Median       3Q      Max
## -10.2973  -0.1671   0.2843   0.5369   0.9862
##
## Random effects:
##  Groups                  Name        Variance Std.Dev.
##  SequencingRunID         (Intercept) 0.005841 0.07643
##  ExtractionPlateLoadedby (Intercept) 0.000000 0.00000
##  Extractedby             (Intercept) 0.000000 0.00000
##  CollectionMethod        (Intercept) 0.000000 0.00000
##  Residual                            1.037375 1.01852
## Number of obs: 1426, groups:
## SequencingRunID, 33; ExtractionPlateLoadedby, 10; Extractedby, 2; CollectionMethod, 2
##
## Fixed effects:
##                    Estimate Std. Error         df t value Pr(>|t|)
## (Intercept)         0.49663    0.72655  200.94934   0.684    0.495
## eduff2              0.07137    0.08574 1418.51629   0.832    0.405
## eduff3              0.10043    0.08008 1422.57002   1.254    0.210
## eduff4              0.06599    0.08505 1420.62834   0.776    0.438
## Library_sizelog10  -0.11568    0.14812  188.97679  -0.781    0.436
## zyg                -0.00170    0.02765 1362.17650  -0.061    0.951
##
## Correlation of Fixed Effects:
##             (Intr) eduff2 eduff3 eduff4 Lbr_10
## eduff2      -0.084
## eduff3      -0.066  0.619
## eduff4      -0.024  0.583  0.629
## Lbrry_szl10 -0.996  0.016 -0.008 -0.045
## zyg         -0.062  0.011  0.052  0.093  0.057
##
## $Simpson[[2]]
##                        stdcoef      stdse
## (Intercept)        0.000000000 0.00000000
## eduff2             0.029639902 0.03560787
## eduff3             0.046580997 0.03714413
## eduff4             0.028027246 0.03612226
## Library_sizelog10 -0.021453470 0.02747010
## zyg               -0.001644501 0.02675475
```

Income

```
alphaaovsIncome <- apply(alphas, 2, function(x) {
    mod <- lmer(x ~ Income4F + Library_sizelog10 + zyg + (1 |
        SequencingRunID) + (1 | ExtractionPlateLoadedby) + (1 |
        Extractedby) + (1 | CollectionMethod), REML = F, data = mapalph)
```

```r
    beta <- stdCoef.merMod(mod)
    sum <- summary(mod)
    list <- list(sum, beta)
    return(list)
})

alphaaovsIncome
```

```
## $Chao1
## $Chao1[[1]]
## Linear mixed model fit by maximum likelihood . t-tests use
##   Satterthwaite's method [lmerModLmerTest]
## Formula: x ~ Income4F + Library_sizelog10 + zyg + (1 | SequencingRunID) +
##     (1 | ExtractionPlateLoadedby) + (1 | Extractedby) + (1 |
##     CollectionMethod)
##    Data: mapalph
##
##      AIC      BIC   logLik deviance df.resid
##   1505.0   1556.5   -741.5   1483.0      788
##
## Scaled residuals:
##     Min      1Q  Median      3Q     Max
## -2.7262 -0.5704 -0.0786  0.4218  7.5898
##
## Random effects:
##  Groups                  Name        Variance Std.Dev.
##  SequencingRunID         (Intercept) 0.17734  0.4211
##  ExtractionPlateLoadedby (Intercept) 0.04372  0.2091
##  Extractedby             (Intercept) 0.00000  0.0000
##  CollectionMethod        (Intercept) 0.00000  0.0000
##  Residual                            0.33466  0.5785
## Number of obs: 799, groups:
## SequencingRunID, 33; ExtractionPlateLoadedby, 9; Extractedby, 2; CollectionMethod, 2
##
## Fixed effects:
##                     Estimate Std. Error        df t value Pr(>|t|)
## (Intercept)        -13.46641    0.69840 634.02936 -19.282   <2e-16 ***
## Income4F2            0.04913    0.06582 771.33380   0.746   0.4556
## Income4F3            0.13413    0.06006 767.35132   2.233   0.0258 *
## Income4F4            0.12788    0.06988 771.05525   1.830   0.0676 .
## Library_sizelog10    2.72230    0.14002 746.97549  19.442   <2e-16 ***
## zyg                 -0.02815    0.02133 777.04403  -1.320   0.1873
## ---
## Signif. codes:  0 '***' 0.001 '**' 0.01 '*' 0.05 '.' 0.1 ' ' 1
##
## Correlation of Fixed Effects:
##              (Intr) Inc4F2 Inc4F3 Inc4F4 Lbr_10
## Income4F2   -0.092
## Income4F3   -0.119  0.643
## Income4F4   -0.078  0.556  0.598
## Lbrry_szl10 -0.985  0.036  0.059  0.026
## zyg         -0.042 -0.023  0.003  0.000  0.042
##
## $Chao1[[2]]
```

```
##                        stdcoef      stdse
## (Intercept)        0.00000000 0.00000000
## Income4F2          0.02345129 0.03141836
## Income4F3          0.07166473 0.03209221
## Income4F4          0.05432982 0.02968845
## Library_sizelog10  0.54080953 0.02781645
## zyg               -0.03096442 0.02346278
##
##
## $Shannon
## $Shannon[[1]]
## Linear mixed model fit by maximum likelihood . t-tests use
##   Satterthwaite's method [lmerModLmerTest]
## Formula: x ~ Income4F + Library_sizelog10 + zyg + (1 | SequencingRunID) +
##     (1 | ExtractionPlateLoadedby) + (1 | Extractedby) + (1 |
##     CollectionMethod)
##    Data: mapalph
##
##      AIC      BIC   logLik deviance df.resid
##   2319.7   2371.2  -1148.9   2297.7      788
##
## Scaled residuals:
##     Min      1Q  Median      3Q     Max
## -4.9629 -0.5385  0.1435  0.6913  2.1157
##
## Random effects:
##  Groups                  Name        Variance   Std.Dev.
##  SequencingRunID         (Intercept)  8.737e-03 9.347e-02
##  ExtractionPlateLoadedby (Intercept)  7.623e-03 8.731e-02
##  Extractedby             (Intercept)  2.456e-15 4.956e-08
##  CollectionMethod        (Intercept) 5.265e-113 7.256e-57
##  Residual                             1.026e+00 1.013e+00
## Number of obs: 799, groups:
## SequencingRunID, 33; ExtractionPlateLoadedby, 9; Extractedby, 2; CollectionMethod, 2
##
## Fixed effects:
##                   Estimate Std. Error        df t value Pr(>|t|)
## (Intercept)        0.27905    1.02301 236.16280   0.273  0.78526
## Income4F2          0.07316    0.11254 795.93035   0.650  0.51581
## Income4F3          0.20888    0.10398 786.18715   2.009  0.04489 *
## Income4F4          0.35918    0.12042 794.18918   2.983  0.00295 **
## Library_sizelog10  -0.08168    0.20746 231.03242  -0.394  0.69417
## zyg                0.02439    0.03613 772.63267   0.675  0.49978
## ---
## Signif. codes:  0 '***' 0.001 '**' 0.01 '*' 0.05 '.' 0.1 ' ' 1
##
## Correlation of Fixed Effects:
##             (Intr) Inc4F2 Inc4F3 Inc4F4 Lbr_10
## Income4F2   -0.086
## Income4F3   -0.133  0.640
## Income4F4   -0.086  0.553  0.597
## Lbrry_szl10 -0.996  0.021  0.063  0.026
## zyg         -0.056 -0.040 -0.004 -0.013  0.055
##
```

```
## $Shannon[[2]]
##                      stdcoef      stdse
## (Intercept)        0.00000000 0.00000000
## Income4F2          0.03101407 0.04770658
## Income4F3          0.09910957 0.04933553
## Income4F4          0.13551225 0.04543419
## Library_sizelog10 -0.01440921 0.03660017
## zyg                0.02382943 0.03529517
##
##
## $Simpson
## $Simpson[[1]]
## Linear mixed model fit by maximum likelihood . t-tests use
##   Satterthwaite's method [lmerModLmerTest]
## Formula: x ~ Income4F + Library_sizelog10 + zyg + (1 | SequencingRunID) +
##     (1 | ExtractionPlateLoadedby) + (1 | Extractedby) + (1 |
##     CollectionMethod)
##    Data: mapalph
##
##      AIC      BIC   logLik deviance df.resid
##   2401.0   2452.5  -1189.5   2379.0      788
##
## Scaled residuals:
##     Min      1Q  Median      3Q     Max
## -9.5921 -0.1723  0.2695  0.5016  1.0549
##
## Random effects:
##  Groups                  Name        Variance Std.Dev.
##  SequencingRunID         (Intercept) 0.01056  0.1028
##  ExtractionPlateLoadedby (Intercept) 0.00000  0.0000
##  Extractedby             (Intercept) 0.02692  0.1641
##  CollectionMethod        (Intercept) 0.00000  0.0000
##  Residual                            1.13575  1.0657
## Number of obs: 799, groups:
## SequencingRunID, 33; ExtractionPlateLoadedby, 9; Extractedby, 2; CollectionMethod, 2
##
## Fixed effects:
##                    Estimate Std. Error        df t value Pr(>|t|)
## (Intercept)         0.25452    1.07204 230.26089   0.237  0.81255
## Income4F2           0.14349    0.11819 789.68418   1.214  0.22507
## Income4F3           0.27968    0.10933 787.25185   2.558  0.01071 *
## Income4F4           0.39206    0.12666 794.77416   3.095  0.00203 **
## Library_sizelog10  -0.07259    0.21664 229.19388  -0.335  0.73788
## zyg                 0.01021    0.03800 774.66383   0.269  0.78831
## ---
## Signif. codes:  0 '***' 0.001 '**' 0.01 '*' 0.05 '.' 0.1 ' ' 1
##
## Correlation of Fixed Effects:
##             (Intr) Inc4F2 Inc4F3 Inc4F4 Lbr_10
## Income4F2   -0.089
## Income4F3   -0.134  0.640
## Income4F4   -0.089  0.553  0.597
## Lbrry_szl10 -0.989  0.025  0.066  0.031
## zyg         -0.061 -0.039 -0.004 -0.013  0.059
```

```
##
## $Simpson[[2]]
##                        stdcoef      stdse
## (Intercept)        0.000000000 0.00000000
## Income4F2          0.057838604 0.04763899
## Income4F3          0.126186794 0.04933019
## Income4F4          0.140657565 0.04544141
## Library_sizelog10 -0.012177411 0.03634351
## zyg                0.009481745 0.03530102
```

IMD

```
alphaaovsIMDF <- apply(alphas, 2, function(x) {
    mod <- lmer(x ~ IMD5f + Library_sizelog10 + zyg + (1 | SequencingRunID) +
        (1 | ExtractionPlateLoadedby) + (1 | Extractedby) + (1 |
        CollectionMethod), REML = F, data = mapalph)
    beta <- stdCoef.merMod(mod)
    sum <- summary(mod)
    list <- list(sum, beta)
    return(list)
})

alphaaovsIMDF
```

```
## $Chao1
## $Chao1[[1]]
## Linear mixed model fit by maximum likelihood . t-tests use
##   Satterthwaite's method [lmerModLmerTest]
## Formula: x ~ IMD5f + Library_sizelog10 + zyg + (1 | SequencingRunID) +
##     (1 | ExtractionPlateLoadedby) + (1 | Extractedby) + (1 |
##     CollectionMethod)
##    Data: mapalph
##
##      AIC      BIC   logLik deviance df.resid
##   3323.9   3389.0  -1650.0   3299.9     1660
##
## Scaled residuals:
##     Min      1Q  Median      3Q     Max
## -3.4375 -0.5547 -0.1055  0.3918  7.5223
##
## Random effects:
##  Groups                  Name        Variance Std.Dev.
##  SequencingRunID         (Intercept) 0.206454 0.45437
##  ExtractionPlateLoadedby (Intercept) 0.034945 0.18694
##  Extractedby             (Intercept) 0.000000 0.00000
##  CollectionMethod        (Intercept) 0.001316 0.03628
##  Residual                            0.393305 0.62714
## Number of obs: 1672, groups:
## SequencingRunID, 33; ExtractionPlateLoadedby, 10; Extractedby, 2; CollectionMethod, 2
##
## Fixed effects:
##                      Estimate Std. Error         df t value Pr(>|t|)
## (Intercept)         -13.54543    0.49433 1157.99143 -27.402   <2e-16 ***
```

```
## IMD5f2              -0.01305    0.04909 1639.55537  -0.266    0.790
## IMD5f3               0.05561    0.04891 1637.27818   1.137    0.256
## IMD5f4               0.05221    0.04909 1641.68848   1.064    0.288
## IMD5f5               0.03843    0.04903 1639.18855   0.784    0.433
## Library_sizelog10    2.74568    0.09830 1601.32849  27.930   <2e-16 ***
## zyg                 -0.01785    0.01592 1649.01910  -1.121    0.262
## ---
## Signif. codes:  0 '***' 0.001 '**' 0.01 '*' 0.05 '.' 0.1 ' ' 1
##
## Correlation of Fixed Effects:
##             (Intr) IMD5f2 IMD5f3 IMD5f4 IMD5f5 Lbr_10
## IMD5f2      -0.036
## IMD5f3      -0.026  0.494
## IMD5f4      -0.042  0.500  0.492
## IMD5f5      -0.027  0.501  0.498  0.504
## Lbrry_szl10 -0.973 -0.017 -0.024 -0.009 -0.021
## zyg         -0.027 -0.004 -0.020  0.006 -0.018  0.028
##
## $Chao1[[2]]
##                        stdcoef      stdse
## (Intercept)        0.000000000 0.00000000
## IMD5f2            -0.005213515 0.01961158
## IMD5f3             0.022238865 0.01955918
## IMD5f4             0.020881767 0.01963227
## IMD5f5             0.015385347 0.01963162
## Library_sizelog10  0.516319257 0.01848602
## zyg               -0.017846224 0.01591694
##
##
## $Shannon
## $Shannon[[1]]
## Linear mixed model fit by maximum likelihood . t-tests use
##   Satterthwaite's method [lmerModLmerTest]
## Formula: x ~ IMD5f + Library_sizelog10 + zyg + (1 | SequencingRunID) +
##     (1 | ExtractionPlateLoadedby) + (1 | Extractedby) + (1 |
##     CollectionMethod)
##    Data: mapalph
##
##      AIC      BIC   logLik deviance df.resid
##   4751.0   4816.0  -2363.5   4727.0     1660
##
## Scaled residuals:
##     Min      1Q  Median      3Q     Max
## -5.2548 -0.5413  0.1324  0.6995  2.1071
##
## Random effects:
##  Groups                  Name        Variance  Std.Dev.
##  SequencingRunID         (Intercept) 1.729e-02 1.315e-01
##  ExtractionPlateLoadedby (Intercept) 1.503e-15 3.877e-08
##  Extractedby             (Intercept) 0.000e+00 0.000e+00
##  CollectionMethod        (Intercept) 0.000e+00 0.000e+00
##  Residual                            9.777e-01 9.888e-01
## Number of obs: 1672, groups:
## SequencingRunID, 33; ExtractionPlateLoadedby, 10; Extractedby, 2; CollectionMethod, 2
```

```
##
## Fixed effects:
##                    Estimate Std. Error         df t value Pr(>|t|)
## (Intercept)         0.23093    0.68137  567.49504   0.339  0.73480
## IMD5f2              0.24011    0.07684 1661.41055   3.125  0.00181 **
## IMD5f3              0.10071    0.07670 1657.35437   1.313  0.18935
## IMD5f4              0.13188    0.07675 1661.45823   1.718  0.08593 .
## IMD5f5              0.16540    0.07676 1663.73515   2.155  0.03132 *
## Library_sizelog10  -0.07637    0.13937  555.00650  -0.548  0.58394
## zyg                 0.02185    0.02456 1655.43399   0.889  0.37386
## ---
## Signif. codes:  0 '***' 0.001 '**' 0.01 '*' 0.05 '.' 0.1 ' ' 1
##
## Correlation of Fixed Effects:
##             (Intr) IMD5f2 IMD5f3 IMD5f4 IMD5f5 Lbr_10
## IMD5f2      -0.030
## IMD5f3      -0.035  0.497
## IMD5f4      -0.040  0.500  0.497
## IMD5f5      -0.034  0.501  0.499  0.502
## Lbrry_szl10 -0.996 -0.026 -0.021 -0.016 -0.022
## zyg         -0.061 -0.004 -0.021  0.004 -0.021  0.061
##
## $Shannon[[2]]
##                       stdcoef      stdse
## (Intercept)        0.00000000 0.00000000
## IMD5f2             0.09592243 0.03069725
## IMD5f3             0.04027683 0.03067433
## IMD5f4             0.05274214 0.03069414
## IMD5f5             0.06622269 0.03073310
## Library_sizelog10 -0.01436157 0.02620913
## zyg                0.02184716 0.02456120
##
##
## $Simpson
## $Simpson[[1]]
## Linear mixed model fit by maximum likelihood . t-tests use
##   Satterthwaite's method [lmerModLmerTest]
## Formula: x ~ IMD5f + Library_sizelog10 + zyg + (1 | SequencingRunID) +
##     (1 | ExtractionPlateLoadedby) + (1 | Extractedby) + (1 |
##     CollectionMethod)
##    Data: mapalph
##
##      AIC      BIC   logLik deviance df.resid
##   4761.2   4826.2  -2368.6   4737.2     1660
##
## Scaled residuals:
##      Min       1Q   Median       3Q      Max
## -10.5572  -0.1908   0.2796   0.5440   0.9891
##
## Random effects:
##  Groups                  Name        Variance Std.Dev.
##  SequencingRunID         (Intercept) 0.006719 0.08197
##  ExtractionPlateLoadedby (Intercept) 0.000000 0.00000
##  Extractedby             (Intercept) 0.015240 0.12345
```

```
##  CollectionMethod        (Intercept) 0.000000 0.00000
##  Residual                            0.987680 0.99382
## Number of obs: 1672, groups:
## SequencingRunID, 33; ExtractionPlateLoadedby, 10; Extractedby, 2; CollectionMethod, 2
##
## Fixed effects:
##                     Estimate Std. Error         df t value Pr(>|t|)
## (Intercept)        2.861e-01  6.677e-01  2.267e+02   0.428   0.6687
## IMD5f2             1.691e-01  7.708e-02  1.666e+03   2.193   0.0284 *
## IMD5f3             8.773e-02  7.697e-02  1.659e+03   1.140   0.2546
## IMD5f4             1.166e-01  7.699e-02  1.666e+03   1.515   0.1301
## IMD5f5             1.054e-01  7.699e-02  1.668e+03   1.369   0.1712
## Library_sizelog10 -5.955e-02  1.354e-01  2.392e+02  -0.440   0.6605
## zyg                8.918e-03  2.456e-02  1.599e+03   0.363   0.7166
## ---
## Signif. codes:  0 '***' 0.001 '**' 0.01 '*' 0.05 '.' 0.1 ' ' 1
##
## Correlation of Fixed Effects:
##              (Intr) IMD5f2 IMD5f3 IMD5f4 IMD5f5 Lbr_10
## IMD5f2      -0.030
## IMD5f3      -0.038  0.498
## IMD5f4      -0.042  0.499  0.498
## IMD5f5      -0.034  0.500  0.499  0.501
## Lbrry_szl10 -0.985 -0.028 -0.020 -0.016 -0.023
## zyg         -0.067 -0.005 -0.021  0.003 -0.022  0.066
##
## $Simpson[[2]]
##                       stdcoef      stdse
## (Intercept)        0.000000000 0.00000000
## IMD5f2             0.067533858 0.03079377
## IMD5f3             0.035085128 0.03078467
## IMD5f4             0.046636343 0.03079154
## IMD5f5             0.042200307 0.03082670
## Library_sizelog10 -0.011198889 0.02546711
## zyg                0.008917762 0.02456010
```

Adjusted Models

Education

```
alphaaovsEducfull <- apply(alphas, 2, function(x) {
    mod <- lmer(x ~ eduff + Library_sizelog10 + zyg + BMI + HEI +
        FIsqrt + Age + (1 | HEIv) + (1 | Frailty_Index_Vers) +
        (1 | SequencingRunID) + (1 | ExtractionPlateLoadedby) +
        (1 | Extractedby) + (1 | CollectionMethod), REML = F,
        data = mapalph)
    beta <- stdCoef.merMod(mod)
    sum <- summary(mod)
    list <- list(sum, beta)
    return(list)
})
```

```
alphaaovsEducfull
```

```
## $Chao1
## $Chao1[[1]]
## Linear mixed model fit by maximum likelihood . t-tests use
##   Satterthwaite's method [lmerModLmerTest]
## Formula: x ~ eduff + Library_sizelog10 + zyg + BMI + HEI + FIsqrt + Age +
##     (1 | HEIv) + (1 | Frailty_Index_Vers) + (1 | SequencingRunID) +
##     (1 | ExtractionPlateLoadedby) + (1 | Extractedby) + (1 |
##     CollectionMethod)
##    Data: mapalph
##
##      AIC      BIC   logLik deviance df.resid
##   2883.3   2972.8  -1424.7   2849.3     1409
##
## Scaled residuals:
##     Min      1Q  Median      3Q     Max
## -3.0019 -0.5634 -0.0987  0.4107  7.4701
##
## Random effects:
##  Groups                  Name        Variance  Std.Dev.
##  SequencingRunID         (Intercept) 2.300e-01 4.796e-01
##  ExtractionPlateLoadedby (Intercept) 3.042e-02 1.744e-01
##  CollectionMethod        (Intercept) 0.000e+00 0.000e+00
##  Extractedby             (Intercept) 9.378e-16 3.062e-08
##  Frailty_Index_Vers      (Intercept) 0.000e+00 0.000e+00
##  HEIv                    (Intercept) 0.000e+00 0.000e+00
##  Residual                            4.001e-01 6.326e-01
## Number of obs: 1426, groups:
## SequencingRunID, 33; ExtractionPlateLoadedby, 10; CollectionMethod, 2; Extractedby, 2; Frailty_Index_Ve
##
## Fixed effects:
##                     Estimate Std. Error         df t value Pr(>|t|)
## (Intercept)       -1.371e+01  5.401e-01  1.002e+03 -25.383   <2e-16 ***
## eduff2             7.345e-04  5.520e-02  1.396e+03   0.013    0.989
## eduff3            -1.452e-02  5.218e-02  1.395e+03  -0.278    0.781
## eduff4             2.400e-02  5.798e-02  1.396e+03   0.414    0.679
## Library_sizelog10  2.786e+00  1.078e-01  1.329e+03  25.850   <2e-16 ***
## zyg               -1.173e-02  1.773e-02  1.402e+03  -0.662    0.508
## BMI                7.404e-03  1.835e-02  1.395e+03   0.404    0.687
## HEI               -1.966e-02  1.731e-02  1.395e+03  -1.136    0.256
## FIsqrt            -2.073e-02  1.863e-02  1.399e+03  -1.113    0.266
## Age               -1.551e-03  1.867e-02  1.404e+03  -0.083    0.934
## ---
## Signif. codes:  0 '***' 0.001 '**' 0.01 '*' 0.05 '.' 0.1 ' ' 1
##
## Correlation of Fixed Effects:
##             (Intr) eduff2 eduff3 eduff4 Lbr_10 zyg    BMI    HEI    FIsqrt
## eduff2      -0.073
## eduff3      -0.068  0.638
## eduff4      -0.057  0.606  0.662
## Lbrry_szl10 -0.977  0.013  0.001 -0.008
## zyg         -0.020 -0.008  0.030  0.049  0.018
```

```
## BMI          0.003  0.052  0.066  0.115 -0.008 -0.036
## HEI          0.025 -0.024 -0.024 -0.041 -0.021 -0.027  0.088
## FIsqrt      -0.009  0.055  0.060  0.040 -0.003  0.012 -0.235  0.066
## Age         -0.095  0.179  0.226  0.337  0.082 -0.073  0.004  0.017 -0.196
##
## $Chao1[[2]]
##                        stdcoef      stdse
## (Intercept)        0.0000000000 0.00000000
## eduff2             0.0003089969 0.02322184
## eduff3            -0.0068207803 0.02451732
## eduff4             0.0103261950 0.02494619
## Library_sizelog10  0.5234396003 0.02024910
## zyg               -0.0114999263 0.01737898
## BMI                0.0071632178 0.01775178
## HEI               -0.0193594859 0.01704248
## FIsqrt            -0.0201401630 0.01809142
## Age               -0.0015655972 0.01884896
##
##
## $Shannon
## $Shannon[[1]]
## Linear mixed model fit by maximum likelihood . t-tests use
##   Satterthwaite's method [lmerModLmerTest]
## Formula: x ~ eduff + Library_sizelog10 + zyg + BMI + HEI + FIsqrt + Age +
##     (1 | HEIv) + (1 | Frailty_Index_Vers) + (1 | SequencingRunID) +
##     (1 | ExtractionPlateLoadedby) + (1 | Extractedby) + (1 |
##     CollectionMethod)
##    Data: mapalph
##
##      AIC      BIC   logLik deviance df.resid
##   4030.6   4120.0  -1998.3   3996.6     1409
##
## Scaled residuals:
##     Min      1Q  Median      3Q     Max
## -5.6168 -0.5032  0.1138  0.6959  2.2885
##
## Random effects:
##  Groups                  Name        Variance  Std.Dev.
##  SequencingRunID         (Intercept) 5.714e-03 7.559e-02
##  ExtractionPlateLoadedby (Intercept) 4.064e-17 6.375e-09
##  CollectionMethod        (Intercept) 0.000e+00 0.000e+00
##  Extractedby             (Intercept) 9.691e-03 9.844e-02
##  Frailty_Index_Vers      (Intercept) 1.717e-02 1.310e-01
##  HEIv                    (Intercept) 0.000e+00 0.000e+00
##  Residual                            9.565e-01 9.780e-01
## Number of obs: 1426, groups:
## SequencingRunID, 33; ExtractionPlateLoadedby, 10; CollectionMethod, 2; Extractedby, 2; Frailty_Index_Ve
##
## Fixed effects:
##                     Estimate Std. Error        df t value Pr(>|t|)
## (Intercept)        4.704e-01  7.110e-01  2.088e+02   0.662  0.50899
## eduff2             6.595e-02  8.437e-02  1.421e+03   0.782  0.43454
## eduff3             6.589e-02  7.976e-02  1.424e+03   0.826  0.40889
## eduff4             9.586e-02  8.855e-02  1.424e+03   1.083  0.27920
```

```
## Library_sizelog10 -1.035e-01  1.430e-01  2.136e+02  -0.724  0.47005
## zyg                8.811e-03  2.666e-02  1.365e+03   0.331  0.74103
## BMI               -7.557e-02  2.817e-02  1.422e+03  -2.683  0.00738 **
## HEI                8.559e-02  2.649e-02  1.424e+03   3.232  0.00126 **
## FIsqrt            -1.832e-01  3.211e-02  7.668e+02  -5.706 1.65e-08 ***
## Age                1.263e-01  2.920e-02  1.246e+03   4.325 1.64e-05 ***
## ---
## Signif. codes:  0 '***' 0.001 '**' 0.01 '*' 0.05 '.' 0.1 ' ' 1
##
## Correlation of Fixed Effects:
##             (Intr) eduff2 eduff3 eduff4 Lbr_10 zyg    BMI    HEI    FIsqrt
## eduff2      -0.098
## eduff3      -0.083  0.640
## eduff4      -0.054  0.607  0.661
## Lbrry_szl10 -0.980  0.028  0.009 -0.017
## zyg         -0.056 -0.003  0.033  0.059  0.051
## BMI         -0.003  0.050  0.061  0.103 -0.004 -0.033
## HEI          0.012 -0.027 -0.025 -0.042 -0.006 -0.033  0.081
## FIsqrt      -0.008  0.054  0.070  0.063  0.008 -0.005 -0.264  0.076
## Age         -0.075  0.168  0.200  0.305  0.052 -0.053  0.038  0.003 -0.290
##
## $Shannon[[2]]
##                        stdcoef      stdse
## (Intercept)        0.000000000 0.00000000
## eduff2             0.027774189 0.03553232
## eduff3             0.030991309 0.03751521
## eduff4             0.041284681 0.03813697
## Library_sizelog10 -0.019456500 0.02688504
## zyg                0.008644742 0.02615168
## BMI               -0.073199457 0.02728256
## HEI                0.084380348 0.02611127
## FIsqrt            -0.178129404 0.03121884
## Age                0.127637130 0.02950929
##
##
## $Simpson
## $Simpson[[1]]
## Linear mixed model fit by maximum likelihood . t-tests use
##   Satterthwaite's method [lmerModLmerTest]
## Formula: x ~ eduff + Library_sizelog10 + zyg + BMI + HEI + FIsqrt + Age +
##     (1 | HEIv) + (1 | Frailty_Index_Vers) + (1 | SequencingRunID) +
##     (1 | ExtractionPlateLoadedby) + (1 | Extractedby) + (1 |
##     CollectionMethod)
##    Data: mapalph
##
##      AIC      BIC   logLik deviance df.resid
##   4115.2   4204.7  -2040.6   4081.2     1409
##
## Scaled residuals:
##      Min       1Q   Median       3Q      Max
## -10.5752  -0.1705   0.2632   0.5263   1.1373
##
## Random effects:
##  Groups                  Name        Variance  Std.Dev.
```

```
##  SequencingRunID         (Intercept) 0.0001649 0.01284
##  ExtractionPlateLoadedby (Intercept) 0.0000000 0.00000
##  CollectionMethod        (Intercept) 0.0000000 0.00000
##  Extractedby             (Intercept) 0.0229731 0.15157
##  Frailty_Index_Vers      (Intercept) 0.0092494 0.09617
##  HEIv                    (Intercept) 0.0000000 0.00000
##  Residual                            1.0197318 1.00982
## Number of obs: 1426, groups:
## SequencingRunID, 33; ExtractionPlateLoadedby, 10; CollectionMethod, 2; Extractedby, 2; Frailty_Index_Ve
##
## Fixed effects:
##                    Estimate Std. Error        df t value Pr(>|t|)
## (Intercept)        4.682e-01  7.111e-01  1.408e+02   0.658 0.511368
## eduff2             5.674e-02  8.695e-02  1.423e+03   0.653 0.514127
## eduff3             8.118e-02  8.213e-02  1.419e+03   0.988 0.323090
## eduff4             4.420e-02  9.115e-02  1.416e+03   0.485 0.627789
## Library_sizelog10 -8.819e-02  1.425e-01  1.389e+02  -0.619 0.537063
## zyg               -5.819e-03  2.738e-02  1.298e+03  -0.213 0.831707
## BMI               -3.690e-02  2.897e-02  1.396e+03  -1.274 0.202957
## HEI                5.982e-02  2.729e-02  1.423e+03   2.192 0.028547 *
## FIsqrt            -1.184e-01  3.261e-02  3.313e+02  -3.632 0.000326 ***
## Age                7.276e-02  2.984e-02  9.768e+02   2.438 0.014936 *
## ---
## Signif. codes:  0 '***' 0.001 '**' 0.01 '*' 0.05 '.' 0.1 ' ' 1
##
## Correlation of Fixed Effects:
##             (Intr) eduff2 eduff3 eduff4 Lbr_10 zyg    BMI    HEI    FIsqrt
## eduff2      -0.104
## eduff3      -0.087  0.640
## eduff4      -0.053  0.607  0.661
## Lbrry_szl10 -0.977  0.033  0.011 -0.020
## zyg         -0.059 -0.003  0.032  0.061  0.054
## BMI         -0.007  0.050  0.062  0.103  0.000 -0.033
## HEI          0.012 -0.027 -0.025 -0.044 -0.005 -0.033  0.081
## FIsqrt      -0.004  0.054  0.070  0.060  0.003 -0.004 -0.264  0.077
## Age         -0.072  0.168  0.199  0.307  0.050 -0.053  0.038  0.004 -0.278
##
## $Simpson[[2]]
##                       stdcoef      stdse
## (Intercept)        0.000000000 0.00000000
## eduff2             0.023564305 0.03610894
## eduff3             0.037655272 0.03809438
## eduff4             0.018773705 0.03871293
## Library_sizelog10 -0.016355278 0.02643072
## zyg               -0.005629978 0.02648712
## BMI               -0.035244500 0.02766946
## HEI                0.058153186 0.02653070
## FIsqrt            -0.113573448 0.03127128
## Age                0.072523730 0.02974424
```

Income

```r
alphaaovsIncomefull <- apply(alphas, 2, function(x) {
    mod <- lmer(x ~ Income4F + Library_sizelog10 + zyg + BMI +
        HEI + FIsqrt + Age + (1 | HEIv) + (1 | Frailty_Index_Vers) +
        (1 | SequencingRunID) + (1 | ExtractionPlateLoadedby) +
        (1 | Extractedby) + (1 | CollectionMethod), REML = F,
        data = mapalph)
    beta <- stdCoef.merMod(mod)
    sum <- summary(mod)
    list <- list(sum, beta)
    return(list)
})

alphaaovsIncomefull
```

```
## $Chao1
## $Chao1[[1]]
## Linear mixed model fit by maximum likelihood . t-tests use
##   Satterthwaite's method [lmerModLmerTest]
## Formula: x ~ Income4F + Library_sizelog10 + zyg + BMI + HEI + FIsqrt +
##     Age + (1 | HEIv) + (1 | Frailty_Index_Vers) + (1 | SequencingRunID) +
##     (1 | ExtractionPlateLoadedby) + (1 | Extractedby) + (1 |
##     CollectionMethod)
##    Data: mapalph
##
##      AIC      BIC   logLik deviance df.resid
##   1514.7   1594.3   -740.3   1480.7      782
##
## Scaled residuals:
##     Min      1Q  Median      3Q     Max
## -2.6882 -0.5934 -0.0770  0.4129  7.6091
##
## Random effects:
##  Groups                  Name        Variance Std.Dev.
##  SequencingRunID         (Intercept) 0.17575  0.4192
##  ExtractionPlateLoadedby (Intercept) 0.04555  0.2134
##  CollectionMethod        (Intercept) 0.00000  0.0000
##  Extractedby             (Intercept) 0.00000  0.0000
##  Frailty_Index_Vers      (Intercept) 0.00000  0.0000
##  HEIv                    (Intercept) 0.00000  0.0000
##  Residual                            0.33365  0.5776
## Number of obs: 799, groups:
## SequencingRunID, 33; ExtractionPlateLoadedby, 9; CollectionMethod, 2; Extractedby, 2; Frailty_Index_Vers
##
## Fixed effects:
##                     Estimate Std. Error         df t value Pr(>|t|)
## (Intercept)       -13.420557   0.702700 633.357245 -19.099   <2e-16 ***
## Income4F2           0.046896   0.066044 771.214793   0.710   0.4779
## Income4F3           0.131164   0.061236 767.849441   2.142   0.0325 *
## Income4F4           0.124552   0.072138 770.782965   1.727   0.0846 .
## Library_sizelog10   2.714510   0.140632 749.025930  19.302   <2e-16 ***
## zyg                -0.028192   0.021317 777.207269  -1.323   0.1864
## BMI                 0.004940   0.022670 771.604416   0.218   0.8276
```

```
## HEI                -0.028542   0.022311 769.146165  -1.279   0.2012
## FIsqrt             -0.020615   0.022413 772.752987  -0.920   0.3580
## Age                -0.001563   0.025942 778.398634  -0.060   0.9520
## ---
## Signif. codes:  0 '***' 0.001 '**' 0.01 '*' 0.05 '.' 0.1 ' ' 1
##
## Correlation of Fixed Effects:
##             (Intr) Inc4F2 Inc4F3 Inc4F4 Lbr_10 zyg    BMI    HEI    FIsqrt
## Income4F2   -0.102
## Income4F3   -0.139  0.646
## Income4F4   -0.100  0.559  0.614
## Lbrry_szl10 -0.985  0.045  0.078  0.046
## zyg         -0.041 -0.022  0.004  0.002  0.041
## BMI          0.009 -0.005  0.008  0.050 -0.010 -0.030
## HEI         -0.014 -0.012 -0.012 -0.049  0.015 -0.020  0.103
## FIsqrt      -0.024  0.043  0.044  0.107  0.014  0.032 -0.218  0.089
## Age         -0.109  0.076  0.181  0.173  0.099 -0.011 -0.011  0.040 -0.189
##
## $Chao1[[2]]
##                        stdcoef      stdse
## (Intercept)        0.000000000 0.00000000
## Income4F2          0.022384952 0.03152515
## Income4F3          0.070080964 0.03271837
## Income4F4          0.052915539 0.03064755
## Library_sizelog10  0.539262694 0.02793795
## zyg               -0.031015791 0.02345155
## BMI                0.005202733 0.02387486
## HEI               -0.029866628 0.02334647
## FIsqrt            -0.022744610 0.02472828
## Age               -0.001475317 0.02449140
##
##
## $Shannon
## $Shannon[[1]]
## Linear mixed model fit by maximum likelihood . t-tests use
##   Satterthwaite's method [lmerModLmerTest]
## Formula: x ~ Income4F + Library_sizelog10 + zyg + BMI + HEI + FIsqrt +
##     Age + (1 | HEIv) + (1 | Frailty_Index_Vers) + (1 | SequencingRunID) +
##     (1 | ExtractionPlateLoadedby) + (1 | Extractedby) + (1 |
##     CollectionMethod)
##    Data: mapalph
##
##      AIC      BIC   logLik deviance df.resid
##   2294.3   2374.0  -1130.2   2260.3      782
##
## Scaled residuals:
##     Min      1Q  Median      3Q     Max
## -5.3825 -0.4911  0.1209  0.6835  2.3522
##
## Random effects:
##  Groups                  Name        Variance Std.Dev.
##  SequencingRunID         (Intercept) 0.009857 0.09928
##  ExtractionPlateLoadedby (Intercept) 0.000000 0.00000
##  CollectionMethod        (Intercept) 0.000000 0.00000
```

```
##  Extractedby             (Intercept) 0.048006 0.21910
##  Frailty_Index_Vers      (Intercept) 0.037206 0.19289
##  HEIv                    (Intercept) 0.000000 0.00000
##  Residual                            0.973350 0.98659
## Number of obs: 799, groups:
## SequencingRunID, 33; ExtractionPlateLoadedby, 9; CollectionMethod, 2; Extractedby, 2; Frailty_Index_Vers
##
## Fixed effects:
##                    Estimate Std. Error        df t value Pr(>|t|)
## (Intercept)         0.29253    1.01527 219.34028   0.288   0.7735
## Income4F2           0.03073    0.11038 794.14940   0.278   0.7808
## Income4F3           0.15808    0.10393 786.10311   1.521   0.1287
## Income4F4           0.23905    0.12190 792.99730   1.961   0.0502 .
## Library_sizelog10  -0.05843    0.20199 240.55172  -0.289   0.7726
## zyg                 0.02428    0.03523 776.10959   0.689   0.4909
## BMI                -0.06523    0.03836 795.22083  -1.700   0.0895 .
## HEI                 0.07001    0.03757 796.55181   1.863   0.0628 .
## FIsqrt             -0.21859    0.04391 535.17974  -4.978 8.66e-07 ***
## Age                 0.10016    0.04440 776.49749   2.256   0.0244 *
## ---
## Signif. codes:  0 '***' 0.001 '**' 0.01 '*' 0.05 '.' 0.1 ' ' 1
##
## Correlation of Fixed Effects:
##             (Intr) Inc4F2 Inc4F3 Inc4F4 Lbr_10 zyg    BMI    HEI    FIsqrt
## Income4F2   -0.097
## Income4F3   -0.147  0.646
## Income4F4   -0.105  0.560  0.617
## Lbrry_szl10 -0.973  0.034  0.082  0.048
## zyg         -0.059 -0.038 -0.005 -0.013  0.057
## BMI          0.005 -0.021 -0.008  0.033 -0.006 -0.027
## HEI         -0.037 -0.010 -0.014 -0.048  0.046 -0.021  0.094
## FIsqrt      -0.037  0.083  0.095  0.140  0.035  0.000 -0.262  0.089
## Age         -0.072  0.055  0.144  0.150  0.059  0.008  0.035  0.035 -0.285
##
## $Shannon[[2]]
##                       stdcoef      stdse
## (Intercept)        0.00000000 0.00000000
## Income4F2          0.01302751 0.04678873
## Income4F3          0.07500460 0.04931232
## Income4F4          0.09018913 0.04599207
## Library_sizelog10 -0.01030778 0.03563516
## zyg                0.02372246 0.03441736
## BMI               -0.06100540 0.03588105
## HEI                0.06505485 0.03491342
## FIsqrt            -0.21417731 0.04302075
## Age                0.08397368 0.03722577
##
##
## $Simpson
## $Simpson[[1]]
## Linear mixed model fit by maximum likelihood . t-tests use
##   Satterthwaite's method [lmerModLmerTest]
## Formula: x ~ Income4F + Library_sizelog10 + zyg + BMI + HEI + FIsqrt +
##     Age + (1 | HEIv) + (1 | Frailty_Index_Vers) + (1 | SequencingRunID) +
```

```
##     (1 | ExtractionPlateLoadedby) + (1 | Extractedby) + (1 |
##     CollectionMethod)
##    Data: mapalph
##
##      AIC      BIC   logLik deviance df.resid
##   2406.2   2485.9  -1186.1   2372.2      782
##
## Scaled residuals:
##     Min      1Q  Median      3Q     Max
## -9.7878 -0.1460  0.2647  0.4976  1.1448
##
## Random effects:
##  Groups                  Name        Variance Std.Dev.
##  SequencingRunID         (Intercept) 0.009666 0.09832
##  ExtractionPlateLoadedby (Intercept) 0.000000 0.00000
##  CollectionMethod        (Intercept) 0.000000 0.00000
##  Extractedby             (Intercept) 0.038238 0.19555
##  Frailty_Index_Vers      (Intercept) 0.009110 0.09545
##  HEIv                    (Intercept) 0.000000 0.00000
##  Residual                            1.124030 1.06020
## Number of obs: 799, groups:
## SequencingRunID, 33; ExtractionPlateLoadedby, 9; CollectionMethod, 2; Extractedby, 2; Frailty_Index_Vers
##
## Fixed effects:
##                   Estimate Std. Error        df t value Pr(>|t|)
## (Intercept)        0.29571    1.07447 221.67869   0.275   0.7834
## Income4F2          0.11941    0.11843 792.93467   1.008   0.3136
## Income4F3          0.25264    0.11146 782.84652   2.267   0.0237 *
## Income4F4          0.33369    0.13078 792.92231   2.552   0.0109 *
## Library_sizelog10 -0.07452    0.21600 224.08537  -0.345   0.7304
## zyg                0.01036    0.03782 771.97995   0.274   0.7843
## BMI               -0.01807    0.04112 785.96608  -0.440   0.6604
## HEI                0.03492    0.04034 797.12731   0.866   0.3869
## FIsqrt            -0.10517    0.04526  85.68614  -2.324   0.0225 *
## Age                0.04180    0.04724 592.10216   0.885   0.3766
## ---
## Signif. codes:  0 '***' 0.001 '**' 0.01 '*' 0.05 '.' 0.1 ' ' 1
##
## Correlation of Fixed Effects:
##             (Intr) Inc4F2 Inc4F3 Inc4F4 Lbr_10 zyg    BMI    HEI    FIsqrt
## Income4F2   -0.098
## Income4F3   -0.149  0.645
## Income4F4   -0.105  0.559  0.616
## Lbrry_szl10 -0.984  0.033  0.081  0.046
## zyg         -0.060 -0.038 -0.004 -0.012  0.058
## BMI          0.004 -0.019 -0.004  0.036 -0.004 -0.028
## HEI         -0.038 -0.011 -0.015 -0.049  0.046 -0.020  0.095
## FIsqrt      -0.035  0.075  0.082  0.132  0.031  0.003 -0.254  0.089
## Age         -0.074  0.061  0.153  0.158  0.061  0.007  0.026  0.036 -0.260
##
## $Simpson[[2]]
##                        stdcoef      stdse
## (Intercept)        0.000000000 0.00000000
## Income4F2          0.048130895 0.04773628
```

```
## Income4F3          0.113986784 0.05028767
## Income4F4          0.119716262 0.04691978
## Library_sizelog10 -0.012501625 0.03623605
## zyg                0.009621254 0.03513133
## BMI               -0.016074167 0.03656662
## HEI                0.030857916 0.03564596
## FIsqrt            -0.097987378 0.04216472
## Age                0.033321952 0.03765846
```

IMD

```
alphaaovsIMDFfull <- apply(alphas, 2, function(x) {
    mod <- lmer(x ~ IMD5f + Library_sizelog10 + zyg + BMI + HEI +
        FIsqrt + Age + (1 | HEIv) + (1 | Frailty_Index_Vers) +
        (1 | SequencingRunID) + (1 | ExtractionPlateLoadedby) +
        (1 | Extractedby) + (1 | CollectionMethod), REML = F,
        data = mapalph)
    beta <- stdCoef.merMod(mod)
    sum <- summary(mod)
    list <- list(sum, beta)
    return(list)
})

alphaaovsIMDFfull
```

```
## $Chao1
## $Chao1[[1]]
## Linear mixed model fit by maximum likelihood . t-tests use
##   Satterthwaite's method [lmerModLmerTest]
## Formula: x ~ IMD5f + Library_sizelog10 + zyg + BMI + HEI + FIsqrt + Age +
##     (1 | HEIv) + (1 | Frailty_Index_Vers) + (1 | SequencingRunID) +
##     (1 | ExtractionPlateLoadedby) + (1 | Extractedby) + (1 |
##     CollectionMethod)
##    Data: mapalph
##
##      AIC      BIC   logLik deviance df.resid
##   3333.8   3431.4  -1648.9   3297.8     1654
##
## Scaled residuals:
##     Min      1Q  Median      3Q     Max
## -3.4409 -0.5444 -0.1090  0.3926  7.5158
##
## Random effects:
##  Groups                  Name        Variance  Std.Dev.
##  SequencingRunID         (Intercept) 0.2051439 0.45293
##  ExtractionPlateLoadedby (Intercept) 0.0354320 0.18823
##  CollectionMethod        (Intercept) 0.0012180 0.03490
##  Extractedby             (Intercept) 0.0000000 0.00000
##  Frailty_Index_Vers      (Intercept) 0.0001381 0.01175
##  HEIv                    (Intercept) 0.0000000 0.00000
##  Residual                            0.3927739 0.62672
## Number of obs: 1672, groups:
## SequencingRunID, 33; ExtractionPlateLoadedby, 10; CollectionMethod, 2; Extractedby, 2; Frailty_Index_Ve
```

```
##
## Fixed effects:
##                    Estimate Std. Error        df t value Pr(>|t|)
## (Intercept)       -1.354e+01  4.951e-01  1.154e+03 -27.345   <2e-16 ***
## IMD5f2            -1.297e-02  4.934e-02  1.639e+03  -0.263    0.793
## IMD5f3             5.727e-02  4.895e-02  1.637e+03   1.170    0.242
## IMD5f4             5.339e-02  4.944e-02  1.636e+03   1.080    0.280
## IMD5f5             3.930e-02  4.942e-02  1.638e+03   0.795    0.427
## Library_sizelog10  2.744e+00  9.852e-02  1.603e+03  27.857   <2e-16 ***
## zyg               -1.661e-02  1.600e-02  1.649e+03  -1.038    0.299
## BMI               -1.072e-03  1.638e-02  1.075e+03  -0.065    0.948
## HEI               -1.716e-02  1.575e-02  1.640e+03  -1.089    0.276
## FIsqrt            -1.757e-02  1.720e-02  1.084e+01  -1.021    0.329
## Age               -2.508e-03  1.655e-02  1.001e+02  -0.152    0.880
## ---
## Signif. codes:  0 '***' 0.001 '**' 0.01 '*' 0.05 '.' 0.1 ' ' 1
##
## Correlation of Fixed Effects:
##             (Intr) IMD5f2 IMD5f3 IMD5f4 IMD5f5 Lbr_10 zyg    BMI    HEI
## IMD5f2      -0.036
## IMD5f3      -0.025  0.495
## IMD5f4      -0.039  0.505  0.494
## IMD5f5      -0.022  0.506  0.499  0.511
## Lbrry_szl10 -0.972 -0.018 -0.025 -0.013 -0.027
## zyg         -0.021 -0.004 -0.019  0.008 -0.012  0.021
## BMI          0.002  0.078  0.042  0.104  0.090 -0.008 -0.032
## HEI          0.025 -0.037 -0.021 -0.022 -0.016 -0.022 -0.023  0.099
## FIsqrt      -0.005  0.026 -0.017 -0.009  0.011  0.002 -0.002 -0.248  0.064
## Age         -0.055 -0.040 -0.021 -0.059 -0.092  0.065 -0.096 -0.009  0.035
##             FIsqrt
## IMD5f2
## IMD5f3
## IMD5f4
## IMD5f5
## Lbrry_szl10
## zyg
## BMI
## HEI
## FIsqrt
## Age         -0.238
##
## $Chao1[[2]]
##                        stdcoef      stdse
## (Intercept)        0.000000000 0.00000000
## IMD5f2            -0.005179304 0.01971143
## IMD5f3             0.022903184 0.01957503
## IMD5f4             0.021352676 0.01977218
## IMD5f5             0.015733671 0.01978806
## Library_sizelog10  0.516081412 0.01852597
## zyg               -0.016610569 0.01600375
## BMI               -0.001072235 0.01638097
## HEI               -0.017161156 0.01575428
## FIsqrt            -0.017568425 0.01719962
## Age               -0.002507775 0.01655202
```

```
##
##
## $Shannon
## $Shannon[[1]]
## Linear mixed model fit by maximum likelihood . t-tests use
##   Satterthwaite's method [lmerModLmerTest]
## Formula: x ~ IMD5f + Library_sizelog10 + zyg + BMI + HEI + FIsqrt + Age +
##     (1 | HEIv) + (1 | Frailty_Index_Vers) + (1 | SequencingRunID) +
##     (1 | ExtractionPlateLoadedby) + (1 | Extractedby) + (1 |
##     CollectionMethod)
##    Data: mapalph
##
##      AIC      BIC   logLik deviance df.resid
##   4665.7   4763.3  -2314.9   4629.7     1654
##
## Scaled residuals:
##     Min      1Q  Median      3Q     Max
## -5.6320 -0.5143  0.1354  0.6871  2.1975
##
## Random effects:
##  Groups                  Name        Variance  Std.Dev.
##  SequencingRunID         (Intercept) 1.268e-02 1.126e-01
##  ExtractionPlateLoadedby (Intercept) 9.346e-16 3.057e-08
##  CollectionMethod        (Intercept) 3.259e-17 5.709e-09
##  Extractedby             (Intercept) 1.490e-02 1.221e-01
##  Frailty_Index_Vers      (Intercept) 2.172e-02 1.474e-01
##  HEIv                    (Intercept) 2.266e-17 4.761e-09
##  Residual                            9.205e-01 9.594e-01
## Number of obs: 1672, groups:
## SequencingRunID, 33; ExtractionPlateLoadedby, 10; CollectionMethod, 2; Extractedby, 2; Frailty_Index_Ve
##
## Fixed effects:
##                     Estimate Std. Error         df t value Pr(>|t|)
## (Intercept)          0.26152    0.67060  339.71808   0.390 0.696794
## IMD5f2               0.16306    0.07498 1661.07350   2.175 0.029784 *
## IMD5f3               0.06893    0.07453 1655.94636   0.925 0.355174
## IMD5f4               0.05529    0.07509 1660.59906   0.736 0.461696
## IMD5f5               0.08730    0.07516 1660.89158   1.161 0.245641
## Library_sizelog10   -0.06322    0.13453  462.83718  -0.470 0.638638
## zyg                  0.02174    0.02396 1643.60905   0.907 0.364436
## BMI                 -0.10469    0.02508 1666.51372  -4.175 3.14e-05 ***
## HEI                  0.08081    0.02391 1667.43504   3.379 0.000744 ***
## FIsqrt              -0.18405    0.02927 1163.16700  -6.287 4.57e-10 ***
## Age                  0.12942    0.02594 1577.10428   4.988 6.76e-07 ***
## ---
## Signif. codes:  0 '***' 0.001 '**' 0.01 '*' 0.05 '.' 0.1 ' ' 1
##
## Correlation of Fixed Effects:
##             (Intr) IMD5f2 IMD5f3 IMD5f4 IMD5f5 Lbr_10 zyg    BMI    HEI
## IMD5f2      -0.029
## IMD5f3      -0.035  0.499
## IMD5f4      -0.039  0.506  0.500
## IMD5f5      -0.029  0.507  0.501  0.510
## Lbrry_szl10 -0.973 -0.027 -0.021 -0.017 -0.027
```

```
## zyg         -0.055 -0.005 -0.020  0.005 -0.016  0.053
## BMI          0.001  0.077  0.039  0.096  0.086 -0.009 -0.031
## HEI          0.014 -0.037 -0.018 -0.019 -0.016 -0.009 -0.029  0.096
## FIsqrt      -0.006  0.029 -0.005  0.010  0.021  0.011 -0.018 -0.282  0.072
## Age         -0.041 -0.047 -0.033 -0.076 -0.103  0.047 -0.081  0.027  0.024
##             FIsqrt
## IMD5f2
## IMD5f3
## IMD5f4
## IMD5f5
## Lbrry_szl10
## zyg
## BMI
## HEI
## FIsqrt
## Age         -0.331
##
## $Shannon[[2]]
##                      stdcoef      stdse
## (Intercept)        0.00000000 0.00000000
## IMD5f2             0.06514210 0.02995278
## IMD5f3             0.02756813 0.02980793
## IMD5f4             0.02211146 0.03003347
## IMD5f5             0.03495281 0.03009505
## Library_sizelog10 -0.01188806 0.02529836
## zyg                0.02173887 0.02396270
## BMI               -0.10469142 0.02507768
## HEI                0.08081293 0.02391475
## FIsqrt            -0.18404781 0.02927388
## Age                0.12941992 0.02594445
##
##
## $Simpson
## $Simpson[[1]]
## Linear mixed model fit by maximum likelihood . t-tests use
##   Satterthwaite's method [lmerModLmerTest]
## Formula: x ~ IMD5f + Library_sizelog10 + zyg + BMI + HEI + FIsqrt + Age +
##     (1 | HEIv) + (1 | Frailty_Index_Vers) + (1 | SequencingRunID) +
##     (1 | ExtractionPlateLoadedby) + (1 | Extractedby) + (1 |
##     CollectionMethod)
##    Data: mapalph
##
##      AIC      BIC   logLik deviance df.resid
##   4738.2   4835.8  -2351.1   4702.2     1654
##
## Scaled residuals:
##      Min       1Q   Median       3Q      Max
## -10.8152  -0.1712   0.2656   0.5243   1.1992
##
## Random effects:
##  Groups                  Name        Variance Std.Dev.
##  SequencingRunID         (Intercept) 0.002145 0.04631
##  ExtractionPlateLoadedby (Intercept) 0.000000 0.00000
##  CollectionMethod        (Intercept) 0.000000 0.00000
```

```
##  Extractedby             (Intercept) 0.024535 0.15664
##  Frailty_Index_Vers      (Intercept) 0.008299 0.09110
##  HEIv                    (Intercept) 0.000000 0.00000
##  Residual                            0.968826 0.98429
## Number of obs: 1672, groups:
## SequencingRunID, 33; ExtractionPlateLoadedby, 10; CollectionMethod, 2; Extractedby, 2; Frailty_Index_Ve
##
## Fixed effects:
##                      Estimate Std. Error         df t value Pr(>|t|)
## (Intercept)        2.887e-01  6.542e-01  1.421e+02   0.441 0.659674
## IMD5f2             1.231e-01  7.670e-02  1.669e+03   1.605 0.108683
## IMD5f3             6.846e-02  7.630e-02  1.662e+03   0.897 0.369715
## IMD5f4             7.357e-02  7.683e-02  1.669e+03   0.958 0.338452
## IMD5f5             6.036e-02  7.690e-02  1.669e+03   0.785 0.432581
## Library_sizelog10 -5.238e-02  1.314e-01  1.549e+02  -0.399 0.690670
## zyg                9.025e-03  2.440e-02  1.535e+03   0.370 0.711470
## BMI               -6.095e-02  2.561e-02  1.645e+03  -2.380 0.017417 *
## HEI                5.372e-02  2.446e-02  1.670e+03   2.197 0.028187 *
## FIsqrt            -1.130e-01  2.943e-02  3.749e+02  -3.840 0.000145 ***
## Age                7.287e-02  2.627e-02  1.072e+03   2.773 0.005643 **
## ---
## Signif. codes:  0 '***' 0.001 '**' 0.01 '*' 0.05 '.' 0.1 ' ' 1
##
## Correlation of Fixed Effects:
##             (Intr) IMD5f2 IMD5f3 IMD5f4 IMD5f5 Lbr_10 zyg    BMI    HEI
## IMD5f2      -0.030
## IMD5f3      -0.040  0.500
## IMD5f4      -0.043  0.505  0.501
## IMD5f5      -0.032  0.505  0.501  0.508
## Lbrry_szl10 -0.974 -0.030 -0.019 -0.016 -0.027
## zyg         -0.062 -0.006 -0.020  0.003 -0.016  0.060
## BMI         -0.004  0.079  0.041  0.097  0.088 -0.004 -0.033
## HEI          0.015 -0.037 -0.019 -0.019 -0.016 -0.009 -0.029  0.097
## FIsqrt       0.001  0.027 -0.005  0.008  0.020  0.003 -0.016 -0.279  0.072
## Age         -0.039 -0.047 -0.035 -0.077 -0.105  0.045 -0.082  0.023  0.024
##             FIsqrt
## IMD5f2
## IMD5f3
## IMD5f4
## IMD5f5
## Lbrry_szl10
## zyg
## BMI
## HEI
## FIsqrt
## Age         -0.316
##
## $Simpson[[2]]
##                        stdcoef      stdse
## (Intercept)        0.000000000 0.00000000
## IMD5f2             0.049180643 0.03064218
## IMD5f3             0.027380877 0.03051642
## IMD5f4             0.029421758 0.03072760
## IMD5f5             0.024168764 0.03078929
```

```
## Library_sizelog10 -0.009849671 0.02470500
## zyg                 0.009024979 0.02439509
## BMI                -0.060953706 0.02560878
## HEI                 0.053718587 0.02445543
## FIsqrt             -0.113000112 0.02943027
## Age                 0.072868552 0.02627366
```

Extracting coefficients for results tables

```
extract_results <- function(x) {
    list <- lapply(x, function(y) {
        stcoef <- y[[2]]$stdcoef
        vals <- y[[1]]$coefficients[, 5]
        z <- cbind(stcoef, vals)
        return(z)
    })
    df <- as.data.frame(list)
    df$varb <- row.names(df)
    return(df)
}

othervarbs <- extract_results(nonses)

educ1 <- extract_results(alphaaovsEDUF)
inc1 <- extract_results(alphaaovsIncome)
imd1 <- extract_results(alphaaovsIMDF)

edu2 <- extract_results(alphaaovsEducfull)
inc2 <- extract_results(alphaaovsIncomefull)
imd2 <- extract_results(alphaaovsIMDFfull)


t0 <- merge(othervarbs, educ1, by = "varb", all = T)
t1 <- merge(t0, inc1, by = "varb", all = T)
t2 <- merge(t1, imd1, by = "varb", all = T)
t3 <- merge(t2, edu2, by = "varb", all = T)
t4 <- merge(t3, inc2, by = "varb", all = T)
all <- merge(t4, imd2, by = "varb", all = T)


x <- all[c(2:43)]

all <- as.data.frame(ifelse(grepl("stcoef", names(x)), round(x,
    digits = 2), round(x, digits = 3)), row.names = all$varb,
    col.names = names(x))



write.csv.date(all, "alpha_diversity_SES")

modelAICs <- function(x) {
    list <- lapply(x, function(y) {
```

```
        AICval <- y[[1]]$AICtab[1]
    })
    as.data.frame(list)
}

othervars <- modelAICs(nonses)

educ1 <- modelAICs(alphaaovsEDUF)
inc1 <- modelAICs(alphaaovsIncome)
imd1 <- modelAICs(alphaaovsIMDF)

edu2 <- modelAICs(alphaaovsEducfull)
inc2 <- modelAICs(alphaaovsIncomefull)
imd2 <- modelAICs(alphaaovsIMDFfull)

aics <- rbind(othervars, educ1, inc1, imd1, edu2, inc2, imd2)

row.names(aics) <- c("Non.ses", "educ1", "inc1", "imd1", "edu2",
    "inc2", "imd2")

write.csv.date(aics, "alpha_diversity_aics")
```

Packages used

```
# For creation of alpha diversity estimates:
citation("phyloseq")
```

To cite phyloseq in publications, or otherwise credit, please use:

phyloseq: An R package for reproducible interactive analysis and graphics of microbiome census data. Paul J. McMurdie and Susan Holmes (2013) PLoS ONE 8(4):e61217.

A BibTeX entry for LaTeX users is

@Article{, author = {Paul J. McMurdie and Susan Holmes}, journal = {PLoS ONE}, pages = {e61217}, title = {phyloseq: An R package for reproducible interactive analysis and graphics of microbiome census data}, volume = {8}, number = {4}, year = {2013}, url = {http://dx.plos.org/10.1371/journal.pone.0061217}, }

```
# For mixed effects models:
citation("lmerTest")
```

To cite lmerTest in publications use:

Kuznetsova A, Brockhoff PB, Christensen RHB (2017). “lmerTest Package: Tests in Linear Mixed Effects Models.” *Journal of Statistical Software*, *82*(13), 1-26. doi: 10.18637/jss.v082.i13 (URL: http://doi.org/10.18637/jss.v082.i13).

A BibTeX entry for LaTeX users is

@Article{, title = {{lmerTest} Package: Tests in Linear Mixed Effects Models}, author = {Alexandra Kuznetsova and Per B. Brockhoff and Rune H. B. Christensen}, journal = {Journal of Statistical Software}, year = {2017}, volume = {82}, number = {13}, pages = {1–26}, doi = {10.18637/jss.v082.i13}, }
