# Supplementary material for: Socioeconomic Status and the Gut Microbiome: A TwinsUK Cohort Study
Source: Microorganisms. 2019 Jan 11;7(1):17. doi: 10.3390/microorganisms7010017 (PMC6351927; doi:10.3390/microorganisms7010017)
Supplement: Supplementary file 1 [file microorganisms-07-00017-s001.zip › Additional File 4.pdf]

SES & the microbiome 3.1: Beta Diversity & Permanova

December 04, 2018

```
library(phyloseq)
library(data.table)
library(ggplot2)
library(ape)

otus <- read.table("Variance_transformed_SEStrimmed5%OTU_table.txt",
    sep = " ", row.names = 1, header = T)
tree <- read.tree("microbiome_tables/phyloseq_tables_aug2017/reduced_tree.tre")
tax <- read.csv("taxa_SES_trimmed5%.csv", row.names = 1)
mapping <- read.csv("mapping_ses_correctpcdes041218.csv")
mapping$IMD5f <- as.factor(mapping$IMD5f)
mapping$eduff <- as.factor(mapping$eduff)
mapping$Income4F <- as.factor(mapping$Income4F)
mapping$FIsqrt <- sqrt(mapping$FI)

OTU <- otu_table(otus, taxa_are_rows = T)
tax <- as.matrix(tax)
TAX <- tax_table(tax)
physeq <- phyloseq(OTU, TAX)

mapping <- sample_data(mapping)
row.names(mapping) <- mapping$SequencingSpecificName

physeq1 <- merge_phyloseq(physeq, mapping, tree)
```

IMD - Bray

```
require(vegan)
metadata <- as(sample_data(physeq1), "data.frame")

metadata1 <- metadata[complete.cases(metadata$FIsqrt, metadata$Age,
    metadata$HEI, metadata$BMI), ]

map <- sample_data(physeq1)
bray <- vegdist(t(otus), "bray")
bray <- as.dist(as(bray, "matrix"))
Ord.bray <- ordinate(physeq1, method = "MDS", distance = bray)

braym <- as.matrix(bray)
braym <- as.data.frame(braym)
bray1 <- braym[which(row.names(braym) %in% metadata1$SequencingSpecificName),
    ]
bray1 <- bray1[, which(names(bray1) %in% metadata1$SequencingSpecificName)]
bray1 <- as.dist(as(bray1, "matrix"))

set.seed(1)
```

```r
adonis(bray1 ~ IMD5f, data = metadata1, permutations = 5000)
```

```
##
## Call:
## adonis(formula = bray1 ~ IMD5f, data = metadata1, permutations = 5000)
##
## Permutation: free
## Number of permutations: 5000
##
## Terms added sequentially (first to last)
##
##             Df SumsOfSqs  MeanSqs F.Model      R2   Pr(>F)
## IMD5f        4     0.174 0.043505  1.3729 0.00328 0.008398 **
## Residuals 1667    52.823 0.031687         0.99672
## Total     1671    52.997                  1.00000
## ---
## Signif. codes:  0 '***' 0.001 '**' 0.01 '*' 0.05 '.' 0.1 ' ' 1
```

```r
adonis(bray1 ~ IMD5f + FI + Age + BMI + HEI + Library_sizelog10,
    data = metadata1, permutations = 5000)
```

```
##
## Call:
## adonis(formula = bray1 ~ IMD5f + FI + Age + BMI + HEI + Library_sizelog10,     data = metadata1, permutatio
##
## Permutation: free
## Number of permutations: 5000
##
## Terms added sequentially (first to last)
##
##                     Df SumsOfSqs MeanSqs F.Model      R2   Pr(>F)
## IMD5f                4     0.174 0.04351  1.4051 0.00328 0.005999 **
## FI                   1     0.273 0.27258  8.8035 0.00514 0.000200 ***
## Age                  1     0.207 0.20742  6.6991 0.00391 0.000200 ***
## BMI                  1     0.226 0.22592  7.2966 0.00426 0.000200 ***
## HEI                  1     0.142 0.14222  4.5933 0.00268 0.000200 ***
## Library_sizelog10    1     0.515 0.51461 16.6204 0.00971 0.000200 ***
## Residuals         1662    51.460 0.03096         0.97100
## Total             1671    52.997                 1.00000
## ---
## Signif. codes:  0 '***' 0.001 '**' 0.01 '*' 0.05 '.' 0.1 ' ' 1
```

Dispersion

```r
beta <- betadisper(bray1, metadata1$IMD5f)
permutest(beta)
```

```
##
## Permutation test for homogeneity of multivariate dispersions
## Permutation: free
## Number of permutations: 999
##
## Response: Distances
```

```
##             Df  Sum Sq    Mean Sq      F N.Perm Pr(>F)
## Groups       4 0.00174 0.00043520 0.5667    999  0.664
## Residuals 1667 1.28027 0.00076801
```

IMD - W. Unifrac

```
metadata1 <- metadata[complete.cases(metadata$FIsqrt, metadata$Age,
    metadata$HEI, metadata$BMI), ]

w.unifrac <- read.table("distance_matrixes/weigthed_unifrac_phyloseq_rooted_tree.txt")

wum <- as.matrix(w.unifrac)
wum <- as.data.frame(wum)
wum1 <- wum[which(row.names(wum) %in% metadata1$SequencingSpecificName),
    ]
wum1 <- wum1[, which(names(wum1) %in% metadata1$SequencingSpecificName)]
wum1 <- as.dist(as(wum1, "matrix"))

adonis(wum1 ~ IMD5f, data = metadata1, permutations = 5000)
```

```
##
## Call:
## adonis(formula = wum1 ~ IMD5f, data = metadata1, permutations = 5000)
##
## Permutation: free
## Number of permutations: 5000
##
## Terms added sequentially (first to last)
##
##             Df SumsOfSqs  MeanSqs F.Model      R2  Pr(>F)
## IMD5f        4    0.0593 0.014836  1.3494 0.00323 0.03139 *
## Residuals 1667   18.3288 0.010995         0.99677
## Total     1671   18.3882                  1.00000
## ---
## Signif. codes:  0 '***' 0.001 '**' 0.01 '*' 0.05 '.' 0.1 ' ' 1
```

```
full.wum.IMD.adonis <- adonis(wum1 ~ IMD5f + FI + Age + BMI +
    HEI + Library_sizelog10, data = metadata1, permutations = 5000)
full.wum.IMD.adonis
```

```
##
## Call:
## adonis(formula = wum1 ~ IMD5f + FI + Age + BMI + HEI + Library_sizelog10,     data = metadata1, permutation
##
## Permutation: free
## Number of permutations: 5000
##
## Terms added sequentially (first to last)
##
##                     Df SumsOfSqs  MeanSqs F.Model      R2 Pr(>F)
## IMD5f                4    0.0593 0.014836  1.3788 0.00323 0.0232 *
## FI                   1    0.1142 0.114238 10.6165 0.00621 0.0002 ***
## Age                  1    0.0950 0.094979  8.8267 0.00517 0.0002 ***
## BMI                  1    0.0925 0.092469  8.5935 0.00503 0.0002 ***
## HEI                  1    0.0456 0.045648  4.2422 0.00248 0.0002 ***
## Library_sizelog10    1    0.0977 0.097719  9.0814 0.00531 0.0002 ***
## Residuals         1662   17.8838 0.010760         0.97257
## Total             1671   18.3882                  1.00000
```

```
## ---
## Signif. codes:  0 '***' 0.001 '**' 0.01 '*' 0.05 '.' 0.1 ' ' 1
```

Dispersion

```
beta <- betadisper(wum1, metadata1$IMD5f)
permutest(beta)
```

```
##
## Permutation test for homogeneity of multivariate dispersions
## Permutation: free
## Number of permutations: 999
##
## Response: Distances
##             Df  Sum Sq    Mean Sq      F N.Perm Pr(>F)
## Groups       4 0.00066 0.00016396 0.4928    999  0.761
## Residuals 1667 0.55465 0.00033272
```

Education - Bray

```
metadata2 <- metadata[complete.cases(metadata$FIsqrt, metadata$Age,
    metadata$HEI, metadata$BMI, metadata$eduff), ]

bray2 <- braym[which(row.names(braym) %in% metadata2$SequencingSpecificName),
    ]
bray2 <- bray2[, which(names(bray2) %in% metadata2$SequencingSpecificName)]
bray2 <- as.dist(as(bray2, "matrix"))

a1.eduff <- adonis(bray2 ~ eduff, data = metadata2, permutations = 5000)
a1.eduff
```

```
##
## Call:
## adonis(formula = bray2 ~ eduff, data = metadata2, permutations = 5000)
##
## Permutation: free
## Number of permutations: 5000
##
## Terms added sequentially (first to last)
##
##             Df SumsOfSqs  MeanSqs F.Model     R2    Pr(>F)
## eduff        3     0.162 0.054114  1.7143 0.0036 0.0003999 ***
## Residuals 1422    44.887 0.031566         0.9964
## Total     1425    45.049                  1.0000
## ---
## Signif. codes:  0 '***' 0.001 '**' 0.01 '*' 0.05 '.' 0.1 ' ' 1
```

```
full.bray.eduff.adonis <- adonis(bray2 ~ eduff + FI + Age + BMI +
    HEI + Library_sizelog10, data = metadata2, permutations = 5000)
```

Dispersion

```
beta <- betadisper(bray2, metadata2$eduff)
permutest(beta)
```

```
##
## Permutation test for homogeneity of multivariate dispersions
## Permutation: free
## Number of permutations: 999
##
## Response: Distances
##             Df  Sum Sq    Mean Sq      F N.Perm Pr(>F)
## Groups       3 0.00238 0.00079308 1.0196    999   0.42
## Residuals 1422 1.10606 0.00077782
```

Education - W. unifrac

```
metadata2 <- metadata[complete.cases(metadata$FIsqrt, metadata$Age,
    metadata$HEI, metadata$BMI, metadata$eduff), ]


wum2 <- wum[which(row.names(wum) %in% metadata2$SequencingSpecificName),
    ]
wum2 <- wum2[, which(names(wum2) %in% metadata2$SequencingSpecificName)]
wum2 <- as.dist(as(wum2, "matrix"))

a1.eduff <- adonis(wum2 ~ eduff, data = metadata2, permutations = 5000)
a1.eduff
```

```
##
## Call:
## adonis(formula = wum2 ~ eduff, data = metadata2, permutations = 5000)
##
## Permutation: free
## Number of permutations: 5000
##
## Terms added sequentially (first to last)
##
##             Df SumsOfSqs  MeanSqs F.Model      R2 Pr(>F)
## eduff        3     0.057 0.018994  1.7388 0.00365 0.0022 **
## Residuals 1422    15.534 0.010924         0.99635
## Total     1425    15.591                  1.00000
## ---
## Signif. codes:  0 '***' 0.001 '**' 0.01 '*' 0.05 '.' 0.1 ' ' 1
```

```
full.wum.eduff.adonis <- adonis(wum2 ~ eduff + FI + Age + BMI +
    HEI + Library_sizelog10, data = metadata2, permutations = 5000)

full.wum.eduff.adonis
```

```
##
## Call:
## adonis(formula = wum2 ~ eduff + FI + Age + BMI + HEI + Library_sizelog10,     data = metadata2, permutation
##
## Permutation: free
## Number of permutations: 5000
##
## Terms added sequentially (first to last)
##
##                     Df SumsOfSqs  MeanSqs F.Model      R2   Pr(>F)
## eduff                3    0.0570 0.018994  1.7729 0.00365 0.002799 **
## FI                   1    0.1072 0.107243 10.0098 0.00688 0.000200 ***
## Age                  1    0.0634 0.063370  5.9148 0.00406 0.000200 ***
## BMI                  1    0.0654 0.065376  6.1020 0.00419 0.000200 ***
## HEI                  1    0.0390 0.038951  3.6356 0.00250 0.000200 ***
## Library_sizelog10    1    0.0771 0.077140  7.2001 0.00495 0.000200 ***
## Residuals         1417   15.1814 0.010714         0.97376
## Total             1425   15.5905                  1.00000
## ---
## Signif. codes:  0 '***' 0.001 '**' 0.01 '*' 0.05 '.' 0.1 ' ' 1
```

Dispersion

```r
beta <- betadisper(wum2, metadata2$eduff)
permutest(beta)
```

```
##
## Permutation test for homogeneity of multivariate dispersions
## Permutation: free
## Number of permutations: 999
##
## Response: Distances
##             Df  Sum Sq    Mean Sq      F N.Perm Pr(>F)
## Groups       3 0.00074 0.00024600 0.7324    999  0.532
## Residuals 1422 0.47761 0.00033587
```

Income - Bray

```
metadata3 <- metadata[complete.cases(metadata$FIsqrt, metadata$Age,
    metadata$HEI, metadata$BMI, metadata$Income4F), ]


bray3 <- braym[which(row.names(braym) %in% metadata3$SequencingSpecificName),
    ]
bray3 <- bray3[, which(names(bray3) %in% metadata3$SequencingSpecificName)]
bray3 <- as.dist(as(bray3, "matrix"))

adonis(bray3 ~ Income4F, data = metadata3, permutations = 5000)
```

```
##
## Call:
## adonis(formula = bray3 ~ Income4F, data = metadata3, permutations = 5000)
##
## Permutation: free
## Number of permutations: 5000
##
## Terms added sequentially (first to last)
##
##            Df SumsOfSqs  MeanSqs F.Model      R2  Pr(>F)
## Income4F    3    0.1163 0.038767  1.2188 0.00458 0.06639 .
## Residuals 795   25.2872 0.031808         0.99542
## Total     798   25.4035                  1.00000
## ---
## Signif. codes:  0 '***' 0.001 '**' 0.01 '*' 0.05 '.' 0.1 ' ' 1
```

```
full.bray.Income.adonis <- adonis(bray3 ~ Income4F + FI + Age +
    BMI + HEI + Library_sizelog10, data = metadata3, permutations = 5000)
full.bray.Income.adonis
```

```
##
## Call:
## adonis(formula = bray3 ~ Income4F + FI + Age + BMI + HEI + Library_sizelog10,     data = metadata3, permuta[illegible]
##
## Permutation: free
## Number of permutations: 5000
##
## Terms added sequentially (first to last)
##
##                    Df SumsOfSqs  MeanSqs F.Model      R2    Pr(>F)
## Income4F            3    0.1163 0.038767  1.2432 0.00458 0.0507898 .
## FI                  1    0.1649 0.164939  5.2895 0.00649 0.0002000 ***
## Age                 1    0.0805 0.080527  2.5825 0.00317 0.0005999 ***
## BMI                 1    0.0933 0.093277  2.9914 0.00367 0.0002000 ***
## HEI                 1    0.0682 0.068187  2.1867 0.00268 0.0029994 **
## Library_sizelog10   1    0.2465 0.246498  7.9051 0.00970 0.0002000 ***
## Residuals         790   24.6338 0.031182         0.96970
## Total             798   25.4035                  1.00000
## ---
## Signif. codes:  0 '***' 0.001 '**' 0.01 '*' 0.05 '.' 0.1 ' ' 1
```

Dispersion

```
##
## Permutation test for homogeneity of multivariate dispersions
## Permutation: free
## Number of permutations: 999
##
## Response: Distances
##            Df  Sum Sq    Mean Sq      F N.Perm Pr(>F)
## Groups      3 0.00553 0.00184286 2.3465    999  0.094 .
## Residuals 795 0.62437 0.00078537
## ---
## Signif. codes:  0 '***' 0.001 '**' 0.01 '*' 0.05 '.' 0.1 ' ' 1
```

Income - W. Unifrac

```
metadata3 <- metadata[complete.cases(metadata$FIsqrt, metadata$Age,
    metadata$HEI, metadata$BMI, metadata$Income4F), ]


wum3 <- wum[which(row.names(wum) %in% metadata3$SequencingSpecificName),
    ]
wum3 <- wum3[, which(names(wum3) %in% metadata3$SequencingSpecificName)]
wum3 <- as.dist(as(wum3, "matrix"))

adonis(wum3 ~ Income4F, data = metadata3, permutations = 5000)
```

```
##
## Call:
## adonis(formula = wum3 ~ Income4F, data = metadata3, permutations = 5000)
##
## Permutation: free
## Number of permutations: 5000
##
## Terms added sequentially (first to last)
##
##            Df SumsOfSqs  MeanSqs F.Model      R2 Pr(>F)
## Income4F    3    0.0387 0.012894  1.1736 0.00441 0.1504
## Residuals 795    8.7346 0.010987         0.99559
## Total     798    8.7733                  1.00000
```

```
adonis(wum3 ~ Income4F + FI + Age + BMI + HEI + Library_sizelog10,
    data = metadata3, permutations = 5000)
```

```
##
## Call:
## adonis(formula = wum3 ~ Income4F + FI + Age + BMI + HEI + Library_sizelog10,     data = metadata3, permutat
##
## Permutation: free
## Number of permutations: 5000
##
## Terms added sequentially (first to last)
##
##                    Df SumsOfSqs  MeanSqs F.Model      R2    Pr(>F)
## Income4F            3    0.0387 0.012894  1.1934 0.00441 0.1339732
## FI                  1    0.0624 0.062419  5.7771 0.00711 0.0002000 ***
## Age                 1    0.0234 0.023428  2.1684 0.00267 0.0073985 **
## BMI                 1    0.0353 0.035334  3.2703 0.00403 0.0005999 ***
## HEI                 1    0.0227 0.022715  2.1024 0.00259 0.0089982 **
## Library_sizelog10   1    0.0551 0.055124  5.1019 0.00628 0.0002000 ***
## Residuals         790    8.5356 0.010805         0.97291
## Total             798    8.7733                  1.00000
## ---
## Signif. codes:  0 '***' 0.001 '**' 0.01 '*' 0.05 '.' 0.1 ' ' 1
```

Dispersion

```r
beta <- betadisper(wum3, metadata3$Income4F)
permutest(beta)
```

```
##
## Permutation test for homogeneity of multivariate dispersions
## Permutation: free
## Number of permutations: 999
##
## Response: Distances
##            Df   Sum Sq    Mean Sq      F N.Perm Pr(>F)
## Groups      3 0.002492 0.00083080 2.4162    999  0.065 .
## Residuals 795 0.273361 0.00034385
## ---
## Signif. codes:  0 '***' 0.001 '**' 0.01 '*' 0.05 '.' 0.1 ' ' 1
```

Packages used

```r
citation("phyloseq")
```

```
##
## To cite phyloseq in publications, or otherwise credit, please use:
##
##   phyloseq: An R package for reproducible interactive analysis and
##   graphics of microbiome census data. Paul J. McMurdie and Susan
##   Holmes (2013) PLoS ONE 8(4):e61217.
##
## A BibTeX entry for LaTeX users is
##
##   @Article{,
##     author = {Paul J. McMurdie and Susan Holmes},
##     journal = {PLoS ONE},
##     pages = {e61217},
##    title = {phyloseq: An R package for reproducible interactive analysis and graphics of microbiome census
##     volume = {8},
##     number = {4},
##     year = {2013},
##     url = {http://dx.plos.org/10.1371/journal.pone.0061217},
##   }
```

```r
citation("vegan")
```

```
##
## To cite package 'vegan' in publications use:
##
##   Jari Oksanen, F. Guillaume Blanchet, Michael Friendly, Roeland
##   Kindt, Pierre Legendre, Dan McGlinn, Peter R. Minchin, R. B.
##   O'Hara, Gavin L. Simpson, Peter Solymos, M. Henry H. Stevens,
##   Eduard Szoecs and Helene Wagner (2018). vegan: Community Ecology
##   Package. R package version 2.5-2.
##   https://CRAN.R-project.org/package=vegan
##
```

```
## A BibTeX entry for LaTeX users is
##
##   @Manual{,
##     title = {vegan: Community Ecology Package},
##    author = {Jari Oksanen and F. Guillaume Blanchet and Michael Friendly and Roeland Kindt and Pierre Leger
##     year = {2018},
##     note = {R package version 2.5-2},
##     url = {https://CRAN.R-project.org/package=vegan},
##   }
##
## ATTENTION: This citation information has been auto-generated from
## the package DESCRIPTION file and may need manual editing, see
## 'help("citation")'.
```

```r
# For creation of Rmarkdown files
citation("rmarkdown")
```

```
##
## To cite package 'rmarkdown' in publications use:
##
##   JJ Allaire, Yihui Xie, Jonathan McPherson, Javier Luraschi,
##   Kevin Ushey, Aron Atkins, Hadley Wickham, Joe Cheng and Winston
##   Chang (2018). rmarkdown: Dynamic Documents for R. R package
##   version 1.10. https://CRAN.R-project.org/package=rmarkdown
##
## A BibTeX entry for LaTeX users is
##
##   @Manual{,
##     title = {rmarkdown: Dynamic Documents for R},
##    author = {JJ Allaire and Yihui Xie and Jonathan McPherson and Javier Luraschi and Kevin Ushey and Aron A
##     year = {2018},
##     note = {R package version 1.10},
##     url = {https://CRAN.R-project.org/package=rmarkdown},
##   }
```

```r
citation("kableExtra")
```

```
##
## To cite package 'kableExtra' in publications use:
##
##   Hao Zhu (2018). kableExtra: Construct Complex Table with 'kable'
##   and Pipe Syntax. R package version 0.9.0.
##   https://CRAN.R-project.org/package=kableExtra
##
## A BibTeX entry for LaTeX users is
##
##   @Manual{,
##     title = {kableExtra: Construct Complex Table with 'kable' and Pipe Syntax},
##     author = {Hao Zhu},
##     year = {2018},
##     note = {R package version 0.9.0},
##     url = {https://CRAN.R-project.org/package=kableExtra},
##   }
```

```r
citation("knitr")
```

```
##
## To cite the 'knitr' package in publications use:
##
##   Yihui Xie (2018). knitr: A General-Purpose Package for Dynamic
##   Report Generation in R. R package version 1.20.
##
##   Yihui Xie (2015) Dynamic Documents with R and knitr. 2nd
##   edition. Chapman and Hall/CRC. ISBN 978-1498716963
##
##   Yihui Xie (2014) knitr: A Comprehensive Tool for Reproducible
##   Research in R. In Victoria Stodden, Friedrich Leisch and Roger
##   D. Peng, editors, Implementing Reproducible Computational
##   Research. Chapman and Hall/CRC. ISBN 978-1466561595
##
## To see these entries in BibTeX format, use 'print(<citation>,
## bibtex=TRUE)', 'toBibtex(.)', or set
## 'options(citation.bibtex.max=999)'.
```
