# Supplementary material for: Socioeconomic Status and the Gut Microbiome: A TwinsUK Cohort Study
Source: Microorganisms. 2019 Jan 11;7(1):17. doi: 10.3390/microorganisms7010017 (PMC6351927; doi:10.3390/microorganisms7010017)
Supplement: Supplementary file 1 [file microorganisms-07-00017-s001.zip › Additional File 5.pdf]

5.2b SES and Microbiome - twin discordance

LESS STRINGENT

December 06, 2018

Paired discordance of OTUs using EdgeR

Education

```
## Coefficient:  DiscordanceLDep
##                Kingdom            Phylum                  Class
## denovo604 k__Bacteria     p__Firmicutes          c__Clostridia
## denovo495 k__Bacteria     p__Firmicutes          c__Clostridia
## denovo88  k__Bacteria     p__Firmicutes          c__Clostridia
## denovo165 k__Bacteria     p__Firmicutes          c__Clostridia
## denovo8   k__Bacteria  p__Bacteroidetes         c__Bacteroidia
## denovo76  k__Bacteria     p__Firmicutes          c__Clostridia
## denovo111 k__Bacteria     p__Firmicutes          c__Clostridia
## denovo173 k__Bacteria    p__Tenericutes          c__Mollicutes
## denovo4   k__Bacteria p__Proteobacteria c__Gammaproteobacteria
## denovo40  k__Bacteria  p__Bacteroidetes         c__Bacteroidia
##                          Order                Family          Genus
## denovo604     o__Clostridiales                   f__            g__
## denovo495     o__Clostridiales    f__Lachnospiraceae            g__
## denovo88      o__Clostridiales    f__Ruminococcaceae            g__
## denovo165     o__Clostridiales    f__Lachnospiraceae            g__
## denovo8       o__Bacteroidales     f__Prevotellaceae   g__Prevotella
## denovo76      o__Clostridiales    f__Ruminococcaceae            g__
## denovo111     o__Clostridiales    f__Ruminococcaceae g__Ruminococcus
## denovo173              o__RF39                   f__            g__
## denovo4   o__Enterobacteriales f__Enterobacteriaceae            g__
## denovo40      o__Bacteroidales     f__Bacteroidaceae  g__Bacteroides
##              Species      logFC   logCPM        LR       PValue        FDR
## denovo604        s__  1.0889136 10.79897 12.828708 0.0003413414 0.01945646
## denovo495        s__ -0.9943345 10.88653  9.477717 0.0020798264 0.05927505
## denovo88         s__ -0.7623931 14.89692  3.543339 0.0597850054 0.90585455
## denovo165        s__ -0.5560401 12.80012  3.441739 0.0635687404 0.90585455
## denovo8    s__copri  0.8544375 18.74675  1.715070 0.1903288677 0.97914438
## denovo76         s__  0.7142574 14.88601  1.602759 0.2055126174 0.97914438
## denovo111        s__ -0.4716200 13.97125  1.345434 0.2460779338 0.97914438
## denovo173        s__  0.5744391 11.68180  1.305799 0.2531567358 0.97914438
```

```
## denovo4         s__  0.7545204 18.61378  1.208533 0.2716228045 0.97914438
## denovo40        s__  0.5171439 14.99081  1.173106 0.2787638763 0.97914438
```

Income

```
## Coefficient:  DiscordanceLDep
##                Kingdom             Phylum               Class
## denovo400  k__Bacteria      p__Firmicutes  c__Erysipelotrichi
## denovo2    k__Bacteria p__Verrucomicrobia c__Verrucomicrobiae
## denovo77   k__Bacteria   p__Bacteroidetes      c__Bacteroidia
## denovo1750 k__Bacteria      p__Firmicutes       c__Clostridia
## denovo469  k__Bacteria      p__Firmicutes       c__Clostridia
## denovo165  k__Bacteria      p__Firmicutes       c__Clostridia
## denovo495  k__Bacteria      p__Firmicutes       c__Clostridia
## denovo22   k__Bacteria  p__Actinobacteria   c__Actinobacteria
## denovo99   k__Bacteria      p__Firmicutes       c__Clostridia
## denovo60   k__Bacteria      p__Firmicutes          c__Bacilli
##                               Order                   Family
## denovo400  o__Erysipelotrichales f__Erysipelotrichaceae
## denovo2    o__Verrucomicrobiales f__Verrucomicrobiaceae
## denovo77        o__Bacteroidales      f__Prevotellaceae
## denovo1750      o__Clostridiales     f__Lachnospiraceae
## denovo469       o__Clostridiales     f__Ruminococcaceae
## denovo165       o__Clostridiales     f__Lachnospiraceae
## denovo495       o__Clostridiales     f__Lachnospiraceae
## denovo22    o__Bifidobacteriales  f__Bifidobacteriaceae
## denovo99        o__Clostridiales     f__Veillonellaceae
## denovo60      o__Lactobacillales    f__Streptococcaceae
##                         Genus         Species       logFC   logCPM
## denovo400     g__Coprobacillus             s__  0.61071044 11.92574
## denovo2          g__Akkermansia  s__muciniphila -0.98232046 20.33570
## denovo77          g__Prevotella             s__  0.68584996 14.83980
## denovo1750   g__[Ruminococcus]       s__gnavus -0.44213558 10.86009
## denovo469  g__Faecalibacterium  s__prausnitzii  0.64753237 14.46680
## denovo165                  g__             s__  0.36110820 14.79665
## denovo495                  g__             s__ -0.35576261 12.59660
## denovo22    g__Bifidobacterium s__adolescentis  0.21246331 18.39142
## denovo99    g__Acidaminococcus             s__ -0.14984141 15.61010
## denovo60      g__Streptococcus             s__  0.03127444 15.41680
##                     LR     PValue       FDR
## denovo400  2.898575401 0.08865787 0.4918161
## denovo2    2.214951718 0.13667934 0.4918161
## denovo77   2.097451911 0.14754484 0.4918161
## denovo1750 1.479406450 0.22386741 0.5497926
```

```
## denovo469  1.192150139 0.27489628 0.5497926
## denovo165  0.823180827 0.36425181 0.5886087
## denovo495  0.672946809 0.41202610 0.5886087
## denovo22   0.135267296 0.71303204 0.8912901
## denovo99   0.055049374 0.81449900 0.9049989
## denovo60   0.006319754 0.93663742 0.9366374
```

IMD

```
## Coefficient:  DiscordanceLDep
##                Kingdom           Phylum         Class           Order
## denovo221 k__Bacteria p__Bacteroidetes c__Bacteroidia o__Bacteroidales
## denovo114 k__Bacteria p__Bacteroidetes c__Bacteroidia o__Bacteroidales
## denovo106 k__Bacteria    p__Firmicutes  c__Clostridia o__Clostridiales
## denovo281 k__Bacteria p__Bacteroidetes c__Bacteroidia o__Bacteroidales
## denovo91  k__Bacteria p__Bacteroidetes c__Bacteroidia o__Bacteroidales
## denovo268 k__Bacteria p__Bacteroidetes c__Bacteroidia o__Bacteroidales
## denovo958 k__Bacteria    p__Firmicutes  c__Clostridia o__Clostridiales
## denovo110 k__Bacteria    p__Firmicutes  c__Clostridia o__Clostridiales
## denovo254 k__Bacteria    p__Firmicutes  c__Clostridia o__Clostridiales
## denovo170 k__Bacteria p__Bacteroidetes c__Bacteroidia o__Bacteroidales
##                          Family              Genus       Species
## denovo221     f__Prevotellaceae       g__Prevotella           s__
## denovo114     f__Bacteroidaceae      g__Bacteroides s__coprophilus
## denovo106    f__Ruminococcaceae     g__Ruminococcus           s__
## denovo281     f__Bacteroidaceae      g__Bacteroides           s__
## denovo91  f__Porphyromonadaceae g__Parabacteroides  s__distasonis
## denovo268      f__Rikenellaceae                 g__           s__
## denovo958                   f__                 g__           s__
## denovo110    f__Ruminococcaceae                 g__           s__
## denovo254                   f__                 g__           s__
## denovo170              f__S24-7                 g__           s__
##                 logFC   logCPM        LR    PValue       FDR
## denovo221  0.24766861 17.18822 1.2798284 0.2579309 0.9905029
## denovo114 -0.21934290 16.67888 1.0259492 0.3111120 0.9905029
## denovo106  0.21715616 16.01884 0.8628232 0.3529498 0.9905029
## denovo281 -0.17368079 16.36366 0.7054190 0.4009688 0.9905029
## denovo91   0.20900336 19.64047 0.4923223 0.4828932 0.9905029
## denovo268  0.16098626 16.59451 0.4821968 0.4874289 0.9905029
## denovo958  0.10506717 14.22086 0.2692871 0.6038104 0.9905029
## denovo110  0.15817742 17.94678 0.2309763 0.6308009 0.9905029
## denovo254 -0.10783170 15.25765 0.2270809 0.6336968 0.9905029
## denovo170  0.08645575 15.40426 0.1956921 0.6582207 0.9905029
```

Packages used

```r
citation("edgeR")
```

```
##
## See Section 1.2 in the User's Guide for more detail about how to
## cite the different edgeR pipelines.
##
##   Robinson MD, McCarthy DJ and Smyth GK (2010). edgeR: a
##   Bioconductor package for differential expression analysis of
##   digital gene expression data. Bioinformatics 26, 139-140
##
##   McCarthy DJ, Chen Y and Smyth GK (2012). Differential expression
##   analysis of multifactor RNA-Seq experiments with respect to
##   biological variation. Nucleic Acids Research 40, 4288-4297
##
## To see these entries in BibTeX format, use 'print(<citation>,
## bibtex=TRUE)', 'toBibtex(.)', or set
## 'options(citation.bibtex.max=999)'.
```

```r
citation("phyloseq")
```

```
##
## To cite phyloseq in publications, or otherwise credit, please use:
##
##   phyloseq: An R package for reproducible interactive analysis and
##   graphics of microbiome census data. Paul J. McMurdie and Susan
##   Holmes (2013) PLoS ONE 8(4):e61217.
##
## A BibTeX entry for LaTeX users is
##
##   @Article{,
##     author = {Paul J. McMurdie and Susan Holmes},
##     journal = {PLoS ONE},
##     pages = {e61217},
##    title = {phyloseq: An R package for reproducible interactive analysis and graphics of m
##     volume = {8},
##     number = {4},
##     year = {2013},
##     url = {http://dx.plos.org/10.1371/journal.pone.0061217},
##   }
```

```r
citation("ape")
```

```
##
```

```
## To cite ape in a publication use:
##
##   Paradis E. & Schliep K. 2018. ape 5.0: an environment for modern
##   phylogenetics and evolutionary analyses in R. Bioinformatics xx:
##   xxx-xxx.
##
## A BibTeX entry for LaTeX users is
##
##   @Article{,
##    title = {ape 5.0: an environment for modern phylogenetics and evolutionary analyses in
##     author = {E. Paradis and K. Schliep},
##     journal = {Bioinformatics},
##     year = {2018},
##     volume = {xx},
##     pages = {xxx-xxx},
##   }
##
## As ape is evolving quickly, you may want to cite also its version
## number (found with 'library(help = ape)' or
## 'packageVersion("ape")').
```

```
citation("data.table")
```

```
##
## To cite package 'data.table' in publications use:
##
##   Matt Dowle and Arun Srinivasan (2018). data.table: Extension of
##   `data.frame`. R package version 1.11.8.
##   https://CRAN.R-project.org/package=data.table
##
## A BibTeX entry for LaTeX users is
##
##   @Manual{,
##     title = {data.table: Extension of `data.frame`},
##     author = {Matt Dowle and Arun Srinivasan},
##     year = {2018},
##     note = {R package version 1.11.8},
##     url = {https://CRAN.R-project.org/package=data.table},
##   }
```

```
citation("knitr")
```

```
##
## To cite the 'knitr' package in publications use:
##
##   Yihui Xie (2018). knitr: A General-Purpose Package for Dynamic
```

```
##   Report Generation in R. R package version 1.20.
##
##   Yihui Xie (2015) Dynamic Documents with R and knitr. 2nd
##   edition. Chapman and Hall/CRC. ISBN 978-1498716963
##
##   Yihui Xie (2014) knitr: A Comprehensive Tool for Reproducible
##   Research in R. In Victoria Stodden, Friedrich Leisch and Roger
##   D. Peng, editors, Implementing Reproducible Computational
##   Research. Chapman and Hall/CRC. ISBN 978-1466561595
##
## To see these entries in BibTeX format, use 'print(<citation>,
## bibtex=TRUE)', 'toBibtex(.)', or set
## 'options(citation.bibtex.max=999)'.
```

```
citation("rmarkdown")
```

```
##
## To cite package 'rmarkdown' in publications use:
##
##   JJ Allaire, Yihui Xie, Jonathan McPherson, Javier Luraschi,
##   Kevin Ushey, Aron Atkins, Hadley Wickham, Joe Cheng and Winston
##   Chang (2018). rmarkdown: Dynamic Documents for R. R package
##   version 1.10. https://CRAN.R-project.org/package=rmarkdown
##
## A BibTeX entry for LaTeX users is
##
##   @Manual{,
##     title = {rmarkdown: Dynamic Documents for R},
##    author = {JJ Allaire and Yihui Xie and Jonathan McPherson and Javier Luraschi and Kevin
##     year = {2018},
##     note = {R package version 1.10},
##     url = {https://CRAN.R-project.org/package=rmarkdown},
##   }
```

```
citation("base")
```

```
##
## To cite R in publications use:
##
##   R Core Team (2018). R: A language and environment for
##   statistical computing. R Foundation for Statistical Computing,
##   Vienna, Austria. URL https://www.R-project.org/.
##
## A BibTeX entry for LaTeX users is
##
##   @Manual{,
```

```
##     title = {R: A Language and Environment for Statistical Computing},
##     author = {{R Core Team}},
##     organization = {R Foundation for Statistical Computing},
##     address = {Vienna, Austria},
##     year = {2018},
##     url = {https://www.R-project.org/},
##   }
##
## We have invested a lot of time and effort in creating R, please
## cite it when using it for data analysis. See also
## 'citation("pkgname")' for citing R packages.
```
