# Supplementary material for: Socioeconomic Status and the Gut Microbiome: A TwinsUK Cohort Study
Source: Microorganisms. 2019 Jan 11;7(1):17. doi: 10.3390/microorganisms7010017 (PMC6351927; doi:10.3390/microorganisms7010017)
Supplement: Supplementary file 1 [file microorganisms-07-00017-s001.zip › Additional_file_1_supptables.docx]

**Additional File 1: Supplementary tables**

**Supplementary table 1.** Thresholds for a) Body Mass Index (BMI) and b) health deficit (FI) and number of participants in each category.

**a.**

|  | Underweight (BMI < 20) | Healthy weight  (20 ≤ BMI < 25) | Overweight  (25 ≤ BMI < 30) | Obese  (BMI ≥ 30 ) |
| --- | --- | --- | --- | --- |
| n | 116 | 688 | 578 | 290 |

**b.**

|  | Healthy  (FI < 0.12) | Pre-frail  (0.12 ≤ FI < 0.3) | Frail  (0.3 ≤ FI < 0.44) | Very Frail  (FI ≥ 0.44) |
| --- | --- | --- | --- | --- |
| n | 557 | 829 | 208 | 78 |

**Supplementary table 2. Alpha Diversity and Socioeconomic Status**

|  | **i. Covariates Only** | | | **ii. IMD, Crude** | | | **iii. IMD, adjusted** | | | **iv. Income Crude** | | | **v. Income, adjusted** | | |
| --- | --- | --- | --- | --- | --- | --- | --- | --- | --- | --- | --- | --- | --- | --- | --- |
|  | **Chao1** | **Shannon** | **Simpson** | **Chao1** | **Shannon** | **Simpson** | **Chao1** | **Shannon** | **Simpson** | **Chao1** | **Shannon** | **Simpson** | **Chao1** | **Shannon** | **Simpson** |
| ***AIC*** | 3329.1 | 4662.7 | 4732.9 | 3323.9 | 4751.0 | 4761.2 | 3333.761 | 4665.714 | 4738.234 | 1505.0 | 2319.7 | 2401.0 | 1514.7 | 2294.3 | 2406.2 |
| IMD.2 | - | - | - | -0.01 | **0.1***** | **0.07**** | -0.01 | **0.07**** | 0.05 | - | - | - | - | - | - |
| IMD.3 | - | - | - | 0.02 | 0.04 | 0.04 | 0.02 | 0.03 | 0.03 | - | - | - | - | - | - |
| IMD.4 | - | - | - | 0.02 | **0.05*** | 0.05 | 0.02 | 0.02 | 0.03 | - | - | - | - | - | - |
| IMD.5 | - | - | - | 0.02 | **0.07**** | 0.04 | 0.02 | 0.03 | 0.02 | - | - | - | - | - | - |
| Income.2 | - | - | - | - | - | - | - | - | - | 0.02 | 0.03 | 0.06 | 0.02 | 0.01 | 0.05 |
| Income.3 | - | - | - | - | - | - | - | - | - | **0.07**** | **0.1**** | **0.13**** | **0.07**** | 0.08 | **0.11**** |
| Income.4 | - | - | - | - | - | - | - | - | - | **0.05*** | **0.14***** | **0.14***** | **0.05*** | **0.09*** | **0.12**** |
| Age | 0 | **0.13****** | **0.08***** | - | - | - | 0 | **0.13****** | **0.07***** | - | - | - | 0 | **0.08**** | 0.03 |
| BMI | 0 | **-0.11****** | **-0.06**** | - | - | - | 0 | **-0.1****** | **-0.06**** | - | - | - | 0.01 | **-0.06*** | -0.02 |
| FI | -0.02 | **-0.19****** | **-0.11****** | - | - | - | -0.02 | **-0.18****** | **-0.11****** | - | - | - | -0.02 | **-0.21****** | **-0.1**** |
| HEI | -0.02 | **0.08****** | **0.06**** | - | - | - | -0.02 | **0.08***** | **0.05**** | - | - | - | -0.03 | **0.07*** | 0.03 |

**Standardised coefficients and AIC values of hierarchical linear mixed effects models of alpha diversity (Chao1, Shannon diversity index and Simpson's diversity index): i. Covariate model, where model variables were Age, Body Mass index (BMI - kg/m^2^), health deficit (FI) and diet (HEI); ii) Crude income model iii) Adjusted Income model iv) Crude IMD model and v) IMD adjusted model. All models were adjusted for technical covariates modelled as random effects. Education models are not included due to non-significance. P-values in bold where * <0.1, **<0.05, ***<0.01, ****<0.001.**

**Supplementary table 3.** Differential abundance of OTUs by lowest to highest education group. Results are where q < 0.01

| **id** | **baseMean** | **log2FoldChange** | **lfcSE** | **stat** | **pvalue** | **padj** | **Kingdom** | **Phylum** | **Class** | **Order** | **Family** | **Genus** | **Species** |
| --- | --- | --- | --- | --- | --- | --- | --- | --- | --- | --- | --- | --- | --- |
| denovo281 | 12.60329 | 4.394263 | 0.452523 | 9.710593 | 2.72E-22 | 2.86E-19 | k__Bacteria | p__Bacteroidetes | c__Bacteroidia | o__Bacteroidales | f__Bacteroidaceae | g__Bacteroides | s__ |
| denovo167 | 29.60776 | 5.349413 | 0.611631 | 8.746149 | 2.21E-18 | 1.16E-15 | k__Bacteria | p__Bacteroidetes | c__Bacteroidia | o__Bacteroidales | f__S24-7 | g__ | s__ |
| denovo165 | 34.89897 | -1.27889 | 0.180668 | -7.0787 | 1.46E-12 | 5.11E-10 | k__Bacteria | p__Firmicutes | c__Clostridia | o__Clostridiales | f__Lachnospiraceae | g__ | s__ |
| denovo40 | 197.0657 | 2.23438 | 0.332062 | 6.7288 | 1.71E-11 | 4.51E-09 | k__Bacteria | p__Bacteroidetes | c__Bacteroidia | o__Bacteroidales | f__Bacteroidaceae | g__Bacteroides | s__ |
| denovo308 | 6.827544 | 4.910279 | 0.839734 | 5.847419 | 4.99E-09 | 8.77E-07 | k__Bacteria | p__Bacteroidetes | c__Bacteroidia | o__Bacteroidales | f__Bacteroidaceae | g__Bacteroides | s__ |
| denovo469 | 49.09564 | 1.258105 | 0.214341 | 5.869632 | 4.37E-09 | 8.77E-07 | k__Bacteria | p__Firmicutes | c__Clostridia | o__Clostridiales | f__Ruminococcaceae | g__Faecalibacterium | s__prausnitzii |
| denovo4 | 1431.755 | -1.42639 | 0.245646 | -5.8067 | 6.37E-09 | 9.59E-07 | k__Bacteria | p__Proteobacteria | c__Gammaproteobacteria | o__Enterobacteriales | f__Enterobacteriaceae | g__ | s__ |
| denovo77 | 31.55063 | 3.002451 | 0.528047 | 5.685958 | 1.30E-08 | 1.71E-06 | k__Bacteria | p__Bacteroidetes | c__Bacteroidia | o__Bacteroidales | f__Prevotellaceae | g__Prevotella | s__ |
| denovo254 | 7.449465 | 1.848764 | 0.331094 | 5.583807 | 2.35E-08 | 2.76E-06 | k__Bacteria | p__Firmicutes | c__Clostridia | o__Clostridiales | f__ | g__ | s__ |
| denovo88 | 106.059 | -0.8351 | 0.152146 | -5.48878 | 4.05E-08 | 4.27E-06 | k__Bacteria | p__Firmicutes | c__Clostridia | o__Clostridiales | f__Ruminococcaceae | g__ | s__ |
| denovo484 | 2.119049 | -2.51153 | 0.482275 | -5.20767 | 1.91E-07 | 1.83E-05 | k__Bacteria | p__Firmicutes | c__Clostridia | o__Clostridiales | f__Lachnospiraceae | g__Blautia | s__producta |
| denovo607 | 4.161361 | 3.393013 | 0.65447 | 5.18437 | 2.17E-07 | 1.90E-05 | k__Bacteria | p__Firmicutes | c__Clostridia | o__Clostridiales | f__Clostridiaceae | g__Clostridium | s__ |
| denovo632 | 1.369643 | -1.72091 | 0.346882 | -4.96108 | 7.01E-07 | 5.68E-05 | k__Bacteria | p__Firmicutes | c__Bacilli | o__Lactobacillales | f__Streptococcaceae | g__Streptococcus | s__anginosus |
| denovo604 | 5.167559 | 1.147988 | 0.233363 | 4.919329 | 8.68E-07 | 6.54E-05 | k__Bacteria | p__Firmicutes | c__Clostridia | o__Clostridiales | f__ | g__ | s__ |
| denovo278 | 6.570248 | -1.23541 | 0.25321 | -4.87898 | 1.07E-06 | 7.49E-05 | k__Bacteria | p__Firmicutes | c__Erysipelotrichi | o__Erysipelotrichales | f__Erysipelotrichaceae | g__[Eubacterium] | s__dolichum |
| denovo475 | 1.724149 | 3.023953 | 0.651481 | 4.641658 | 3.46E-06 | 0.000228 | k__Bacteria | p__Firmicutes | c__Clostridia | o__Clostridiales | f__ | g__ | s__ |
| denovo290 | 16.51381 | -1.30223 | 0.28188 | -4.61979 | 3.84E-06 | 0.000238 | k__Bacteria | p__Firmicutes | c__Clostridia | o__Clostridiales | f__Ruminococcaceae | g__ | s__ |
| denovo522 | 37.25966 | 0.756692 | 0.165338 | 4.576648 | 4.72E-06 | 0.000277 | k__Bacteria | p__Bacteroidetes | c__Bacteroidia | o__Bacteroidales | f__Bacteroidaceae | g__Bacteroides | s__ |
| denovo172 | 1.907728 | -3.7558 | 0.825556 | -4.54943 | 5.38E-06 | 0.000291 | k__Bacteria | p__Tenericutes | c__Mollicutes | o__RF39 | f__ | g__ | s__ |
| denovo184 | 3.401522 | 4.037535 | 0.888628 | 4.543559 | 5.53E-06 | 0.000291 | k__Bacteria | p__Proteobacteria | c__Deltaproteobacteria | o__Desulfovibrionales | f__Desulfovibrionaceae | g__Desulfovibrio | s__ |
| denovo272 | 25.16802 | -0.93205 | 0.205795 | -4.52901 | 5.93E-06 | 0.000295 | k__Bacteria | p__Firmicutes | c__Clostridia | o__Clostridiales | f__Lachnospiraceae | g__ | s__ |
| denovo461 | 2.279544 | -1.7437 | 0.385687 | -4.52103 | 6.15E-06 | 0.000295 | k__Bacteria | p__Firmicutes | c__Bacilli | o__Lactobacillales | f__Lactobacillaceae | g__Lactobacillus | s__zeae |
| denovo188 | 3.856902 | 2.614264 | 0.584001 | 4.476469 | 7.59E-06 | 0.000348 | k__Bacteria | p__Firmicutes | c__Clostridia | o__Clostridiales | f__Lachnospiraceae | g__Anaerostipes | s__ |
| denovo181 | 1.301482 | 4.145442 | 0.940983 | 4.405437 | 1.06E-05 | 0.000445 | k__Bacteria | p__Bacteroidetes | c__Bacteroidia | o__Bacteroidales | f__S24-7 | g__ | s__ |
| denovo8 | 2751.815 | 1.382126 | 0.313716 | 4.405667 | 1.05E-05 | 0.000445 | k__Bacteria | p__Bacteroidetes | c__Bacteroidia | o__Bacteroidales | f__Prevotellaceae | g__Prevotella | s__copri |
| denovo61 | 67.57997 | -1.55219 | 0.353126 | -4.39556 | 1.10E-05 | 0.000448 | k__Bacteria | p__Proteobacteria | c__Gammaproteobacteria | o__Enterobacteriales | f__Enterobacteriaceae | g__ | s__ |
| denovo186 | 4.304665 | -2.64002 | 0.608837 | -4.33617 | 1.45E-05 | 0.000566 | k__Bacteria | p__Firmicutes | c__Clostridia | o__Clostridiales | f__Christensenellaceae | g__ | s__ |
| denovo114 | 31.91715 | 2.293191 | 0.550023 | 4.169263 | 3.06E-05 | 0.001117 | k__Bacteria | p__Bacteroidetes | c__Bacteroidia | o__Bacteroidales | f__Bacteroidaceae | g__Bacteroides | s__coprophilus |
| denovo173 | 13.38568 | 2.401121 | 0.576808 | 4.16277 | 3.14E-05 | 0.001117 | k__Bacteria | p__Tenericutes | c__Mollicutes | o__RF39 | f__ | g__ | s__ |
| denovo826 | 1.300261 | -0.96526 | 0.232021 | -4.16023 | 3.18E-05 | 0.001117 | k__Bacteria | p__Firmicutes | c__Clostridia | o__Clostridiales | f__Eubacteriaceae | g__Anaerofustis | s__ |
| denovo55 | 347.4023 | -0.56158 | 0.135538 | -4.14335 | 3.42E-05 | 0.001164 | k__Bacteria | p__Firmicutes | c__Clostridia | o__Clostridiales | f__Lachnospiraceae | g__[Ruminococcus] | s__ |
| denovo273 | 15.26093 | 1.180237 | 0.29041 | 4.064033 | 4.82E-05 | 0.001589 | k__Bacteria | p__Bacteroidetes | c__Bacteroidia | o__Bacteroidales | f__[Barnesiellaceae] | g__ | s__ |
| denovo133 | 108.3421 | -0.89381 | 0.223976 | -3.99066 | 6.59E-05 | 0.002105 | k__Bacteria | p__Firmicutes | c__Clostridia | o__Clostridiales | f__Veillonellaceae | g__Veillonella | s__dispar |
| denovo313 | 23.3499 | 0.813174 | 0.204411 | 3.978132 | 6.95E-05 | 0.002153 | k__Bacteria | p__Firmicutes | c__Clostridia | o__Clostridiales | f__Lachnospiraceae | g__ | s__ |
| denovo218 | 45.53467 | 0.783536 | 0.199068 | 3.936017 | 8.28E-05 | 0.002495 | k__Bacteria | p__Firmicutes | c__Clostridia | o__Clostridiales | f__Lachnospiraceae | g__ | s__ |
| denovo147 | 29.64886 | 2.143399 | 0.545698 | 3.927812 | 8.57E-05 | 0.00251 | k__Bacteria | p__Tenericutes | c__Mollicutes | o__RF39 | f__ | g__ | s__ |
| denovo189 | 28.94727 | -1.00517 | 0.258625 | -3.88658 | 0.000102 | 0.002885 | k__Bacteria | p__Firmicutes | c__Clostridia | o__Clostridiales | f__ | g__ | s__ |
| denovo753 | 10.70776 | -1.21216 | 0.312327 | -3.88105 | 0.000104 | 0.002885 | k__Bacteria | p__Firmicutes | c__Clostridia | o__Clostridiales | f__Lachnospiraceae | g__ | s__ |
| denovo751 | 1.26949 | 1.856076 | 0.484992 | 3.827021 | 0.00013 | 0.003505 | k__Bacteria | p__Firmicutes | c__Clostridia | o__Clostridiales | f__Ruminococcaceae | g__ | s__ |
| denovo214 | 17.93042 | -1.16926 | 0.307485 | -3.80267 | 0.000143 | 0.003772 | k__Bacteria | p__Cyanobacteria | c__Chloroplast | o__Streptophyta | f__ | g__ | s__ |
| denovo1421 | 0.541301 | 1.415045 | 0.372782 | 3.795908 | 0.000147 | 0.003782 | k__Bacteria | p__Firmicutes | c__Clostridia | o__Clostridiales | f__Lachnospiraceae | g__Lachnospira | s__ |
| denovo127 | 28.61453 | -1.63781 | 0.433321 | -3.77966 | 0.000157 | 0.003941 | k__Bacteria | p__Firmicutes | c__Clostridia | o__Clostridiales | f__Christensenellaceae | g__ | s__ |
| denovo149 | 15.25898 | 2.33739 | 0.625197 | 3.738646 | 0.000185 | 0.004535 | k__Bacteria | p__Firmicutes | c__Clostridia | o__Clostridiales | f__Ruminococcaceae | g__ | s__ |
| denovo26 | 911.9465 | 0.5646 | 0.151713 | 3.721501 | 0.000198 | 0.004744 | k__Bacteria | p__Firmicutes | c__Clostridia | o__Clostridiales | f__Lachnospiraceae | g__ | s__ |
| denovo32 | 432.5442 | -0.70767 | 0.194894 | -3.63105 | 0.000282 | 0.006468 | k__Bacteria | p__Firmicutes | c__Clostridia | o__Clostridiales | f__Clostridiaceae | g__ | s__ |
| denovo495 | 4.282656 | -1.03954 | 0.28591 | -3.63589 | 0.000277 | 0.006468 | k__Bacteria | p__Firmicutes | c__Clostridia | o__Clostridiales | f__Lachnospiraceae | g__ | s__ |
| denovo76 | 108.8393 | 1.435907 | 0.400325 | 3.586855 | 0.000335 | 0.007506 | k__Bacteria | p__Firmicutes | c__Clostridia | o__Clostridiales | f__Ruminococcaceae | g__ | s__ |
| denovo543 | 1.445892 | -2.87907 | 0.806498 | -3.56984 | 0.000357 | 0.007844 | k__Bacteria | p__Firmicutes | c__Erysipelotrichi | o__Erysipelotrichales | f__Erysipelotrichaceae | g__[Eubacterium] | s__dolichum |
| denovo439 | 1.864798 | 3.866067 | 1.088339 | 3.552263 | 0.000382 | 0.008215 | k__Bacteria | p__Bacteroidetes | c__Bacteroidia | o__Bacteroidales | f__Rikenellaceae | g__ | s__ |
| denovo111 | 70.96254 | -0.50988 | 0.143934 | -3.54248 | 0.000396 | 0.008286 | k__Bacteria | p__Firmicutes | c__Clostridia | o__Clostridiales | f__Ruminococcaceae | g__Ruminococcus | s__ |
| denovo430 | 37.28582 | 0.927397 | 0.262016 | 3.539472 | 0.000401 | 0.008286 | k__Bacteria | p__Firmicutes | c__Clostridia | o__Clostridiales | f__Clostridiaceae | g__ | s__ |
| denovo1392 | 1.931791 | -1.80789 | 0.515587 | -3.50648 | 0.000454 | 0.009204 | k__Bacteria | p__Proteobacteria | c__Gammaproteobacteria | o__Enterobacteriales | f__Enterobacteriaceae | g__ | s__ |
| denovo104 | 70.10579 | -1.60818 | 0.460618 | -3.49135 | 0.000481 | 0.009545 | k__Bacteria | p__Firmicutes | c__Clostridia | o__Clostridiales | f__Ruminococcaceae | g__ | s__ |
| denovo361 | 22.571 | -0.62495 | 0.179399 | -3.48359 | 0.000495 | 0.009545 | k__Bacteria | p__Firmicutes | c__Bacilli | o__Lactobacillales | f__Streptococcaceae | g__Streptococcus | s__ |
| denovo467 | 5.064546 | -0.55888 | 0.160517 | -3.48178 | 0.000498 | 0.009545 | k__Bacteria | p__Actinobacteria | c__Actinobacteria | o__Actinomycetales | f__Actinomycetaceae | g__Actinomyces | s__ |
| denovo1800 | 1.783306 | -1.12152 | 0.323578 | -3.46601 | 0.000528 | 0.009768 | k__Bacteria | p__Firmicutes | c__Clostridia | o__Clostridiales | f__Lachnospiraceae | g__ | s__ |
| denovo487 | 5.397614 | -0.81933 | 0.236213 | -3.46862 | 0.000523 | 0.009768 | k__Bacteria | p__Firmicutes | c__Clostridia | o__Clostridiales | f__ | g__ | s__ |

**Supplementary table 4.** Differential abundance of OTUs by lowest to highest education group, in models adjusted for age, BMI, diet and health deficit. Results are where q < 0.01

| **Denovo id** | **baseMean** | **log2FoldChange** | **lfcSE** | **stat** | **pvalue** | **padj** | **Kingdom** | **Phylum** | **Class** | **Order** | **Family** | **Genus** | **Species** |
| --- | --- | --- | --- | --- | --- | --- | --- | --- | --- | --- | --- | --- | --- |
| denovo1034 | 3.387656 | -4.8889 | 0.644806 | -7.58196 | 3.40E-14 | 2.36E-11 | k__Bacteria | p__Firmicutes | c__Clostridia | o__Clostridiales | f__Lachnospiraceae | g__Blautia | s__producta |
| denovo1398 | 1.748111 | 1.047361 | 0.267577 | 3.914242 | 9.07E-05 | 0.007845 | k__Bacteria | p__Firmicutes | c__Clostridia | o__Clostridiales | f__Lachnospiraceae | g__Blautia | s__ |
| denovo144 | 99.05766 | -0.74814 | 0.168432 | -4.4418 | 8.92E-06 | 0.001378 | k__Bacteria | p__Firmicutes | c__Clostridia | o__Clostridiales | f__Lachnospiraceae | g__Blautia | s__ |
| denovo1604 | 0.898461 | -2.30401 | 0.532711 | -4.32507 | 1.52E-05 | 0.00211 | k__Bacteria | p__Firmicutes | c__Clostridia | o__Clostridiales | f__ | g__ | s__ |
| denovo189 | 32.52755 | -1.19383 | 0.284161 | -4.20124 | 2.65E-05 | 0.00334 | k__Bacteria | p__Firmicutes | c__Clostridia | o__Clostridiales | f__ | g__ | s__ |
| denovo239 | 18.92938 | -0.78211 | 0.190025 | -4.11581 | 3.86E-05 | 0.00445 | k__Bacteria | p__Firmicutes | c__Clostridia | o__Clostridiales | f__Ruminococcaceae | g__Ruminococcus | s__ |
| denovo277 | 29.29296 | 1.202673 | 0.303491 | 3.962802 | 7.41E-05 | 0.007323 | k__Bacteria | p__Firmicutes | c__Clostridia | o__Clostridiales | f__ | g__ | s__ |
| denovo313 | 31.81235 | 1.817319 | 0.232594 | 7.813254 | 5.57E-15 | 7.71E-12 | k__Bacteria | p__Firmicutes | c__Clostridia | o__Clostridiales | f__Lachnospiraceae | g__ | s__ |
| denovo469 | 49.09564 | 1.1111 | 0.231792 | 4.793515 | 1.64E-06 | 0.000646 | k__Bacteria | p__Firmicutes | c__Clostridia | o__Clostridiales | f__Ruminococcaceae | g__Faecalibacterium | s__prausnitzii |
| denovo487 | 5.397614 | -1.01972 | 0.255466 | -3.9916 | 6.56E-05 | 0.006987 | k__Bacteria | p__Firmicutes | c__Clostridia | o__Clostridiales | f__ | g__ | s__ |
| denovo522 | 37.25966 | 0.796479 | 0.179352 | 4.440869 | 8.96E-06 | 0.001378 | k__Bacteria | p__Bacteroidetes | c__Bacteroidia | o__Bacteroidales | f__Bacteroidaceae | g__Bacteroides | s__ |
| denovo604 | 5.432027 | 1.158701 | 0.252314 | 4.592303 | 4.38E-06 | 0.001002 | k__Bacteria | p__Firmicutes | c__Clostridia | o__Clostridiales | f__ | g__ | s__ |
| denovo751 | 1.392922 | 2.491469 | 0.522614 | 4.767319 | 1.87E-06 | 0.000646 | k__Bacteria | p__Firmicutes | c__Clostridia | o__Clostridiales | f__Ruminococcaceae | g__ | s__ |
| denovo826 | 1.300261 | -0.9852 | 0.250338 | -3.93546 | 8.30E-05 | 0.007661 | k__Bacteria | p__Firmicutes | c__Clostridia | o__Clostridiales | f__Eubacteriaceae | g__Anaerofustis | s__ |
| denovo831 | 3.590916 | 3.052828 | 0.794386 | 3.843005 | 0.000122 | 0.009895 | k__Bacteria | p__Bacteroidetes | c__Bacteroidia | o__Bacteroidales | f__Prevotellaceae | g__Prevotella | s__copri |
| denovo832 | 0.981605 | 2.883067 | 0.619733 | 4.65211 | 3.29E-06 | 0.000909 | k__Bacteria | p__Bacteroidetes | c__Bacteroidia | o__Bacteroidales | f__Porphyromonadaceae | g__Porphyromonas | s__ |
| denovo88 | 106.059 | -0.74401 | 0.163088 | -4.56203 | 5.07E-06 | 0.001002 | k__Bacteria | p__Firmicutes | c__Clostridia | o__Clostridiales | f__Ruminococcaceae | g__ | s__ |

**Supplementary table 5.** Differential abundance of OTUs by lowest to highest Index of Multiple Deprivation (IMD) quantile. Results are where q < 0.01

|  | **baseMean** | **log2FoldChange** | **lfcSE** | **stat** | **pvalue** | **padj** | **Kingdom** | **Phylum** | **Class** | **Order** | **Family** | **Genus** | **Species** |
| --- | --- | --- | --- | --- | --- | --- | --- | --- | --- | --- | --- | --- | --- |
| denovo165 | 34.39125 | -1.16877 | 0.168701 | -6.92805 | 4.27E-12 | 4.50E-09 | k__Bacteria | p__Firmicutes | c__Clostridia | o__Clostridiales | f__Lachnospiraceae | g__ | s__ |
| denovo170 | 8.157177 | -5.56559 | 0.90781 | -6.13079 | 8.74E-10 | 4.61E-07 | k__Bacteria | p__Bacteroidetes | c__Bacteroidia | o__Bacteroidales | f__S24-7 | g__ | s__ |
| denovo268 | 7.599738 | 3.194554 | 0.553979 | 5.766562 | 8.09E-09 | 2.84E-06 | k__Bacteria | p__Bacteroidetes | c__Bacteroidia | o__Bacteroidales | f__Rikenellaceae | g__ | s__ |
| denovo114 | 30.69891 | -2.81027 | 0.517072 | -5.43497 | 5.48E-08 | 1.16E-05 | k__Bacteria | p__Bacteroidetes | c__Bacteroidia | o__Bacteroidales | f__Bacteroidaceae | g__Bacteroides | s__coprophilus |
| denovo254 | 6.521487 | 1.694125 | 0.31048 | 5.456479 | 4.86E-08 | 1.16E-05 | k__Bacteria | p__Firmicutes | c__Clostridia | o__Clostridiales | f__ | g__ | s__ |
| denovo389 | 3.428106 | 2.500929 | 0.484633 | 5.160461 | 2.46E-07 | 4.33E-05 | k__Bacteria | p__Firmicutes | c__Clostridia | o__Clostridiales | f__ | g__ | s__ |
| denovo281 | 8.560837 | -2.12123 | 0.417416 | -5.08182 | 3.74E-07 | 5.63E-05 | k__Bacteria | p__Bacteroidetes | c__Bacteroidia | o__Bacteroidales | f__Bacteroidaceae | g__Bacteroides | s__ |
| denovo724 | 1.312991 | -1.48453 | 0.295293 | -5.02731 | 4.97E-07 | 6.55E-05 | k__Bacteria | p__Bacteroidetes | c__Bacteroidia | o__Bacteroidales | f__Bacteroidaceae | g__Bacteroides | s__ |
| denovo288 | 15.69858 | -0.90397 | 0.193659 | -4.66785 | 3.04E-06 | 0.000356 | k__Bacteria | p__Firmicutes | c__Clostridia | o__Clostridiales | f__Ruminococcaceae | g__ | s__ |
| denovo110 | 87.45841 | 1.159789 | 0.262497 | 4.418296 | 9.95E-06 | 0.001049 | k__Bacteria | p__Firmicutes | c__Clostridia | o__Clostridiales | f__Ruminococcaceae | g__ | s__ |
| denovo143 | 1.225768 | -3.1073 | 0.710263 | -4.37485 | 1.22E-05 | 0.001164 | k__Bacteria | p__Bacteroidetes | c__Bacteroidia | o__Bacteroidales | f__Prevotellaceae | g__Prevotella | s__ |
| denovo221 | 1.411816 | 4.523099 | 1.079671 | 4.18933 | 2.80E-05 | 0.002457 | k__Bacteria | p__Bacteroidetes | c__Bacteroidia | o__Bacteroidales | f__Prevotellaceae | g__Prevotella | s__ |
| denovo278 | 6.704264 | -0.95178 | 0.234678 | -4.05568 | 5.00E-05 | 0.004044 | k__Bacteria | p__Firmicutes | c__Erysipelotrichi | o__Erysipelotrichales | f__Erysipelotrichaceae | g__Eubacterium | s__dolichum |
| denovo161 | 26.40084 | -1.09747 | 0.271728 | -4.03887 | 5.37E-05 | 0.004044 | k__Bacteria | p__Firmicutes | c__Erysipelotrichi | o__Erysipelotrichales | f__Erysipelotrichaceae | g__ | s__ |
| denovo106 | 30.69939 | 1.923275 | 0.478358 | 4.020572 | 5.81E-05 | 0.004079 | k__Bacteria | p__Firmicutes | c__Clostridia | o__Clostridiales | f__Ruminococcaceae | g__Ruminococcus | s__ |
| denovo91 | 343.9092 | -0.67474 | 0.173436 | -3.89045 | 0.0001 | 0.006591 | k__Bacteria | p__Bacteroidetes | c__Bacteroidia | o__Bacteroidales | f__Porphyromonadaceae | g__Parabacteroides | s__distasonis |
| denovo83 | 54.41056 | -2.07279 | 0.548651 | -3.77798 | 0.000158 | 0.009731 | k__Bacteria | p__Firmicutes | c__Erysipelotrichi | o__Erysipelotrichales | f__Erysipelotrichaceae | g__Catenibacterium | s__ |
| denovo958 | 6.06011 | 0.910571 | 0.241816 | 3.765549 | 0.000166 | 0.009731 | k__Bacteria | p__Firmicutes | c__Clostridia | o__Clostridiales | f__ | g__ | s__ |

**Supplementary table 6**. Differential abundance of OTUs by lowest to highest Index of Multiple Deprivation (IMD) quantile, in models adjusted for age, BMI, diet and health deficit. Results are where q < 0.01

|  | baseMean | log2FoldChange | lfcSE | stat | pvalue | padj | Kingdom | Phylum | Class | Order | Family | Genus | Species |
| --- | --- | --- | --- | --- | --- | --- | --- | --- | --- | --- | --- | --- | --- |
| denovo1192 | 1.1645 | -3.5656 | 0.5226 | -6.8232 | 0.0000 | 0.0000 | k__Bacteria | p__Firmicutes | c__Clostridia | o__Clostridiales | f__Ruminococcaceae | g__ | s__ |
| denovo724 | 2.6666 | -2.1427 | 0.3399 | -6.3044 | 0.0000 | 0.0000 | k__Bacteria | p__Bacteroidetes | c__Bacteroidia | o__Bacteroidales | f__Bacteroidaceae | g__Bacteroides | s__ |
| denovo144 | 99.0250 | -0.7971 | 0.1441 | -5.5312 | 0.0000 | 0.0000 | k__Bacteria | p__Firmicutes | c__Clostridia | o__Clostridiales | f__Lachnospiraceae | g__Blautia | s__ |
| denovo165 | 71.4831 | -0.9311 | 0.1935 | -4.8119 | 0.0000 | 0.0004 | k__Bacteria | p__Firmicutes | c__Clostridia | o__Clostridiales | f__Lachnospiraceae | g__ | s__ |
| denovo1224 | 2.2811 | 0.9456 | 0.2021 | 4.6783 | 0.0000 | 0.0006 | k__Bacteria | p__Firmicutes | c__Clostridia | o__Clostridiales | f__Lachnospiraceae | NA | NA |
| denovo735 | 0.8490 | -1.9799 | 0.4234 | -4.6765 | 0.0000 | 0.0006 | k__Bacteria | p__Firmicutes | c__Clostridia | o__Clostridiales | f__Tissierellaceae | g__Parvimonas | s__ |
| denovo110 | 87.4584 | 1.1759 | 0.2607 | 4.5106 | 0.0000 | 0.0011 | k__Bacteria | p__Firmicutes | c__Clostridia | o__Clostridiales | f__Ruminococcaceae | g__ | s__ |
| denovo1105 | 0.7808 | 1.6107 | 0.3598 | 4.4764 | 0.0000 | 0.0011 | k__Bacteria | p__Firmicutes | c__Clostridia | o__Clostridiales | f__Ruminococcaceae | g__Oscillospira | s__ |
| denovo689 | 13.8327 | 1.3576 | 0.3125 | 4.3440 | 0.0000 | 0.0018 | k__Bacteria | p__Bacteroidetes | c__Bacteroidia | o__Bacteroidales | f__Barnesiellaceae | g__ | s__ |
| denovo1034 | 3.0037 | -2.4361 | 0.5780 | -4.2143 | 0.0000 | 0.0026 | k__Bacteria | p__Firmicutes | c__Clostridia | o__Clostridiales | f__Lachnospiraceae | g__Blautia | s__producta |
| denovo91 | 366.1576 | -0.7440 | 0.1762 | -4.2232 | 0.0000 | 0.0026 | k__Bacteria | p__Bacteroidetes | c__Bacteroidia | o__Bacteroidales | f__Porphyromonadaceae | g__Parabacteroides | s__distasonis |
| denovo357 | 11.2919 | -1.3567 | 0.3244 | -4.1821 | 0.0000 | 0.0027 | k__Bacteria | p__Firmicutes | c__Clostridia | o__Clostridiales | f__ | g__ | s__ |
| denovo18001 | 0.6692 | 1.4389 | 0.3462 | 4.1558 | 0.0000 | 0.0028 | k__Bacteria | p__Bacteroidetes | c__Bacteroidia | o__Bacteroidales | f__Barnesiellaceae | g__ | s__ |
| denovo543 | 4.1395 | -3.2608 | 0.8049 | -4.0513 | 0.0001 | 0.0041 | k__Bacteria | p__Firmicutes | c__Erysipelotrichi | o__Erysipelotrichales | f__Erysipelotrichaceae | g__Eubacterium | s__dolichum |
| denovo18013 | 0.7458 | 1.2472 | 0.3250 | 3.8378 | 0.0001 | 0.0094 | k__Bacteria | p__Bacteroidetes | c__Bacteroidia | o__Bacteroidales | f__Barnesiellaceae | g__ | s__ |

**Supplementary table 7.** Differential abundance of OTUs by lowest to highest income group. Results are where q < 0.01

| **ID** | **baseMean** | **log2FoldChange** | **lfcSE** | **stat** | **pvalue** | **padj** | **Kingdom** | **Phylum** | **Class** | **Order** | **Family** | **Genus** | **Species** |
| --- | --- | --- | --- | --- | --- | --- | --- | --- | --- | --- | --- | --- | --- |
| denovo2 | 2627.824 | -1.90244 | 0.311225 | -6.11275 | 9.79E-10 | 3.17E-07 | k__Bacteria | p__Verrucomicrobia | c__Verrucomicrobiae | o__Verrucomicrobiales | f__Verrucomicrobiaceae | g__Akkermansia | s__muciniphila |
| denovo495 | 4.382719 | -2.46078 | 0.404257 | -6.08716 | 1.15E-09 | 3.17E-07 | k__Bacteria | p__Firmicutes | c__Clostridia | o__Clostridiales | f__Lachnospiraceae | g__ | s__ |
| denovo99 | 25.44404 | -3.95501 | 0.650161 | -6.08313 | 1.18E-09 | 3.17E-07 | k__Bacteria | p__Firmicutes | c__Clostridia | o__Clostridiales | f__Veillonellaceae | g__Acidaminococcus | s__ |
| denovo165 | 39.54423 | -1.61213 | 0.268556 | -6.00295 | 1.94E-09 | 3.91E-07 | k__Bacteria | p__Firmicutes | c__Clostridia | o__Clostridiales | f__Lachnospiraceae | g__ | s__ |
| denovo400 | 7.641997 | -2.19638 | 0.383542 | -5.72655 | 1.02E-08 | 1.65E-06 | k__Bacteria | p__Firmicutes | c__Erysipelotrichi | o__Erysipelotrichales | f__Erysipelotrichaceae | g__Coprobacillus | s__ |
| denovo77 | 10.34353 | 3.316338 | 0.684013 | 4.848357 | 1.24E-06 | 0.000167 | k__Bacteria | p__Bacteroidetes | c__Bacteroidia | o__Bacteroidales | f__Prevotellaceae | g__Prevotella | s__ |
| denovo1750 | 0.974622 | -2.52706 | 0.537815 | -4.69876 | 2.62E-06 | 0.000302 | k__Bacteria | p__Firmicutes | c__Clostridia | o__Clostridiales | f__Lachnospiraceae | g__[Ruminococcus] | s__gnavus |
| denovo469 | 46.45609 | 1.346794 | 0.298564 | 4.510903 | 6.46E-06 | 0.000651 | k__Bacteria | p__Firmicutes | c__Clostridia | o__Clostridiales | f__Ruminococcaceae | g__Faecalibacterium | s__prausnitzii |
| denovo60 | 241.5965 | -1.11445 | 0.259828 | -4.28919 | 1.79E-05 | 0.001608 | k__Bacteria | p__Firmicutes | c__Bacilli | o__Lactobacillales | f__Streptococcaceae | g__Streptococcus | s__ |
| denovo22 | 863.9209 | -1.02631 | 0.25142 | -4.08204 | 4.46E-05 | 0.003603 | k__Bacteria | p__Actinobacteria | c__Actinobacteria | o__Bifidobacteriales | f__Bifidobacteriaceae | g__Bifidobacterium | s__adolescentis |

**Supplementary table 8.** Differential abundance of OTUs by lowest to highest income group, in models adjusted for age, BMI, diet and health deficit. Results are where q < 0.01

| **Denovo id** | **baseMean** | **log2FoldChange** | **lfcSE** | **stat** | **pvalue** | **padj** | **Kingdom** | **Phylum** | **Class** | **Order** | **Family** | **Genus** | **Species** |
| --- | --- | --- | --- | --- | --- | --- | --- | --- | --- | --- | --- | --- | --- |
| denovo165 | 48.77729 | -1.99671 | 0.287854 | -6.93652 | 4.02E-12 | 4.90E-09 | k__Bacteria | p__Firmicutes | c__Clostridia | o__Clostridiales | f__Lachnospiraceae | g__ | s__ |
| denovo2 | 2627.824 | -1.87087 | 0.321282 | -5.82316 | 5.77E-09 | 3.52E-06 | k__Bacteria | p__Verrucomicrobia | c__Verrucomicrobiae | o__Verrucomicrobiales | f__Verrucomicrobiaceae | g__Akkermansia | s__muciniphila |
| denovo4 | 1534.201 | 1.569811 | 0.360856 | 4.350246 | 1.36E-05 | 0.005526 | k__Bacteria | p__Proteobacteria | c__Gammaproteobacteria | o__Enterobacteriales | f__Enterobacteriaceae | g__ | s__ |

**Supplementary table 9.** Differential abundance of OTUs by Health deficit (FI) as a factor. Results are where q < 0.01

| id | baseMean | log2FoldChange | lfcSE | stat | pvalue | padj | Kingdom | Phylum | Class | Order | Family | Genus | Species |
| --- | --- | --- | --- | --- | --- | --- | --- | --- | --- | --- | --- | --- | --- |
| denovo8 | 2140.35 | -3.45824 | 0.454264 | -7.61284 | 2.68E-14 | 2.61E-11 | k__Bacteria | p__Bacteroidetes | c__Bacteroidia | o__Bacteroidales | f__Prevotellaceae | g__Prevotella | s__copri |
| denovo114 | 33.72017 | -6.11658 | 0.837182 | -7.30615 | 2.75E-13 | 1.11E-10 | k__Bacteria | p__Bacteroidetes | c__Bacteroidia | o__Bacteroidales | f__Bacteroidaceae | g__Bacteroides | s__coprophilus |
| denovo93 | 58.9587 | -5.82192 | 0.800034 | -7.27708 | 3.41E-13 | 1.11E-10 | k__Bacteria | p__Tenericutes | c__Mollicutes | o__RF39 | f__ | g__ | s__ |
| denovo88 | 107.1464 | 1.558527 | 0.217228 | 7.174621 | 7.25E-13 | 1.76E-10 | k__Bacteria | p__Firmicutes | c__Clostridia | o__Clostridiales | f__Ruminococcaceae | g__ | s__ |
| denovo77 | 36.61969 | -5.7723 | 0.827621 | -6.97456 | 3.07E-12 | 5.96E-10 | k__Bacteria | p__Bacteroidetes | c__Bacteroidia | o__Bacteroidales | f__Prevotellaceae | g__Prevotella | s__ |
| denovo76 | 104.9048 | -3.81491 | 0.584647 | -6.52515 | 6.79E-11 | 9.91E-09 | k__Bacteria | p__Firmicutes | c__Clostridia | o__Clostridiales | f__Ruminococcaceae | g__ | s__ |
| denovo80 | 47.93211 | -4.61533 | 0.708125 | -6.51768 | 7.14E-11 | 9.91E-09 | k__Bacteria | p__Firmicutes | c__Clostridia | o__Clostridiales | f__ | g__ | s__ |
| denovo139 | 27.83845 | -5.8787 | 0.9373 | -6.27195 | 3.57E-10 | 4.33E-08 | k__Bacteria | p__Tenericutes | c__Mollicutes | o__RF39 | f__ | g__ | s__ |
| denovo136 | 54.77191 | -2.67343 | 0.433302 | -6.16989 | 6.83E-10 | 6.64E-08 | k__Bacteria | p__Firmicutes | c__Clostridia | o__Clostridiales | f__ | g__ | s__ |
| denovo281 | 12.59465 | -4.37552 | 0.709162 | -6.16999 | 6.83E-10 | 6.64E-08 | k__Bacteria | p__Bacteroidetes | c__Bacteroidia | o__Bacteroidales | f__Bacteroidaceae | g__Bacteroides | s__ |
| denovo180 | 34.84431 | -2.32985 | 0.386091 | -6.03446 | 1.59E-09 | 1.41E-07 | k__Bacteria | p__Firmicutes | c__Clostridia | o__Clostridiales | f__ | g__ | s__ |
| denovo218 | 46.85138 | -1.76453 | 0.296298 | -5.95526 | 2.60E-09 | 1.94E-07 | k__Bacteria | p__Firmicutes | c__Clostridia | o__Clostridiales | f__Lachnospiraceae | g__ | s__ |
| denovo85 | 45.02833 | -5.19361 | 0.871186 | -5.96154 | 2.50E-09 | 1.94E-07 | k__Bacteria | p__Tenericutes | c__Mollicutes | o__RF39 | f__ | g__ | s__ |
| denovo449 | 13.10573 | 1.556989 | 0.279117 | 5.578271 | 2.43E-08 | 1.69E-06 | k__Bacteria | p__Bacteroidetes | c__Bacteroidia | o__Bacteroidales | f__Bacteroidaceae | g__Bacteroides | s__ |
| denovo119 | 37.05173 | -4.65733 | 0.839809 | -5.54571 | 2.93E-08 | 1.78E-06 | k__Bacteria | p__Tenericutes | c__Mollicutes | o__RF39 | f__ | g__ | s__ |
| denovo150 | 27.29557 | -4.51268 | 0.813488 | -5.54732 | 2.90E-08 | 1.78E-06 | k__Bacteria | p__Firmicutes | c__Clostridia | o__Clostridiales | f__Ruminococcaceae | g__Ruminococcus | s__ |
| denovo153 | 37.736 | 2.040902 | 0.380107 | 5.369282 | 7.91E-08 | 4.52E-06 | k__Bacteria | p__Firmicutes | c__Clostridia | o__Clostridiales | f__Lachnospiraceae | g__Dorea | s__ |
| denovo109 | 96.23909 | -2.60657 | 0.489912 | -5.32049 | 1.03E-07 | 5.39E-06 | k__Bacteria | p__Firmicutes | c__Clostridia | o__Clostridiales | f__ | g__ | s__ |
| denovo25 | 828.1551 | 0.912687 | 0.171649 | 5.317181 | 1.05E-07 | 5.39E-06 | k__Bacteria | p__Firmicutes | c__Clostridia | o__Clostridiales | f__Lachnospiraceae | g__Blautia | s__ |
| denovo165 | 39.18622 | 1.45331 | 0.274727 | 5.290007 | 1.22E-07 | 5.94E-06 | k__Bacteria | p__Firmicutes | c__Clostridia | o__Clostridiales | f__Lachnospiraceae | g__ | s__ |
| denovo24 | 575.884 | -1.60223 | 0.306159 | -5.23335 | 1.66E-07 | 7.71E-06 | k__Bacteria | p__Bacteroidetes | c__Bacteroidia | o__Bacteroidales | f__Rikenellaceae | NA | NA |
| denovo167 | 23.16944 | -5.11348 | 0.98029 | -5.21629 | 1.83E-07 | 7.71E-06 | k__Bacteria | p__Bacteroidetes | c__Bacteroidia | o__Bacteroidales | f__S24-7 | g__ | s__ |
| denovo206 | 11.95427 | -6.08975 | 1.167302 | -5.21694 | 1.82E-07 | 7.71E-06 | k__Bacteria | p__Tenericutes | c__Mollicutes | o__RF39 | f__ | g__ | s__ |
| denovo158 | 14.6682 | -5.08657 | 0.978671 | -5.19742 | 2.02E-07 | 8.18E-06 | k__Bacteria | p__Firmicutes | c__Clostridia | o__Clostridiales | f__Ruminococcaceae | g__ | s__ |
| denovo112 | 25.83392 | -3.68541 | 0.710344 | -5.1882 | 2.12E-07 | 8.26E-06 | k__Bacteria | p__Proteobacteria | c__Alphaproteobacteria | o__RF32 | f__ | g__ | s__ |
| denovo40 | 225.0964 | -2.57016 | 0.500941 | -5.13066 | 2.89E-07 | 1.08E-05 | k__Bacteria | p__Bacteroidetes | c__Bacteroidia | o__Bacteroidales | f__Bacteroidaceae | g__Bacteroides | s__ |
| denovo60 | 259.7595 | 1.376539 | 0.269754 | 5.102943 | 3.34E-07 | 1.20E-05 | k__Bacteria | p__Firmicutes | c__Bacilli | o__Lactobacillales | f__Streptococcaceae | g__Streptococcus | s__ |
| denovo753 | 14.88162 | 2.41903 | 0.474942 | 5.09331 | 3.52E-07 | 1.22E-05 | k__Bacteria | p__Firmicutes | c__Clostridia | o__Clostridiales | f__Lachnospiraceae | g__ | s__ |
| denovo95 | 107.8416 | -2.17435 | 0.428463 | -5.07476 | 3.88E-07 | 1.30E-05 | k__Bacteria | p__Firmicutes | c__Clostridia | o__Clostridiales | f__Ruminococcaceae | g__Ruminococcus | s__ |
| denovo78 | 63.52 | -2.98224 | 0.589924 | -5.0553 | 4.30E-07 | 1.39E-05 | k__Bacteria | p__Firmicutes | c__Clostridia | o__Clostridiales | f__Lachnospiraceae | g__ | s__ |
| denovo170 | 9.207268 | -7.45858 | 1.490432 | -5.0043 | 5.61E-07 | 1.76E-05 | k__Bacteria | p__Bacteroidetes | c__Bacteroidia | o__Bacteroidales | f__S24-7 | g__ | s__ |
| denovo235 | 10.2532 | 2.066605 | 0.413992 | 4.991893 | 5.98E-07 | 1.82E-05 | k__Bacteria | p__Firmicutes | c__Bacilli | o__Lactobacillales | f__Enterococcaceae | g__Enterococcus | s__ |
| denovo18 | 1807.735 | 0.995825 | 0.199886 | 4.98197 | 6.29E-07 | 1.85E-05 | k__Bacteria | p__Bacteroidetes | c__Bacteroidia | o__Bacteroidales | f__Bacteroidaceae | g__Bacteroides | s__ |
| denovo983 | 7.37916 | -2.05336 | 0.416577 | -4.92912 | 8.26E-07 | 2.36E-05 | k__Bacteria | p__Bacteroidetes | c__Bacteroidia | o__Bacteroidales | f__Bacteroidaceae | g__Bacteroides | s__ |
| denovo142 | 76.70004 | 1.103909 | 0.224285 | 4.921908 | 8.57E-07 | 2.38E-05 | k__Bacteria | p__Firmicutes | c__Clostridia | o__Clostridiales | f__Ruminococcaceae | g__Oscillospira | s__ |
| denovo492 | 3.093508 | -3.97422 | 0.810922 | -4.90087 | 9.54E-07 | 2.58E-05 | k__Bacteria | p__Firmicutes | c__Clostridia | o__Clostridiales | f__ | g__ | s__ |
| denovo582 | 1.361827 | 2.704297 | 0.55414 | 4.88017 | 1.06E-06 | 2.78E-05 | k__Bacteria | p__Firmicutes | c__Bacilli | o__Lactobacillales | f__Streptococcaceae | g__Streptococcus | s__ |
| denovo116 | 58.37776 | -2.15817 | 0.445153 | -4.84816 | 1.25E-06 | 3.19E-05 | k__Bacteria | p__Bacteroidetes | c__Bacteroidia | o__Bacteroidales | f__Rikenellaceae | g__ | s__ |
| denovo108 | 17.87956 | -5.07458 | 1.051525 | -4.82592 | 1.39E-06 | 3.39E-05 | k__Bacteria | p__Tenericutes | c__Mollicutes | o__RF39 | f__ | g__ | s__ |
| denovo389 | 4.804816 | -3.89697 | 0.80685 | -4.82986 | 1.37E-06 | 3.39E-05 | k__Bacteria | p__Firmicutes | c__Clostridia | o__Clostridiales | f__ | g__ | s__ |
| denovo581 | 9.532443 | 1.597846 | 0.331525 | 4.819688 | 1.44E-06 | 3.41E-05 | k__Bacteria | p__Bacteroidetes | c__Bacteroidia | o__Bacteroidales | f__Bacteroidaceae | g__Bacteroides | s__fragilis |
| denovo201 | 8.133846 | -5.61898 | 1.178187 | -4.76917 | 1.85E-06 | 4.28E-05 | k__Bacteria | p__Firmicutes | c__Clostridia | o__Clostridiales | f__Ruminococcaceae | g__ | s__ |
| denovo546 | 14.86373 | -3.22351 | 0.678187 | -4.75313 | 2.00E-06 | 4.53E-05 | k__Bacteria | p__Firmicutes | c__Clostridia | o__Clostridiales | f__ | g__ | s__ |
| denovo154 | 17.29548 | -3.39761 | 0.731674 | -4.64361 | 3.42E-06 | 7.56E-05 | k__Bacteria | p__Firmicutes | c__Clostridia | o__Clostridiales | f__ | g__ | s__ |
| denovo217 | 19.37094 | 1.749127 | 0.377049 | 4.638997 | 3.50E-06 | 7.56E-05 | k__Bacteria | p__Firmicutes | c__Clostridia | o__Clostridiales | f__Ruminococcaceae | g__Oscillospira | s__ |
| denovo1237 | 2.187061 | 1.806858 | 0.393786 | 4.588421 | 4.47E-06 | 9.44E-05 | k__Bacteria | p__Actinobacteria | c__Actinobacteria | o__Bifidobacteriales | f__Bifidobacteriaceae | g__Bifidobacterium | s__ |
| denovo46 | 151.077 | -2.40951 | 0.528481 | -4.55931 | 5.13E-06 | 0.000106 | k__Bacteria | p__Bacteroidetes | c__Bacteroidia | o__Bacteroidales | f__Bacteroidaceae | g__Bacteroides | s__ |
| denovo604 | 5.035271 | -1.61872 | 0.35697 | -4.5346 | 5.77E-06 | 0.000117 | k__Bacteria | p__Firmicutes | c__Clostridia | o__Clostridiales | f__ | g__ | s__ |
| denovo182 | 23.17258 | 1.22147 | 0.269864 | 4.526238 | 6.00E-06 | 0.000119 | k__Bacteria | p__Firmicutes | c__Clostridia | o__Clostridiales | f__Ruminococcaceae | g__Ruminococcus | s__ |
| denovo268 | 11.1336 | -4.14942 | 0.926647 | -4.47788 | 7.54E-06 | 0.000147 | k__Bacteria | p__Bacteroidetes | c__Bacteroidia | o__Bacteroidales | f__Rikenellaceae | g__ | s__ |
| denovo1292 | 2.886905 | -3.79713 | 0.850756 | -4.46325 | 8.07E-06 | 0.000151 | k__Bacteria | p__Bacteroidetes | c__Bacteroidia | o__Bacteroidales | f__Rikenellaceae | g__ | s__ |
| denovo173 | 14.96511 | -3.92062 | 0.87826 | -4.46408 | 8.04E-06 | 0.000151 | k__Bacteria | p__Tenericutes | c__Mollicutes | o__RF39 | f__ | g__ | s__ |
| denovo936 | 1.977097 | -1.6121 | 0.363659 | -4.433 | 9.29E-06 | 0.00017 | k__Bacteria | p__Firmicutes | c__Clostridia | o__Clostridiales | f__Ruminococcaceae | g__Oscillospira | s__ |
| denovo140 | 18.37205 | -3.41721 | 0.774387 | -4.41279 | 1.02E-05 | 0.000184 | k__Bacteria | p__Proteobacteria | c__Alphaproteobacteria | o__RF32 | f__ | g__ | s__ |
| denovo507 | 4.346816 | -3.63609 | 0.828179 | -4.39046 | 1.13E-05 | 0.0002 | k__Bacteria | p__Firmicutes | c__Clostridia | o__Clostridiales | f__Ruminococcaceae | g__ | s__ |
| denovo381 | 9.763838 | -1.76266 | 0.406312 | -4.3382 | 1.44E-05 | 0.000249 | k__Bacteria | p__Firmicutes | c__Clostridia | o__Clostridiales | f__Ruminococcaceae | g__ | s__ |
| denovo130 | 72.28902 | -3.12244 | 0.730205 | -4.27611 | 1.90E-05 | 0.000324 | k__Bacteria | p__Bacteroidetes | c__Bacteroidia | o__Bacteroidales | f__[Paraprevotellaceae] | g__Paraprevotella | s__ |
| denovo438 | 6.652068 | -1.24489 | 0.292763 | -4.2522 | 2.12E-05 | 0.000355 | k__Bacteria | p__Firmicutes | c__Clostridia | o__Clostridiales | f__Ruminococcaceae | g__ | s__ |
| denovo138 | 253.5578 | 1.062309 | 0.250946 | 4.233217 | 2.30E-05 | 0.000373 | k__Bacteria | p__Bacteroidetes | c__Bacteroidia | o__Bacteroidales | f__Bacteroidaceae | g__Bacteroides | s__ovatus |
| denovo36 | 442.9837 | -1.58809 | 0.375013 | -4.23478 | 2.29E-05 | 0.000373 | k__Bacteria | p__Firmicutes | c__Clostridia | o__Clostridiales | f__ | g__ | s__ |
| denovo352 | 7.257045 | -3.17536 | 0.751719 | -4.22413 | 2.40E-05 | 0.000382 | k__Bacteria | p__Firmicutes | c__Clostridia | o__Clostridiales | f__Ruminococcaceae | g__ | s__ |
| denovo260 | 7.587937 | -4.9141 | 1.168714 | -4.2047 | 2.61E-05 | 0.00041 | k__Bacteria | p__Firmicutes | c__Clostridia | o__Clostridiales | f__ | g__ | s__ |
| denovo15 | 3004.782 | 0.804778 | 0.192977 | 4.170333 | 3.04E-05 | 0.000469 | k__Bacteria | p__Bacteroidetes | c__Bacteroidia | o__Bacteroidales | f__Bacteroidaceae | g__Bacteroides | s__ |
| denovo26 | 898.3827 | -0.92611 | 0.222404 | -4.1641 | 3.13E-05 | 0.000475 | k__Bacteria | p__Firmicutes | c__Clostridia | o__Clostridiales | f__Lachnospiraceae | g__ | s__ |
| denovo278 | 8.01777 | 1.572194 | 0.378934 | 4.148991 | 3.34E-05 | 0.000499 | k__Bacteria | p__Firmicutes | c__Erysipelotrichi | o__Erysipelotrichales | f__Erysipelotrichaceae | g__[Eubacterium] | s__dolichum |
| denovo22 | 845.2048 | 1.066479 | 0.258219 | 4.13014 | 3.63E-05 | 0.000534 | k__Bacteria | p__Actinobacteria | c__Actinobacteria | o__Bifidobacteriales | f__Bifidobacteriaceae | g__Bifidobacterium | s__adolescentis |
| denovo123 | 59.02245 | -1.6219 | 0.394204 | -4.11436 | 3.88E-05 | 0.000563 | k__Bacteria | p__Firmicutes | c__Clostridia | o__Clostridiales | f__Ruminococcaceae | g__ | s__ |
| denovo162 | 20.50342 | -3.14955 | 0.769467 | -4.09315 | 4.26E-05 | 0.0006 | k__Bacteria | p__Firmicutes | c__Clostridia | o__Clostridiales | f__Ruminococcaceae | g__Ruminococcus | s__ |
| denovo168 | 15.76841 | -2.78186 | 0.679675 | -4.09292 | 4.26E-05 | 0.0006 | k__Bacteria | p__Proteobacteria | c__Alphaproteobacteria | o__RF32 | f__ | g__ | s__ |
| denovo233 | 10.18366 | -2.82754 | 0.699456 | -4.04248 | 5.29E-05 | 0.000734 | k__Bacteria | p__Firmicutes | c__Clostridia | o__Clostridiales | f__Christensenellaceae | g__ | s__ |
| denovo263 | 7.114765 | -3.80251 | 0.948113 | -4.01061 | 6.06E-05 | 0.000829 | k__Bacteria | p__Tenericutes | c__RF3 | o__ML615J-28 | f__ | g__ | s__ |
| denovo427 | 3.260473 | -3.67296 | 0.919989 | -3.99239 | 6.54E-05 | 0.000883 | k__Bacteria | p__Verrucomicrobia | c__Opitutae | o__[Cerasicoccales] | f__[Cerasicoccaceae] | g__ | s__ |
| denovo337 | 11.35312 | -4.14273 | 1.038669 | -3.9885 | 6.65E-05 | 0.000885 | k__Bacteria | p__Firmicutes | c__Clostridia | o__Clostridiales | f__Ruminococcaceae | g__ | s__ |
| denovo212 | 16.22457 | -2.56893 | 0.647439 | -3.96783 | 7.25E-05 | 0.000953 | k__Bacteria | p__Firmicutes | c__Clostridia | o__Clostridiales | f__Ruminococcaceae | g__ | s__ |
| denovo321 | 5.67716 | -4.06116 | 1.033829 | -3.92828 | 8.56E-05 | 0.001109 | k__Bacteria | p__Firmicutes | c__Clostridia | o__Clostridiales | f__Ruminococcaceae | g__ | s__ |
| denovo350 | 9.249253 | -1.56237 | 0.400416 | -3.90188 | 9.54E-05 | 0.001221 | k__Bacteria | p__Firmicutes | c__Erysipelotrichi | o__Erysipelotrichales | f__Erysipelotrichaceae | g__cc_115 | s__ |
| denovo401 | 4.461537 | -4.43036 | 1.137712 | -3.8941 | 9.86E-05 | 0.001244 | k__Bacteria | p__Tenericutes | c__Mollicutes | o__RF39 | f__ | g__ | s__ |
| denovo133 | 142.123 | 1.327634 | 0.342787 | 3.873062 | 0.000107 | 0.001339 | k__Bacteria | p__Firmicutes | c__Clostridia | o__Clostridiales | f__Veillonellaceae | g__Veillonella | s__dispar |
| denovo411 | 6.896978 | -3.15192 | 0.822989 | -3.82985 | 0.000128 | 0.001578 | k__Bacteria | p__Firmicutes | c__Clostridia | o__Clostridiales | f__ | g__ | s__ |
| denovo322 | 4.02294 | -3.93039 | 1.052039 | -3.73598 | 0.000187 | 0.002272 | k__Bacteria | p__Tenericutes | c__Mollicutes | o__RF39 | f__ | g__ | s__ |
| denovo151 | 31.27266 | -2.36541 | 0.639105 | -3.70112 | 0.000215 | 0.002504 | k__Bacteria | p__Firmicutes | c__Clostridia | o__Clostridiales | f__ | g__ | s__ |
| denovo210 | 19.70616 | -3.46507 | 0.935789 | -3.70283 | 0.000213 | 0.002504 | k__Bacteria | p__Firmicutes | c__Clostridia | o__Clostridiales | f__Ruminococcaceae | g__ | s__ |
| denovo228 | 5.194358 | -4.12591 | 1.115396 | -3.69905 | 0.000216 | 0.002504 | k__Bacteria | p__Firmicutes | c__Clostridia | o__Clostridiales | f__Veillonellaceae | g__Dialister | s__ |
| denovo559 | 1.975523 | -2.68325 | 0.723706 | -3.70766 | 0.000209 | 0.002504 | k__Bacteria | p__Firmicutes | c__Clostridia | o__Clostridiales | f__Ruminococcaceae | g__ | s__ |
| denovo591 | 1.680499 | -2.68319 | 0.726244 | -3.69462 | 0.00022 | 0.002518 | k__Bacteria | p__Firmicutes | c__Clostridia | o__Clostridiales | f__ | g__ | s__ |
| denovo528 | 1.695126 | -3.06498 | 0.836203 | -3.66535 | 0.000247 | 0.002792 | k__Bacteria | p__Lentisphaerae | c__[Lentisphaeria] | o__Victivallales | f__Victivallaceae | g__ | s__ |
| denovo254 | 7.910114 | -1.83054 | 0.50167 | -3.64889 | 0.000263 | 0.002943 | k__Bacteria | p__Firmicutes | c__Clostridia | o__Clostridiales | f__ | g__ | s__ |
| denovo1686 | 2.125497 | -2.02733 | 0.556947 | -3.64008 | 0.000273 | 0.00301 | k__Bacteria | p__Firmicutes | c__Clostridia | o__Clostridiales | f__Ruminococcaceae | g__ | s__ |
| denovo164 | 14.48297 | -3.46664 | 0.956616 | -3.62385 | 0.00029 | 0.003091 | k__Bacteria | p__Proteobacteria | c__Betaproteobacteria | o__Burkholderiales | f__Comamonadaceae | g__ | s__ |
| denovo186 | 6.747992 | -3.59062 | 0.992045 | -3.61941 | 0.000295 | 0.003091 | k__Bacteria | p__Firmicutes | c__Clostridia | o__Clostridiales | f__Christensenellaceae | g__ | s__ |
| denovo27 | 597.5102 | 0.652863 | 0.179862 | 3.629809 | 0.000284 | 0.003091 | k__Bacteria | p__Firmicutes | c__Clostridia | o__Clostridiales | f__Lachnospiraceae | g__[Ruminococcus] | s__gnavus |
| denovo361 | 24.49448 | 0.972626 | 0.268758 | 3.618965 | 0.000296 | 0.003091 | k__Bacteria | p__Firmicutes | c__Bacilli | o__Lactobacillales | f__Streptococcaceae | g__Streptococcus | s__ |
| denovo406 | 2.889203 | -4.56602 | 1.259488 | -3.6253 | 0.000289 | 0.003091 | k__Bacteria | p__Tenericutes | c__Mollicutes | o__RF39 | f__ | g__ | s__ |
| denovo272 | 24.17765 | 1.080685 | 0.299109 | 3.613011 | 0.000303 | 0.003097 | k__Bacteria | p__Firmicutes | c__Clostridia | o__Clostridiales | f__Lachnospiraceae | g__ | s__ |
| denovo336 | 21.30957 | -1.10064 | 0.304619 | -3.61316 | 0.000302 | 0.003097 | k__Bacteria | p__Firmicutes | c__Clostridia | o__Clostridiales | f__Ruminococcaceae | g__Oscillospira | s__ |
| denovo6053 | 1.626384 | 0.962745 | 0.266675 | 3.610181 | 0.000306 | 0.003098 | k__Bacteria | p__Bacteroidetes | c__Bacteroidia | o__Bacteroidales | f__Bacteroidaceae | g__Bacteroides | s__ |
| denovo236 | 3.835059 | -4.83854 | 1.354506 | -3.57218 | 0.000354 | 0.003548 | k__Bacteria | p__Firmicutes | c__Clostridia | o__Clostridiales | f__ | g__ | s__ |
| denovo277 | 28.0913 | -1.50323 | 0.421777 | -3.56404 | 0.000365 | 0.003599 | k__Bacteria | p__Firmicutes | c__Clostridia | o__Clostridiales | f__ | g__ | s__ |
| denovo362 | 5.52997 | 1.533098 | 0.430274 | 3.563074 | 0.000367 | 0.003599 | k__Bacteria | p__Firmicutes | c__Clostridia | o__Clostridiales | f__Lachnospiraceae | g__Dorea | s__ |
| denovo296 | 17.27303 | -2.86537 | 0.812446 | -3.52685 | 0.000421 | 0.004088 | k__Bacteria | p__Firmicutes | c__Clostridia | o__Clostridiales | f__Ruminococcaceae | g__ | s__ |
| denovo469 | 49.16887 | -1.13965 | 0.32339 | -3.52406 | 0.000425 | 0.00409 | k__Bacteria | p__Firmicutes | c__Clostridia | o__Clostridiales | f__Ruminococcaceae | g__Faecalibacterium | s__prausnitzii |
| denovo147 | 31.52112 | -2.77444 | 0.79916 | -3.4717 | 0.000517 | 0.004928 | k__Bacteria | p__Tenericutes | c__Mollicutes | o__RF39 | f__ | g__ | s__ |
| denovo432 | 2.837917 | -2.70231 | 0.787485 | -3.43157 | 0.0006 | 0.005663 | k__Bacteria | p__Firmicutes | c__Clostridia | o__Clostridiales | f__ | g__ | s__ |
| denovo72 | 274.8887 | -1.20203 | 0.351533 | -3.41939 | 0.000628 | 0.005866 | k__Bacteria | p__Firmicutes | c__Clostridia | o__Clostridiales | f__ | g__ | s__ |
| denovo129 | 68.6113 | -1.07932 | 0.319481 | -3.37834 | 0.000729 | 0.006646 | k__Bacteria | p__Firmicutes | c__Clostridia | o__Clostridiales | f__Lachnospiraceae | g__Lachnospira | s__ |
| denovo160 | 6.425847 | -4.21421 | 1.248691 | -3.3749 | 0.000738 | 0.006646 | k__Bacteria | p__Firmicutes | c__Clostridia | o__Clostridiales | f__Veillonellaceae | g__Megamonas | s__ |
| denovo482 | 5.824657 | -1.24899 | 0.369991 | -3.37574 | 0.000736 | 0.006646 | k__Bacteria | p__Firmicutes | c__Clostridia | o__Clostridiales | f__ | g__ | s__ |
| denovo73 | 196.3259 | -0.86185 | 0.255013 | -3.37963 | 0.000726 | 0.006646 | k__Bacteria | p__Firmicutes | c__Clostridia | o__Clostridiales | f__Lachnospiraceae | g__Lachnospira | s__ |
| denovo102 | 105.3545 | -0.81359 | 0.242374 | -3.35676 | 0.000789 | 0.006977 | k__Bacteria | p__Firmicutes | c__Clostridia | o__Clostridiales | f__Ruminococcaceae | g__Oscillospira | s__ |
| denovo453 | 2.59268 | -3.16773 | 0.943778 | -3.35644 | 0.00079 | 0.006977 | k__Bacteria | p__Lentisphaerae | c__[Lentisphaeria] | o__Victivallales | f__Victivallaceae | g__ | s__ |
| denovo828 | 14.05614 | -0.79408 | 0.237151 | -3.34842 | 0.000813 | 0.007117 | k__Bacteria | p__Firmicutes | c__Clostridia | o__Clostridiales | f__Lachnospiraceae | g__ | s__ |
| denovo52 | 199.815 | -1.12659 | 0.337806 | -3.33501 | 0.000853 | 0.007402 | k__Bacteria | p__Firmicutes | c__Clostridia | o__Clostridiales | f__Clostridiaceae | g__Clostridium | s__ |
| denovo49 | 168.0284 | -1.41846 | 0.427755 | -3.31605 | 0.000913 | 0.007853 | k__Bacteria | p__Firmicutes | c__Clostridia | o__Clostridiales | f__Ruminococcaceae | g__ | s__ |
| denovo349 | 5.620941 | -4.6965 | 1.418972 | -3.30979 | 0.000934 | 0.007942 | k__Bacteria | p__Tenericutes | c__Mollicutes | o__RF39 | f__ | g__ | s__ |
| denovo568 | 2.254864 | -2.67607 | 0.808968 | -3.30801 | 0.00094 | 0.007942 | k__Bacteria | p__Firmicutes | c__Clostridia | o__Clostridiales | f__Lachnospiraceae | g__ | s__ |
| denovo4 | 1647.511 | 1.195695 | 0.362146 | 3.301695 | 0.000961 | 0.008053 | k__Bacteria | p__Proteobacteria | c__Gammaproteobacteria | o__Enterobacteriales | f__Enterobacteriaceae | g__ | s__ |
| denovo1764 | 2.148453 | -1.62284 | 0.492071 | -3.29799 | 0.000974 | 0.00809 | k__Bacteria | p__Firmicutes | c__Clostridia | o__Clostridiales | f__ | g__ | s__ |
| denovo577 | 2.761879 | -2.05313 | 0.623792 | -3.29136 | 0.000997 | 0.008213 | k__Bacteria | p__Firmicutes | c__Clostridia | o__Clostridiales | f__Peptococcaceae | g__ | s__ |
| denovo495 | 4.253807 | 1.393901 | 0.424826 | 3.281112 | 0.001034 | 0.008446 | k__Bacteria | p__Firmicutes | c__Clostridia | o__Clostridiales | f__Lachnospiraceae | g__ | s__ |
| denovo823 | 3.522354 | -3.97899 | 1.213756 | -3.27825 | 0.001045 | 0.008461 | k__Bacteria | p__Firmicutes | c__Clostridia | o__Clostridiales | f__Ruminococcaceae | g__ | s__ |
| denovo556 | 1.993519 | -2.48302 | 0.760171 | -3.2664 | 0.001089 | 0.008608 | k__Bacteria | p__Firmicutes | c__Clostridia | o__Clostridiales | f__Ruminococcaceae | g__ | s__ |
| denovo6 | 1855.755 | 0.829911 | 0.254006 | 3.267295 | 0.001086 | 0.008608 | k__Bacteria | p__Firmicutes | c__Clostridia | o__Clostridiales | f__Ruminococcaceae | g__Ruminococcus | s__ |
| denovo6591 | 1.807406 | 0.907287 | 0.277491 | 3.269605 | 0.001077 | 0.008608 | k__Bacteria | p__Bacteroidetes | c__Bacteroidia | o__Bacteroidales | f__Bacteroidaceae | g__Bacteroides | s__ |
| denovo98 | 30.1405 | -2.2601 | 0.694735 | -3.25318 | 0.001141 | 0.008946 | k__Bacteria | p__Bacteroidetes | c__Bacteroidia | o__Bacteroidales | f__Bacteroidaceae | g__Bacteroides | s__ |
| denovo257 | 8.880385 | -3.33 | 1.025636 | -3.24677 | 0.001167 | 0.009033 | k__Bacteria | p__Tenericutes | c__Mollicutes | o__RF39 | f__ | g__ | s__ |
| denovo667 | 18.95036 | -1.22058 | 0.376041 | -3.24587 | 0.001171 | 0.009033 | k__Bacteria | p__Firmicutes | c__Clostridia | o__Clostridiales | f__Lachnospiraceae | g__ | s__ |
| denovo226 | 6.059253 | -3.09508 | 0.95938 | -3.22613 | 0.001255 | 0.009604 | k__Bacteria | p__Firmicutes | c__Clostridia | o__Clostridiales | f__Lachnospiraceae | g__ | s__ |
| denovo717 | 1.646237 | -1.51412 | 0.469688 | -3.22367 | 0.001266 | 0.009611 | k__Bacteria | p__Firmicutes | c__Clostridia | o__Clostridiales | f__Ruminococcaceae | g__ | s__ |

**Supplementary table 10.** Differential abundance of OTUs by Health deficit (FI) as a factor, adjusted for education level. Results are where q < 0.01

| **id** | **baseMean** | **log2FoldChange** | **lfcSE** | **stat** | **pvalue** | **padj** | **Kingdom** | **Phylum** | **Class** | **Order** | **Family** | **Genus** | **Species** |
| --- | --- | --- | --- | --- | --- | --- | --- | --- | --- | --- | --- | --- | --- |
| denovo235 | 13.92807 | 3.810325 | 0.42845 | 8.893286 | 5.93E-19 | 5.77E-16 | k__Bacteria | p__Firmicutes | c__Bacilli | o__Lactobacillales | f__Enterococcaceae | g__Enterococcus | s__ |
| denovo278 | 9.883887 | 2.974019 | 0.406037 | 7.324499 | 2.40E-13 | 1.17E-10 | k__Bacteria | p__Firmicutes | c__Erysipelotrichi | o__Erysipelotrichales | f__Erysipelotrichaceae | g__[Eubacterium] | s__dolichum |
| denovo76 | 124.1804 | -4.2297 | 0.606782 | -6.97071 | 3.15E-12 | 1.02E-09 | k__Bacteria | p__Firmicutes | c__Clostridia | o__Clostridiales | f__Ruminococcaceae | g__ | s__ |
| denovo114 | 34.04683 | -5.89727 | 0.861457 | -6.8457 | 7.61E-12 | 1.85E-09 | k__Bacteria | p__Bacteroidetes | c__Bacteroidia | o__Bacteroidales | f__Bacteroidaceae | g__Bacteroides | s__coprophilus |
| denovo139 | 43.24271 | -6.49703 | 0.976451 | -6.65371 | 2.86E-11 | 5.56E-09 | k__Bacteria | p__Tenericutes | c__Mollicutes | o__RF39 | f__ | g__ | s__ |
| denovo88 | 106.059 | 1.488275 | 0.226514 | 6.570337 | 5.02E-11 | 8.13E-09 | k__Bacteria | p__Firmicutes | c__Clostridia | o__Clostridiales | f__Ruminococcaceae | g__ | s__ |
| denovo165 | 46.32019 | 1.848403 | 0.290757 | 6.357204 | 2.05E-10 | 2.85E-08 | k__Bacteria | p__Firmicutes | c__Clostridia | o__Clostridiales | f__Lachnospiraceae | g__ | s__ |
| denovo158 | 22.05752 | -6.32965 | 0.999207 | -6.33468 | 2.38E-10 | 2.89E-08 | k__Bacteria | p__Firmicutes | c__Clostridia | o__Clostridiales | f__Ruminococcaceae | g__ | s__ |
| denovo119 | 66.94926 | -5.58558 | 0.889871 | -6.27684 | 3.46E-10 | 3.36E-08 | k__Bacteria | p__Tenericutes | c__Mollicutes | o__RF39 | f__ | g__ | s__ |
| denovo80 | 37.02218 | -4.50759 | 0.716572 | -6.29049 | 3.16E-10 | 3.36E-08 | k__Bacteria | p__Firmicutes | c__Clostridia | o__Clostridiales | f__ | g__ | s__ |
| denovo93 | 89.43914 | -5.12072 | 0.820607 | -6.24016 | 4.37E-10 | 3.86E-08 | k__Bacteria | p__Tenericutes | c__Mollicutes | o__RF39 | f__ | g__ | s__ |
| denovo153 | 41.09861 | 2.47767 | 0.401003 | 6.17868 | 6.46E-10 | 5.24E-08 | k__Bacteria | p__Firmicutes | c__Clostridia | o__Clostridiales | f__Lachnospiraceae | g__Dorea | s__ |
| denovo180 | 35.60444 | -2.45675 | 0.401623 | -6.11706 | 9.53E-10 | 6.62E-08 | k__Bacteria | p__Firmicutes | c__Clostridia | o__Clostridiales | f__ | g__ | s__ |
| denovo85 | 45.22994 | -5.43953 | 0.889042 | -6.11842 | 9.45E-10 | 6.62E-08 | k__Bacteria | p__Tenericutes | c__Mollicutes | o__RF39 | f__ | g__ | s__ |
| denovo176 | 12.31202 | -6.87473 | 1.132117 | -6.07245 | 1.26E-09 | 8.16E-08 | k__Bacteria | p__Bacteroidetes | c__Bacteroidia | o__Bacteroidales | f__ | g__ | s__ |
| denovo218 | 47.52788 | -1.82967 | 0.302988 | -6.03875 | 1.55E-09 | 8.88E-08 | k__Bacteria | p__Firmicutes | c__Clostridia | o__Clostridiales | f__Lachnospiraceae | g__ | s__ |
| denovo25 | 805.0458 | 1.065134 | 0.176369 | 6.039241 | 1.55E-09 | 8.88E-08 | k__Bacteria | p__Firmicutes | c__Clostridia | o__Clostridiales | f__Lachnospiraceae | g__Blautia | s__ |
| denovo136 | 55.31846 | -2.67663 | 0.451721 | -5.9254 | 3.12E-09 | 1.68E-07 | k__Bacteria | p__Firmicutes | c__Clostridia | o__Clostridiales | f__ | g__ | s__ |
| denovo77 | 26.73168 | -4.5444 | 0.801997 | -5.66636 | 1.46E-08 | 7.46E-07 | k__Bacteria | p__Bacteroidetes | c__Bacteroidia | o__Bacteroidales | f__Prevotellaceae | g__Prevotella | s__ |
| denovo133 | 166.81 | 2.035614 | 0.361399 | 5.632596 | 1.78E-08 | 8.63E-07 | k__Bacteria | p__Firmicutes | c__Clostridia | o__Clostridiales | f__Veillonellaceae | g__Veillonella | s__dispar |
| denovo206 | 11.51555 | -6.25211 | 1.178501 | -5.30513 | 1.13E-07 | 5.21E-06 | k__Bacteria | p__Tenericutes | c__Mollicutes | o__RF39 | f__ | g__ | s__ |
| denovo492 | 3.224334 | -4.2489 | 0.815401 | -5.21081 | 1.88E-07 | 8.31E-06 | k__Bacteria | p__Firmicutes | c__Clostridia | o__Clostridiales | f__ | g__ | s__ |
| denovo364 | 9.36689 | 3.084848 | 0.601194 | 5.131206 | 2.88E-07 | 1.22E-05 | k__Bacteria | p__Firmicutes | c__Clostridia | o__Clostridiales | f__Lachnospiraceae | g__[Ruminococcus] | s__ |
| denovo449 | 14.09767 | 1.477305 | 0.289652 | 5.100279 | 3.39E-07 | 1.37E-05 | k__Bacteria | p__Bacteroidetes | c__Bacteroidia | o__Bacteroidales | f__Bacteroidaceae | g__Bacteroides | s__ |
| denovo24 | 596.2805 | -1.61586 | 0.31962 | -5.05556 | 4.29E-07 | 1.67E-05 | k__Bacteria | p__Bacteroidetes | c__Bacteroidia | o__Bacteroidales | f__Rikenellaceae | NA | NA |
| denovo40 | 406.4829 | -2.66382 | 0.529154 | -5.03412 | 4.80E-07 | 1.79E-05 | k__Bacteria | p__Bacteroidetes | c__Bacteroidia | o__Bacteroidales | f__Bacteroidaceae | g__Bacteroides | s__ |
| denovo60 | 252.1968 | 1.381792 | 0.277156 | 4.985619 | 6.18E-07 | 2.22E-05 | k__Bacteria | p__Firmicutes | c__Bacilli | o__Lactobacillales | f__Streptococcaceae | g__Streptococcus | s__ |
| denovo18 | 1855.525 | 1.015413 | 0.205853 | 4.932706 | 8.11E-07 | 2.82E-05 | k__Bacteria | p__Bacteroidetes | c__Bacteroidia | o__Bacteroidales | f__Bacteroidaceae | g__Bacteroides | s__ |
| denovo182 | 23.13041 | 1.386538 | 0.283137 | 4.897052 | 9.73E-07 | 3.26E-05 | k__Bacteria | p__Firmicutes | c__Clostridia | o__Clostridiales | f__Ruminococcaceae | g__Ruminococcus | s__ |
| denovo753 | 14.78208 | 2.338309 | 0.487733 | 4.794239 | 1.63E-06 | 5.29E-05 | k__Bacteria | p__Firmicutes | c__Clostridia | o__Clostridiales | f__Lachnospiraceae | g__ | s__ |
| denovo116 | 60.37839 | -2.19523 | 0.460545 | -4.76659 | 1.87E-06 | 5.87E-05 | k__Bacteria | p__Bacteroidetes | c__Bacteroidia | o__Bacteroidales | f__Rikenellaceae | g__ | s__ |
| denovo581 | 9.715859 | 1.647768 | 0.347762 | 4.738208 | 2.16E-06 | 6.55E-05 | k__Bacteria | p__Bacteroidetes | c__Bacteroidia | o__Bacteroidales | f__Bacteroidaceae | g__Bacteroides | s__fragilis |
| denovo95 | 108.8278 | -2.04012 | 0.432795 | -4.71383 | 2.43E-06 | 7.16E-05 | k__Bacteria | p__Firmicutes | c__Clostridia | o__Clostridiales | f__Ruminococcaceae | g__Ruminococcus | s__ |
| denovo61 | 153.0968 | 2.693097 | 0.572546 | 4.703723 | 2.55E-06 | 7.30E-05 | k__Bacteria | p__Proteobacteria | c__Gammaproteobacteria | o__Enterobacteriales | f__Enterobacteriaceae | g__ | s__ |
| denovo161 | 37.49926 | 2.16974 | 0.466083 | 4.655266 | 3.24E-06 | 8.99E-05 | k__Bacteria | p__Firmicutes | c__Erysipelotrichi | o__Erysipelotrichales | f__Erysipelotrichaceae | g__ | s__ |
| denovo616 | 2.506102 | 4.261499 | 0.921834 | 4.622851 | 3.79E-06 | 0.000101 | k__Bacteria | p__Firmicutes | c__Clostridia | o__Clostridiales | f__Lachnospiraceae | g__Coprococcus | s__ |
| denovo92 | 20.56229 | 3.8337 | 0.829703 | 4.620572 | 3.83E-06 | 0.000101 | k__Bacteria | p__Bacteroidetes | c__Bacteroidia | o__Bacteroidales | f__ | g__ | s__ |
| denovo350 | 10.36816 | -1.91586 | 0.416377 | -4.60127 | 4.20E-06 | 0.000107 | k__Bacteria | p__Firmicutes | c__Erysipelotrichi | o__Erysipelotrichales | f__Erysipelotrichaceae | g__cc_115 | s__ |
| denovo168 | 19.69802 | -3.23832 | 0.70715 | -4.5794 | 4.66E-06 | 0.000116 | k__Bacteria | p__Proteobacteria | c__Alphaproteobacteria | o__RF32 | f__ | g__ | s__ |
| denovo381 | 10.03462 | -1.9116 | 0.418414 | -4.56869 | 4.91E-06 | 0.000119 | k__Bacteria | p__Firmicutes | c__Clostridia | o__Clostridiales | f__Ruminococcaceae | g__ | s__ |
| denovo22 | 823.1074 | 1.202958 | 0.264338 | 4.550825 | 5.34E-06 | 0.000127 | k__Bacteria | p__Actinobacteria | c__Actinobacteria | o__Bifidobacteriales | f__Bifidobacteriaceae | g__Bifidobacterium | s__adolescentis |
| denovo389 | 4.145429 | -3.66914 | 0.823741 | -4.45424 | 8.42E-06 | 0.000195 | k__Bacteria | p__Firmicutes | c__Clostridia | o__Clostridiales | f__ | g__ | s__ |
| denovo123 | 61.82454 | -1.76556 | 0.403947 | -4.37077 | 1.24E-05 | 0.00028 | k__Bacteria | p__Firmicutes | c__Clostridia | o__Clostridiales | f__Ruminococcaceae | g__ | s__ |
| denovo438 | 6.87767 | -1.33518 | 0.306919 | -4.35026 | 1.36E-05 | 0.0003 | k__Bacteria | p__Firmicutes | c__Clostridia | o__Clostridiales | f__Ruminococcaceae | g__ | s__ |
| denovo582 | 1.283814 | 2.498746 | 0.575341 | 4.343068 | 1.41E-05 | 0.000303 | k__Bacteria | p__Firmicutes | c__Bacilli | o__Lactobacillales | f__Streptococcaceae | g__Streptococcus | s__ |
| denovo217 | 19.20037 | 1.663712 | 0.386871 | 4.300431 | 1.70E-05 | 0.00036 | k__Bacteria | p__Firmicutes | c__Clostridia | o__Clostridiales | f__Ruminococcaceae | g__Oscillospira | s__ |
| denovo604 | 5.432027 | -1.58659 | 0.370176 | -4.28605 | 1.82E-05 | 0.000376 | k__Bacteria | p__Firmicutes | c__Clostridia | o__Clostridiales | f__ | g__ | s__ |
| denovo201 | 5.224228 | -4.92856 | 1.159479 | -4.25067 | 2.13E-05 | 0.000432 | k__Bacteria | p__Firmicutes | c__Clostridia | o__Clostridiales | f__Ruminococcaceae | g__ | s__ |
| denovo401 | 4.438266 | -4.85937 | 1.147475 | -4.23484 | 2.29E-05 | 0.000454 | k__Bacteria | p__Tenericutes | c__Mollicutes | o__RF39 | f__ | g__ | s__ |
| denovo1237 | 2.188022 | 1.695113 | 0.401609 | 4.220802 | 2.43E-05 | 0.000464 | k__Bacteria | p__Actinobacteria | c__Actinobacteria | o__Bifidobacteriales | f__Bifidobacteriaceae | g__Bifidobacterium | s__ |
| denovo1686 | 2.256564 | -2.42966 | 0.575467 | -4.22206 | 2.42E-05 | 0.000464 | k__Bacteria | p__Firmicutes | c__Clostridia | o__Clostridiales | f__Ruminococcaceae | g__ | s__ |
| denovo108 | 16.56556 | -4.44125 | 1.065196 | -4.16942 | 3.05E-05 | 0.000562 | k__Bacteria | p__Tenericutes | c__Mollicutes | o__RF39 | f__ | g__ | s__ |
| denovo212 | 19.24651 | -2.76591 | 0.663529 | -4.16848 | 3.07E-05 | 0.000562 | k__Bacteria | p__Firmicutes | c__Clostridia | o__Clostridiales | f__Ruminococcaceae | g__ | s__ |
| denovo7275 | 3.018238 | 1.766346 | 0.428216 | 4.124891 | 3.71E-05 | 0.000668 | k__Bacteria | p__Bacteroidetes | c__Bacteroidia | o__Bacteroidales | f__Bacteroidaceae | g__Bacteroides | s__fragilis |
| denovo461 | 4.703968 | 2.520289 | 0.61754 | 4.081177 | 4.48E-05 | 0.000792 | k__Bacteria | p__Firmicutes | c__Bacilli | o__Lactobacillales | f__Lactobacillaceae | g__Lactobacillus | s__zeae |
| denovo109 | 102.9246 | -2.0499 | 0.503286 | -4.07304 | 4.64E-05 | 0.000805 | k__Bacteria | p__Firmicutes | c__Clostridia | o__Clostridiales | f__ | g__ | s__ |
| denovo142 | 78.71592 | 0.945196 | 0.233439 | 4.049005 | 5.14E-05 | 0.000877 | k__Bacteria | p__Firmicutes | c__Clostridia | o__Clostridiales | f__Ruminococcaceae | g__Oscillospira | s__ |
| denovo151 | 40.69152 | -2.70998 | 0.671434 | -4.0361 | 5.43E-05 | 0.000911 | k__Bacteria | p__Firmicutes | c__Clostridia | o__Clostridiales | f__ | g__ | s__ |
| denovo138 | 263.8555 | 1.023084 | 0.256149 | 3.994096 | 6.49E-05 | 0.00107 | k__Bacteria | p__Bacteroidetes | c__Bacteroidia | o__Bacteroidales | f__Bacteroidaceae | g__Bacteroides | s__ovatus |
| denovo164 | 16.0644 | -3.89236 | 0.976042 | -3.9879 | 6.67E-05 | 0.00108 | k__Bacteria | p__Proteobacteria | c__Betaproteobacteria | o__Burkholderiales | f__Comamonadaceae | g__ | s__ |
| denovo1292 | 2.892505 | -3.55179 | 0.894533 | -3.97056 | 7.17E-05 | 0.001143 | k__Bacteria | p__Bacteroidetes | c__Bacteroidia | o__Bacteroidales | f__Rikenellaceae | g__ | s__ |
| denovo236 | 5.587889 | -5.22596 | 1.333959 | -3.91763 | 8.94E-05 | 0.001402 | k__Bacteria | p__Firmicutes | c__Clostridia | o__Clostridiales | f__ | g__ | s__ |
| denovo11 | 1236.945 | 1.067076 | 0.273876 | 3.896206 | 9.77E-05 | 0.001508 | k__Bacteria | p__Bacteroidetes | c__Bacteroidia | o__Bacteroidales | f__Porphyromonadaceae | g__Parabacteroides | s__ |
| denovo140 | 20.03197 | -3.13198 | 0.80599 | -3.88588 | 0.000102 | 0.001549 | k__Bacteria | p__Proteobacteria | c__Alphaproteobacteria | o__RF32 | f__ | g__ | s__ |
| denovo27 | 578.2477 | 0.716736 | 0.185433 | 3.865198 | 0.000111 | 0.00165 | k__Bacteria | p__Firmicutes | c__Clostridia | o__Clostridiales | f__Lachnospiraceae | g__[Ruminococcus] | s__gnavus |
| denovo591 | 1.687058 | -2.83231 | 0.733201 | -3.86294 | 0.000112 | 0.00165 | k__Bacteria | p__Firmicutes | c__Clostridia | o__Clostridiales | f__ | g__ | s__ |
| denovo322 | 3.833658 | -4.15281 | 1.078361 | -3.85104 | 0.000118 | 0.001706 | k__Bacteria | p__Tenericutes | c__Mollicutes | o__RF39 | f__ | g__ | s__ |
| denovo411 | 6.911394 | -3.2202 | 0.839224 | -3.83712 | 0.000124 | 0.001779 | k__Bacteria | p__Firmicutes | c__Clostridia | o__Clostridiales | f__ | g__ | s__ |
| denovo496 | 1.206161 | 3.51395 | 0.91732 | 3.830671 | 0.000128 | 0.0018 | k__Bacteria | p__Firmicutes | c__Clostridia | o__Clostridiales | f__Clostridiaceae | g__Clostridium | NA |
| denovo528 | 1.454886 | -3.13 | 0.822968 | -3.80331 | 0.000143 | 0.001983 | k__Bacteria | p__Lentisphaerae | c__[Lentisphaeria] | o__Victivallales | f__Victivallaceae | g__ | s__ |
| denovo170 | 5.747028 | -5.74593 | 1.52096 | -3.77783 | 0.000158 | 0.002157 | k__Bacteria | p__Bacteroidetes | c__Bacteroidia | o__Bacteroidales | f__S24-7 | g__ | s__ |
| denovo52 | 213.421 | -1.32443 | 0.350812 | -3.77533 | 0.00016 | 0.002157 | k__Bacteria | p__Firmicutes | c__Clostridia | o__Clostridiales | f__Clostridiaceae | g__Clostridium | s__ |
| denovo228 | 4.153859 | -4.14453 | 1.100769 | -3.76512 | 0.000166 | 0.002217 | k__Bacteria | p__Firmicutes | c__Clostridia | o__Clostridiales | f__Veillonellaceae | g__Dialister | s__ |
| denovo254 | 8.956546 | -1.9253 | 0.512618 | -3.75582 | 0.000173 | 0.002233 | k__Bacteria | p__Firmicutes | c__Clostridia | o__Clostridiales | f__ | g__ | s__ |
| denovo263 | 6.003711 | -3.62424 | 0.964968 | -3.75581 | 0.000173 | 0.002233 | k__Bacteria | p__Tenericutes | c__RF3 | o__ML615J-28 | f__ | g__ | s__ |
| denovo427 | 2.985554 | -3.52069 | 0.938052 | -3.7532 | 0.000175 | 0.002233 | k__Bacteria | p__Verrucomicrobia | c__Opitutae | o__[Cerasicoccales] | f__[Cerasicoccaceae] | g__ | s__ |
| denovo361 | 22.571 | 1.001215 | 0.268108 | 3.734375 | 0.000188 | 0.002375 | k__Bacteria | p__Firmicutes | c__Bacilli | o__Lactobacillales | f__Streptococcaceae | g__Streptococcus | s__ |
| denovo150 | 33.62461 | -3.12482 | 0.839065 | -3.72417 | 0.000196 | 0.002442 | k__Bacteria | p__Firmicutes | c__Clostridia | o__Clostridiales | f__Ruminococcaceae | g__Ruminococcus | s__ |
| denovo321 | 6.269846 | -3.85338 | 1.061885 | -3.62881 | 0.000285 | 0.00346 | k__Bacteria | p__Firmicutes | c__Clostridia | o__Clostridiales | f__Ruminococcaceae | g__ | s__ |
| denovo73 | 207.4213 | -0.94798 | 0.261008 | -3.632 | 0.000281 | 0.00346 | k__Bacteria | p__Firmicutes | c__Clostridia | o__Clostridiales | f__Lachnospiraceae | g__Lachnospira | s__ |
| denovo1460 | 1.148039 | 2.03921 | 0.567853 | 3.591087 | 0.000329 | 0.003766 | k__Bacteria | p__Firmicutes | c__Clostridia | o__Clostridiales | f__Veillonellaceae | g__Veillonella | s__parvula |
| denovo154 | 10.67663 | -2.62938 | 0.731506 | -3.59447 | 0.000325 | 0.003766 | k__Bacteria | p__Firmicutes | c__Clostridia | o__Clostridiales | f__ | g__ | s__ |
| denovo336 | 22.30167 | -1.13275 | 0.315296 | -3.59264 | 0.000327 | 0.003766 | k__Bacteria | p__Firmicutes | c__Clostridia | o__Clostridiales | f__Ruminococcaceae | g__Oscillospira | s__ |
| denovo367 | 7.721513 | 1.998871 | 0.556097 | 3.594463 | 0.000325 | 0.003766 | k__Bacteria | p__Firmicutes | c__Clostridia | o__Clostridiales | f__Lachnospiraceae | g__ | s__ |
| denovo556 | 2.144542 | -2.79331 | 0.776361 | -3.59796 | 0.000321 | 0.003766 | k__Bacteria | p__Firmicutes | c__Clostridia | o__Clostridiales | f__Ruminococcaceae | g__ | s__ |
| denovo160 | 6.335407 | -4.47506 | 1.25036 | -3.57902 | 0.000345 | 0.003898 | k__Bacteria | p__Firmicutes | c__Clostridia | o__Clostridiales | f__Veillonellaceae | g__Megamonas | s__ |
| denovo128 | 17.44237 | -3.07098 | 0.863675 | -3.55571 | 0.000377 | 0.004194 | k__Bacteria | p__Tenericutes | c__Mollicutes | o__Anaeroplasmatales | f__Anaeroplasmataceae | g__ | s__ |
| denovo55 | 347.4023 | 0.720898 | 0.202853 | 3.553792 | 0.00038 | 0.004194 | k__Bacteria | p__Firmicutes | c__Clostridia | o__Clostridiales | f__Lachnospiraceae | g__[Ruminococcus] | s__ |
| denovo507 | 4.21143 | -3.04802 | 0.859372 | -3.5468 | 0.00039 | 0.004259 | k__Bacteria | p__Firmicutes | c__Clostridia | o__Clostridiales | f__Ruminococcaceae | g__ | s__ |
| denovo448 | 2.869041 | -2.08497 | 0.589583 | -3.53635 | 0.000406 | 0.004381 | k__Bacteria | p__Firmicutes | c__Clostridia | o__Clostridiales | f__Ruminococcaceae | g__ | s__ |
| denovo432 | 3.978587 | -2.8173 | 0.799428 | -3.52414 | 0.000425 | 0.004538 | k__Bacteria | p__Firmicutes | c__Clostridia | o__Clostridiales | f__ | g__ | s__ |
| denovo26 | 911.9465 | -0.80305 | 0.228901 | -3.50828 | 0.000451 | 0.004765 | k__Bacteria | p__Firmicutes | c__Clostridia | o__Clostridiales | f__Lachnospiraceae | g__ | s__ |
| denovo471 | 7.907773 | -2.07966 | 0.594657 | -3.49725 | 0.00047 | 0.004861 | k__Bacteria | p__Firmicutes | c__Clostridia | o__Clostridiales | f__Peptococcaceae | g__rc4-4 | s__ |
| denovo559 | 1.829118 | -2.64761 | 0.75674 | -3.4987 | 0.000468 | 0.004861 | k__Bacteria | p__Firmicutes | c__Clostridia | o__Clostridiales | f__Ruminococcaceae | g__ | s__ |
| denovo260 | 7.009734 | -4.16265 | 1.193335 | -3.48825 | 0.000486 | 0.004908 | k__Bacteria | p__Firmicutes | c__Clostridia | o__Clostridiales | f__ | g__ | s__ |
| denovo362 | 5.811159 | 1.5843 | 0.453964 | 3.48992 | 0.000483 | 0.004908 | k__Bacteria | p__Firmicutes | c__Clostridia | o__Clostridiales | f__Lachnospiraceae | g__Dorea | s__ |
| denovo70 | 157.8566 | -0.82604 | 0.236939 | -3.48629 | 0.00049 | 0.004908 | k__Bacteria | p__Firmicutes | c__Clostridia | o__Clostridiales | f__ | g__ | s__ |
| denovo667 | 18.6843 | -1.32985 | 0.382648 | -3.47539 | 0.00051 | 0.005059 | k__Bacteria | p__Firmicutes | c__Clostridia | o__Clostridiales | f__Lachnospiraceae | NA | NA |
| denovo482 | 5.986595 | -1.31989 | 0.381036 | -3.46396 | 0.000532 | 0.005226 | k__Bacteria | p__Firmicutes | c__Clostridia | o__Clostridiales | f__ | g__ | s__ |
| denovo102 | 108.6046 | -0.84994 | 0.246866 | -3.44292 | 0.000575 | 0.005545 | k__Bacteria | p__Firmicutes | c__Clostridia | o__Clostridiales | f__Ruminococcaceae | g__Oscillospira | s__ |
| denovo159 | 28.40424 | 1.705778 | 0.49549 | 3.442609 | 0.000576 | 0.005545 | k__Bacteria | p__Actinobacteria | c__Coriobacteriia | o__Coriobacteriales | f__Coriobacteriaceae | g__ | s__ |
| denovo406 | 2.456734 | -4.4612 | 1.300488 | -3.4304 | 0.000603 | 0.005743 | k__Bacteria | p__Tenericutes | c__Mollicutes | o__RF39 | f__ | g__ | s__ |
| denovo124 | 55.26544 | -2.26533 | 0.663274 | -3.41537 | 0.000637 | 0.006011 | k__Bacteria | p__Firmicutes | c__Clostridia | o__Clostridiales | f__Ruminococcaceae | g__Ruminococcus | s__ |
| denovo425 | 1.464062 | 2.779324 | 0.819625 | 3.39097 | 0.000696 | 0.006344 | k__Bacteria | p__Firmicutes | c__Bacilli | o__Lactobacillales | f__Lactobacillaceae | g__Lactobacillus | s__ |
| denovo49 | 174.2985 | -1.48476 | 0.437394 | -3.39456 | 0.000687 | 0.006344 | k__Bacteria | p__Firmicutes | c__Clostridia | o__Clostridiales | f__Ruminococcaceae | g__ | s__ |
| denovo495 | 5.199615 | 1.494797 | 0.440485 | 3.393527 | 0.00069 | 0.006344 | k__Bacteria | p__Firmicutes | c__Clostridia | o__Clostridiales | f__Lachnospiraceae | g__ | s__ |
| denovo632 | 1.78462 | 1.807836 | 0.533247 | 3.390242 | 0.000698 | 0.006344 | k__Bacteria | p__Firmicutes | c__Bacilli | o__Lactobacillales | f__Streptococcaceae | g__Streptococcus | s__anginosus |
| denovo15 | 3140.375 | 0.670073 | 0.198308 | 3.378948 | 0.000728 | 0.006479 | k__Bacteria | p__Bacteroidetes | c__Bacteroidia | o__Bacteroidales | f__Bacteroidaceae | g__Bacteroides | s__ |
| denovo186 | 5.525566 | -3.12586 | 0.925672 | -3.37686 | 0.000733 | 0.006479 | k__Bacteria | p__Firmicutes | c__Clostridia | o__Clostridiales | f__Christensenellaceae | g__ | s__ |
| denovo8 | 2789.028 | -1.60588 | 0.474965 | -3.38106 | 0.000722 | 0.006479 | k__Bacteria | p__Bacteroidetes | c__Bacteroidia | o__Bacteroidales | f__Prevotellaceae | g__Prevotella | s__copri |
| denovo552 | 5.724107 | -1.35131 | 0.400729 | -3.37213 | 0.000746 | 0.006532 | k__Bacteria | p__Firmicutes | c__Clostridia | o__Clostridiales | f__Christensenellaceae | g__ | s__ |
| denovo173 | 12.16714 | -2.9666 | 0.88265 | -3.36101 | 0.000777 | 0.006739 | k__Bacteria | p__Tenericutes | c__Mollicutes | o__RF39 | f__ | g__ | s__ |
| denovo983 | 7.673751 | -1.36659 | 0.41444 | -3.29744 | 0.000976 | 0.008393 | k__Bacteria | p__Bacteroidetes | c__Bacteroidia | o__Bacteroidales | f__Bacteroidaceae | g__Bacteroides | s__ |
| denovo1764 | 2.223677 | -1.65821 | 0.506222 | -3.27566 | 0.001054 | 0.008988 | k__Bacteria | p__Firmicutes | c__Clostridia | o__Clostridiales | f__ | g__ | s__ |
| denovo428 | 5.289036 | -1.99422 | 0.610233 | -3.26796 | 0.001083 | 0.009115 | k__Bacteria | p__Firmicutes | c__Clostridia | o__Clostridiales | f__Peptococcaceae | g__ | s__ |
| denovo46 | 138.204 | -1.7237 | 0.527648 | -3.26677 | 0.001088 | 0.009115 | k__Bacteria | p__Bacteroidetes | c__Bacteroidia | o__Bacteroidales | f__Bacteroidaceae | g__Bacteroides | s__ |
| denovo281 | 11.50213 | -2.23257 | 0.686871 | -3.25034 | 0.001153 | 0.009477 | k__Bacteria | p__Bacteroidetes | c__Bacteroidia | o__Bacteroidales | f__Bacteroidaceae | g__Bacteroides | s__ |
| denovo36 | 462.1493 | -1.26252 | 0.388311 | -3.2513 | 0.001149 | 0.009477 | k__Bacteria | p__Firmicutes | c__Clostridia | o__Clostridiales | f__ | g__ | s__ |
| denovo828 | 14.18922 | -0.79365 | 0.244313 | -3.24848 | 0.00116 | 0.009477 | k__Bacteria | p__Firmicutes | c__Clostridia | o__Clostridiales | f__Lachnospiraceae | g__ | s__ |

**Supplementary table 11.** Differential abundance of OTUs by Health deficit (FI) as a factor, adjusted for IMD level. Results are where q < 0.01

|  | baseMean | log2FoldChange | lfcSE | stat | pvalue | padj | Kingdom | Phylum | Class | Order | Family | Genus | Species |
| --- | --- | --- | --- | --- | --- | --- | --- | --- | --- | --- | --- | --- | --- |
| denovo102 | 105.3545 | -0.8507 | 0.242477 | -3.50838 | 0.000451 | 0.005019 | k__Bacteria | p__Firmicutes | c__Clostridia | o__Clostridiales | f__Ruminococcaceae | g__Oscillospira | s__ |
| denovo1031 | 4.677398 | 1.807253 | 0.339528 | 5.322832 | 1.02E-07 | 4.50E-06 | k__Bacteria | p__Firmicutes | c__Clostridia | o__Clostridiales | f__Ruminococcaceae | g__Ruminococcus | s__ |
| denovo108 | 14.9424 | -4.63886 | 1.040157 | -4.45977 | 8.20E-06 | 0.000189 | k__Bacteria | p__Tenericutes | c__Mollicutes | o__RF39 | f__ | g__ | s__ |
| denovo109 | 101.2591 | -2.13456 | 0.494386 | -4.3176 | 1.58E-05 | 0.000325 | k__Bacteria | p__Firmicutes | c__Clostridia | o__Clostridiales | f__ | g__ | s__ |
| denovo11 | 1197.368 | 1.113064 | 0.267479 | 4.161319 | 3.16E-05 | 0.000562 | k__Bacteria | p__Bacteroidetes | c__Bacteroidia | o__Bacteroidales | f__Porphyromonadaceae | g__Parabacteroides | s__ |
| denovo114 | 29.2759 | -5.39971 | 0.789583 | -6.83869 | 7.99E-12 | 1.62E-09 | k__Bacteria | p__Bacteroidetes | c__Bacteroidia | o__Bacteroidales | f__Bacteroidaceae | g__Bacteroides | s__coprophilus |
| denovo119 | 48.99673 | -4.70487 | 0.846474 | -5.5582 | 2.73E-08 | 1.45E-06 | k__Bacteria | p__Tenericutes | c__Mollicutes | o__RF39 | f__ | g__ | s__ |
| denovo123 | 60.4053 | -1.57826 | 0.396385 | -3.98164 | 6.84E-05 | 0.00105 | k__Bacteria | p__Firmicutes | c__Clostridia | o__Clostridiales | f__Ruminococcaceae | g__ | s__ |
| denovo1237 | 2.187061 | 1.78591 | 0.393791 | 4.535176 | 5.76E-06 | 0.000142 | k__Bacteria | p__Actinobacteria | c__Actinobacteria | o__Bifidobacteriales | f__Bifidobacteriaceae | g__Bifidobacterium | s__ |
| denovo124 | 55.0622 | -2.33204 | 0.643914 | -3.62167 | 0.000293 | 0.003648 | k__Bacteria | p__Firmicutes | c__Clostridia | o__Clostridiales | f__Ruminococcaceae | g__Ruminococcus | s__ |
| denovo1292 | 2.440245 | -3.46361 | 0.842294 | -4.11211 | 3.92E-05 | 0.000662 | k__Bacteria | p__Bacteroidetes | c__Bacteroidia | o__Bacteroidales | f__Rikenellaceae | g__ | s__ |
| denovo132 | 33.38158 | -4.45738 | 1.095724 | -4.06798 | 4.74E-05 | 0.000788 | k__Bacteria | p__Tenericutes | c__Mollicutes | o__RF39 | f__ | g__ | s__ |
| denovo133 | 161.9991 | 2.141252 | 0.348702 | 6.140632 | 8.22E-10 | 7.57E-08 | k__Bacteria | p__Firmicutes | c__Clostridia | o__Clostridiales | f__Veillonellaceae | g__Veillonella | s__dispar |
| denovo136 | 61.93685 | -3.00701 | 0.441393 | -6.81256 | 9.59E-12 | 1.62E-09 | k__Bacteria | p__Firmicutes | c__Clostridia | o__Clostridiales | f__ | g__ | s__ |
| denovo138 | 253.5578 | 1.085521 | 0.250232 | 4.338057 | 1.44E-05 | 0.000303 | k__Bacteria | p__Bacteroidetes | c__Bacteroidia | o__Bacteroidales | f__Bacteroidaceae | g__Bacteroides | s__ovatus |
| denovo139 | 37.30809 | -5.97299 | 0.945397 | -6.31797 | 2.65E-10 | 2.98E-08 | k__Bacteria | p__Tenericutes | c__Mollicutes | o__RF39 | f__ | g__ | s__ |
| denovo140 | 27.24113 | -3.65101 | 0.794859 | -4.59328 | 4.36E-06 | 0.000113 | k__Bacteria | p__Proteobacteria | c__Alphaproteobacteria | o__RF32 | f__ | g__ | s__ |
| denovo142 | 76.70004 | 1.04566 | 0.22285 | 4.692214 | 2.70E-06 | 7.87E-05 | k__Bacteria | p__Firmicutes | c__Clostridia | o__Clostridiales | f__Ruminococcaceae | g__Oscillospira | s__ |
| denovo144 | 93.48644 | 1.024195 | 0.218801 | 4.680938 | 2.86E-06 | 7.94E-05 | k__Bacteria | p__Firmicutes | c__Clostridia | o__Clostridiales | f__Lachnospiraceae | g__Blautia | s__ |
| denovo147 | 43.57382 | -3.33766 | 0.809688 | -4.12216 | 3.75E-05 | 0.000645 | k__Bacteria | p__Tenericutes | c__Mollicutes | o__RF39 | f__ | g__ | s__ |
| denovo15 | 3004.782 | 0.780602 | 0.192768 | 4.049434 | 5.13E-05 | 0.000839 | k__Bacteria | p__Bacteroidetes | c__Bacteroidia | o__Bacteroidales | f__Bacteroidaceae | g__Bacteroides | s__ |
| denovo150 | 34.65557 | -4.92876 | 0.822317 | -5.99374 | 2.05E-09 | 1.73E-07 | k__Bacteria | p__Firmicutes | c__Clostridia | o__Clostridiales | f__Ruminococcaceae | g__Ruminococcus | s__ |
| denovo153 | 41.23135 | 2.250821 | 0.383024 | 5.876448 | 4.19E-09 | 3.24E-07 | k__Bacteria | p__Firmicutes | c__Clostridia | o__Clostridiales | f__Lachnospiraceae | g__Dorea | s__ |
| denovo154 | 15.34907 | -3.09182 | 0.720047 | -4.29392 | 1.76E-05 | 0.000336 | k__Bacteria | p__Firmicutes | c__Clostridia | o__Clostridiales | f__ | g__ | s__ |
| denovo158 | 12.17896 | -5.64006 | 0.97842 | -5.76446 | 8.19E-09 | 5.53E-07 | k__Bacteria | p__Firmicutes | c__Clostridia | o__Clostridiales | f__Ruminococcaceae | g__ | s__ |
| denovo160 | 4.37556 | -4.28475 | 1.214377 | -3.52835 | 0.000418 | 0.004738 | k__Bacteria | p__Firmicutes | c__Clostridia | o__Clostridiales | f__Veillonellaceae | g__Megamonas | s__ |
| denovo161 | 34.96093 | 1.993108 | 0.437196 | 4.558842 | 5.14E-06 | 0.00013 | k__Bacteria | p__Firmicutes | c__Erysipelotrichi | o__Erysipelotrichales | f__Erysipelotrichaceae | g__ | s__ |
| denovo164 | 12.19357 | -3.68326 | 0.958796 | -3.84155 | 0.000122 | 0.001745 | k__Bacteria | p__Proteobacteria | c__Betaproteobacteria | o__Burkholderiales | f__Comamonadaceae | g__ | s__ |
| denovo165 | 35.24756 | 1.41187 | 0.262315 | 5.382348 | 7.35E-08 | 3.55E-06 | k__Bacteria | p__Firmicutes | c__Clostridia | o__Clostridiales | f__Lachnospiraceae | g__ | s__ |
| denovo168 | 14.17635 | -2.8414 | 0.677935 | -4.19125 | 2.77E-05 | 0.00052 | k__Bacteria | p__Proteobacteria | c__Alphaproteobacteria | o__RF32 | f__ | g__ | s__ |
| denovo1686 | 2.125497 | -1.99632 | 0.555801 | -3.5918 | 0.000328 | 0.00396 | k__Bacteria | p__Firmicutes | c__Clostridia | o__Clostridiales | f__Ruminococcaceae | g__ | s__ |
| denovo173 | 19.46853 | -4.49171 | 0.88583 | -5.07063 | 3.97E-07 | 1.43E-05 | k__Bacteria | p__Tenericutes | c__Mollicutes | o__RF39 | f__ | g__ | s__ |
| denovo1764 | 2.148453 | -1.65404 | 0.493325 | -3.35284 | 0.0008 | 0.008023 | k__Bacteria | p__Firmicutes | c__Clostridia | o__Clostridiales | f__ | g__ | s__ |
| denovo18 | 1807.735 | 0.952431 | 0.199688 | 4.769607 | 1.85E-06 | 5.84E-05 | k__Bacteria | p__Bacteroidetes | c__Bacteroidia | o__Bacteroidales | f__Bacteroidaceae | g__Bacteroides | s__ |
| denovo180 | 34.84431 | -2.37497 | 0.386173 | -6.15002 | 7.75E-10 | 7.57E-08 | k__Bacteria | p__Firmicutes | c__Clostridia | o__Clostridiales | f__ | g__ | s__ |
| denovo182 | 23.17258 | 1.218745 | 0.269052 | 4.529769 | 5.90E-06 | 0.000142 | k__Bacteria | p__Firmicutes | c__Clostridia | o__Clostridiales | f__Ruminococcaceae | g__Ruminococcus | s__ |
| denovo186 | 6.548171 | -3.29422 | 0.969996 | -3.39612 | 0.000683 | 0.007065 | k__Bacteria | p__Firmicutes | c__Clostridia | o__Clostridiales | f__Christensenellaceae | g__ | s__ |
| denovo191 | 17.54519 | -2.0464 | 0.622799 | -3.28581 | 0.001017 | 0.009713 | k__Bacteria | p__Cyanobacteria | c__4C0d-2 | o__YS2 | f__ | g__ | s__ |
| denovo192 | 7.464181 | -3.92466 | 1.154906 | -3.39825 | 0.000678 | 0.007065 | k__Bacteria | p__Bacteroidetes | c__Bacteroidia | o__Bacteroidales | f__Prevotellaceae | g__Prevotella | s__ |
| denovo201 | 5.870029 | -5.41477 | 1.154281 | -4.69103 | 2.72E-06 | 7.87E-05 | k__Bacteria | p__Firmicutes | c__Clostridia | o__Clostridiales | f__Ruminococcaceae | g__ | s__ |
| denovo204 | 20.06602 | -2.05439 | 0.557101 | -3.68763 | 0.000226 | 0.003017 | k__Bacteria | p__Firmicutes | c__Clostridia | o__Clostridiales | f__Ruminococcaceae | g__ | s__ |
| denovo206 | 9.433238 | -5.98637 | 1.177534 | -5.08382 | 3.70E-07 | 1.39E-05 | k__Bacteria | p__Tenericutes | c__Mollicutes | o__RF39 | f__ | g__ | s__ |
| denovo217 | 18.81473 | 1.913409 | 0.37421 | 5.113199 | 3.17E-07 | 1.23E-05 | k__Bacteria | p__Firmicutes | c__Clostridia | o__Clostridiales | f__Ruminococcaceae | g__Oscillospira | s__ |
| denovo218 | 46.85138 | -1.73676 | 0.296096 | -5.86554 | 4.48E-09 | 3.24E-07 | k__Bacteria | p__Firmicutes | c__Clostridia | o__Clostridiales | f__Lachnospiraceae | g__ | s__ |
| denovo22 | 845.2048 | 1.035168 | 0.258521 | 4.004196 | 6.22E-05 | 0.000985 | k__Bacteria | p__Actinobacteria | c__Actinobacteria | o__Bifidobacteriales | f__Bifidobacteriaceae | g__Bifidobacterium | s__adolescentis |
| denovo228 | 4.775774 | -4.13312 | 1.079714 | -3.82797 | 0.000129 | 0.001818 | k__Bacteria | p__Firmicutes | c__Clostridia | o__Clostridiales | f__Veillonellaceae | g__Dialister | s__ |
| denovo235 | 7.670679 | 3.445473 | 0.380373 | 9.058149 | 1.33E-19 | 1.34E-16 | k__Bacteria | p__Firmicutes | c__Bacilli | o__Lactobacillales | f__Enterococcaceae | g__Enterococcus | s__ |
| denovo237 | 21.62968 | 1.923934 | 0.491268 | 3.916258 | 8.99E-05 | 0.00132 | k__Bacteria | p__Firmicutes | c__Clostridia | o__Clostridiales | f__Lachnospiraceae | g__Blautia | s__producta |
| denovo24 | 603.3428 | -1.75377 | 0.309252 | -5.67101 | 1.42E-08 | 8.99E-07 | k__Bacteria | p__Bacteroidetes | c__Bacteroidia | o__Bacteroidales | f__Rikenellaceae | NA | NA |
| denovo25 | 828.1551 | 0.89745 | 0.171619 | 5.229303 | 1.70E-07 | 6.89E-06 | k__Bacteria | p__Firmicutes | c__Clostridia | o__Clostridiales | f__Lachnospiraceae | g__Blautia | s__ |
| denovo26 | 898.3827 | -0.96778 | 0.222403 | -4.35148 | 1.35E-05 | 0.000291 | k__Bacteria | p__Firmicutes | c__Clostridia | o__Clostridiales | f__Lachnospiraceae | g__ | s__ |
| denovo260 | 6.39978 | -3.89747 | 1.164949 | -3.34561 | 0.000821 | 0.008154 | k__Bacteria | p__Firmicutes | c__Clostridia | o__Clostridiales | f__ | g__ | s__ |
| denovo263 | 6.487486 | -3.77395 | 0.944211 | -3.99693 | 6.42E-05 | 0.001 | k__Bacteria | p__Tenericutes | c__RF3 | o__ML615J-28 | f__ | g__ | s__ |
| denovo268 | 8.342489 | -3.48993 | 0.884879 | -3.94397 | 8.01E-05 | 0.001194 | k__Bacteria | p__Bacteroidetes | c__Bacteroidia | o__Bacteroidales | f__Rikenellaceae | g__ | s__ |
| denovo272 | 24.17765 | 1.058604 | 0.299277 | 3.537208 | 0.000404 | 0.004655 | k__Bacteria | p__Firmicutes | c__Clostridia | o__Clostridiales | f__Lachnospiraceae | g__ | s__ |
| denovo277 | 28.0913 | -1.54831 | 0.421885 | -3.66997 | 0.000243 | 0.003111 | k__Bacteria | p__Firmicutes | c__Clostridia | o__Clostridiales | f__ | g__ | s__ |
| denovo278 | 8.01777 | 1.573052 | 0.376807 | 4.174686 | 2.98E-05 | 0.00054 | k__Bacteria | p__Firmicutes | c__Erysipelotrichi | o__Erysipelotrichales | f__Erysipelotrichaceae | g__[Eubacterium] | s__dolichum |
| denovo281 | 8.014751 | -3.46723 | 0.662125 | -5.23651 | 1.64E-07 | 6.89E-06 | k__Bacteria | p__Bacteroidetes | c__Bacteroidia | o__Bacteroidales | f__Bacteroidaceae | g__Bacteroides | s__ |
| denovo295 | 8.779943 | 1.524046 | 0.448649 | 3.396965 | 0.000681 | 0.007065 | k__Bacteria | p__Actinobacteria | c__Coriobacteriia | o__Coriobacteriales | f__Coriobacteriaceae | g__Eggerthella | s__lenta |
| denovo322 | 3.869707 | -3.98894 | 1.053082 | -3.78787 | 0.000152 | 0.00208 | k__Bacteria | p__Tenericutes | c__Mollicutes | o__RF39 | f__ | g__ | s__ |
| denovo336 | 21.30957 | -1.06192 | 0.303952 | -3.4937 | 0.000476 | 0.005245 | k__Bacteria | p__Firmicutes | c__Clostridia | o__Clostridiales | f__Ruminococcaceae | g__Oscillospira | s__ |
| denovo350 | 9.900072 | -1.75224 | 0.406285 | -4.31284 | 1.61E-05 | 0.000325 | k__Bacteria | p__Firmicutes | c__Erysipelotrichi | o__Erysipelotrichales | f__Erysipelotrichaceae | g__cc_115 | s__ |
| denovo352 | 7.500502 | -3.25117 | 0.754396 | -4.30964 | 1.64E-05 | 0.000325 | k__Bacteria | p__Firmicutes | c__Clostridia | o__Clostridiales | f__Ruminococcaceae | g__ | s__ |
| denovo361 | 24.49448 | 0.987477 | 0.269049 | 3.670248 | 0.000242 | 0.003111 | k__Bacteria | p__Firmicutes | c__Bacilli | o__Lactobacillales | f__Streptococcaceae | g__Streptococcus | s__ |
| denovo362 | 6.612324 | 1.476837 | 0.445699 | 3.313527 | 0.000921 | 0.008974 | k__Bacteria | p__Firmicutes | c__Clostridia | o__Clostridiales | f__Lachnospiraceae | g__Dorea | s__ |
| denovo364 | 7.945443 | 2.686661 | 0.574346 | 4.677773 | 2.90E-06 | 7.94E-05 | k__Bacteria | p__Firmicutes | c__Clostridia | o__Clostridiales | f__Lachnospiraceae | g__[Ruminococcus] | s__ |
| denovo367 | 7.178556 | 2.947593 | 0.528185 | 5.580605 | 2.40E-08 | 1.35E-06 | k__Bacteria | p__Firmicutes | c__Clostridia | o__Clostridiales | f__Lachnospiraceae | g__ | s__ |
| denovo389 | 3.417266 | -3.06126 | 0.770246 | -3.97439 | 7.06E-05 | 0.001067 | k__Bacteria | p__Firmicutes | c__Clostridia | o__Clostridiales | f__ | g__ | s__ |
| denovo39 | 309.805 | -1.27401 | 0.374251 | -3.40417 | 0.000664 | 0.007065 | k__Bacteria | p__Proteobacteria | c__Gammaproteobacteria | o__Pasteurellales | f__Pasteurellaceae | g__Haemophilus | NA |
| denovo4 | 1766.999 | 1.29747 | 0.364397 | 3.560595 | 0.00037 | 0.004383 | k__Bacteria | p__Proteobacteria | c__Gammaproteobacteria | o__Enterobacteriales | f__Enterobacteriaceae | g__ | s__ |
| denovo40 | 264.3314 | -2.22596 | 0.505606 | -4.40255 | 1.07E-05 | 0.000241 | k__Bacteria | p__Bacteroidetes | c__Bacteroidia | o__Bacteroidales | f__Bacteroidaceae | g__Bacteroides | s__ |
| denovo401 | 4.216656 | -4.33373 | 1.128145 | -3.84146 | 0.000122 | 0.001745 | k__Bacteria | p__Tenericutes | c__Mollicutes | o__RF39 | f__ | g__ | s__ |
| denovo406 | 2.402376 | -4.31313 | 1.250004 | -3.4505 | 0.00056 | 0.006095 | k__Bacteria | p__Tenericutes | c__Mollicutes | o__RF39 | f__ | g__ | s__ |
| denovo411 | 7.026804 | -2.71154 | 0.820046 | -3.30657 | 0.000944 | 0.009112 | k__Bacteria | p__Firmicutes | c__Clostridia | o__Clostridiales | f__ | g__ | s__ |
| denovo427 | 2.827552 | -3.46831 | 0.910463 | -3.80939 | 0.000139 | 0.001933 | k__Bacteria | p__Verrucomicrobia | c__Opitutae | o__[Cerasicoccales] | f__[Cerasicoccaceae] | g__ | s__ |
| denovo438 | 7.155236 | -1.25246 | 0.30385 | -4.12197 | 3.76E-05 | 0.000645 | k__Bacteria | p__Firmicutes | c__Clostridia | o__Clostridiales | f__Ruminococcaceae | g__ | s__ |
| denovo449 | 13.10573 | 1.535473 | 0.278463 | 5.514095 | 3.51E-08 | 1.78E-06 | k__Bacteria | p__Bacteroidetes | c__Bacteroidia | o__Bacteroidales | f__Bacteroidaceae | g__Bacteroides | s__ |
| denovo461 | 2.895282 | 2.256417 | 0.563394 | 4.005039 | 6.20E-05 | 0.000985 | k__Bacteria | p__Firmicutes | c__Bacilli | o__Lactobacillales | f__Lactobacillaceae | g__Lactobacillus | s__zeae |
| denovo469 | 49.16887 | -1.05945 | 0.322677 | -3.28332 | 0.001026 | 0.009713 | k__Bacteria | p__Firmicutes | c__Clostridia | o__Clostridiales | f__Ruminococcaceae | g__Faecalibacterium | s__prausnitzii |
| denovo482 | 5.824657 | -1.25051 | 0.370611 | -3.37419 | 0.00074 | 0.007575 | k__Bacteria | p__Firmicutes | c__Clostridia | o__Clostridiales | f__ | g__ | s__ |
| denovo49 | 168.0284 | -1.43117 | 0.428359 | -3.34107 | 0.000835 | 0.008208 | k__Bacteria | p__Firmicutes | c__Clostridia | o__Clostridiales | f__Ruminococcaceae | g__ | s__ |
| denovo492 | 2.854069 | -3.82443 | 0.806808 | -4.7402 | 2.14E-06 | 6.55E-05 | k__Bacteria | p__Firmicutes | c__Clostridia | o__Clostridiales | f__ | g__ | s__ |
| denovo495 | 4.757867 | 1.84328 | 0.428876 | 4.297932 | 1.72E-05 | 0.000336 | k__Bacteria | p__Firmicutes | c__Clostridia | o__Clostridiales | f__Lachnospiraceae | g__ | s__ |
| denovo496 | 3.083171 | 3.509705 | 0.963213 | 3.643746 | 0.000269 | 0.003402 | k__Bacteria | p__Firmicutes | c__Clostridia | o__Clostridiales | f__Clostridiaceae | g__Clostridium | NA |
| denovo507 | 4.186805 | -3.61153 | 0.823827 | -4.38385 | 1.17E-05 | 0.000257 | k__Bacteria | p__Firmicutes | c__Clostridia | o__Clostridiales | f__Ruminococcaceae | g__ | s__ |
| denovo528 | 1.452486 | -3.03214 | 0.823393 | -3.68249 | 0.000231 | 0.003039 | k__Bacteria | p__Lentisphaerae | c__[Lentisphaeria] | o__Victivallales | f__Victivallaceae | g__ | s__ |
| denovo559 | 2.034579 | -2.70128 | 0.731001 | -3.69531 | 0.00022 | 0.002966 | k__Bacteria | p__Firmicutes | c__Clostridia | o__Clostridiales | f__Ruminococcaceae | g__ | s__ |
| denovo581 | 9.532443 | 1.487563 | 0.330962 | 4.494668 | 6.97E-06 | 0.000164 | k__Bacteria | p__Bacteroidetes | c__Bacteroidia | o__Bacteroidales | f__Bacteroidaceae | g__Bacteroides | s__fragilis |
| denovo582 | 1.607852 | 2.717808 | 0.569456 | 4.772642 | 1.82E-06 | 5.84E-05 | k__Bacteria | p__Firmicutes | c__Bacilli | o__Lactobacillales | f__Streptococcaceae | g__Streptococcus | s__ |
| denovo591 | 1.903668 | -2.64042 | 0.735001 | -3.59241 | 0.000328 | 0.00396 | k__Bacteria | p__Firmicutes | c__Clostridia | o__Clostridiales | f__ | g__ | s__ |
| denovo6 | 1855.755 | 0.902564 | 0.253943 | 3.554198 | 0.000379 | 0.004415 | k__Bacteria | p__Firmicutes | c__Clostridia | o__Clostridiales | f__Ruminococcaceae | g__Ruminococcus | s__ |
| denovo60 | 259.7595 | 1.362225 | 0.269592 | 5.052907 | 4.35E-07 | 1.52E-05 | k__Bacteria | p__Firmicutes | c__Bacilli | o__Lactobacillales | f__Streptococcaceae | g__Streptococcus | s__ |
| denovo604 | 5.035271 | -1.66293 | 0.357887 | -4.64652 | 3.38E-06 | 9.00E-05 | k__Bacteria | p__Firmicutes | c__Clostridia | o__Clostridiales | f__ | g__ | s__ |
| denovo6053 | 1.626384 | 0.950533 | 0.267071 | 3.559102 | 0.000372 | 0.004383 | k__Bacteria | p__Bacteroidetes | c__Bacteroidia | o__Bacteroidales | f__Bacteroidaceae | g__Bacteroides | s__ |
| denovo72 | 274.8887 | -1.20068 | 0.351864 | -3.41234 | 0.000644 | 0.006941 | k__Bacteria | p__Firmicutes | c__Clostridia | o__Clostridiales | f__ | g__ | s__ |
| denovo7275 | 2.976267 | 1.495646 | 0.413229 | 3.619408 | 0.000295 | 0.003648 | k__Bacteria | p__Bacteroidetes | c__Bacteroidia | o__Bacteroidales | f__Bacteroidaceae | g__Bacteroides | s__fragilis |
| denovo73 | 196.3259 | -0.85559 | 0.25486 | -3.35709 | 0.000788 | 0.007979 | k__Bacteria | p__Firmicutes | c__Clostridia | o__Clostridiales | f__Lachnospiraceae | g__Lachnospira | s__ |
| denovo753 | 14.88162 | 2.506954 | 0.466989 | 5.368336 | 7.95E-08 | 3.66E-06 | k__Bacteria | p__Firmicutes | c__Clostridia | o__Clostridiales | f__Lachnospiraceae | g__ | s__ |
| denovo76 | 121.1605 | -4.02443 | 0.594154 | -6.77338 | 1.26E-11 | 1.82E-09 | k__Bacteria | p__Firmicutes | c__Clostridia | o__Clostridiales | f__Ruminococcaceae | g__ | s__ |
| denovo77 | 26.8541 | -6.25913 | 0.786492 | -7.95829 | 1.74E-15 | 8.84E-13 | k__Bacteria | p__Bacteroidetes | c__Bacteroidia | o__Bacteroidales | f__Prevotellaceae | g__Prevotella | s__ |
| denovo78 | 85.03919 | -3.41365 | 0.606535 | -5.62811 | 1.82E-08 | 1.09E-06 | k__Bacteria | p__Firmicutes | c__Clostridia | o__Clostridiales | f__Lachnospiraceae | g__ | s__ |
| denovo8 | 2606.796 | -1.6312 | 0.462545 | -3.52658 | 0.000421 | 0.004738 | k__Bacteria | p__Bacteroidetes | c__Bacteroidia | o__Bacteroidales | f__Prevotellaceae | g__Prevotella | s__copri |
| denovo80 | 35.90876 | -4.55431 | 0.698121 | -6.52366 | 6.86E-11 | 8.69E-09 | k__Bacteria | p__Firmicutes | c__Clostridia | o__Clostridiales | f__ | g__ | s__ |
| denovo88 | 107.1464 | 1.505073 | 0.217013 | 6.935395 | 4.05E-12 | 1.03E-09 | k__Bacteria | p__Firmicutes | c__Clostridia | o__Clostridiales | f__Ruminococcaceae | g__ | s__ |
| denovo93 | 82.34515 | -5.6857 | 0.802947 | -7.08104 | 1.43E-12 | 4.83E-10 | k__Bacteria | p__Tenericutes | c__Mollicutes | o__RF39 | f__ | g__ | s__ |
| denovo95 | 107.8416 | -2.15203 | 0.428745 | -5.01937 | 5.18E-07 | 1.75E-05 | k__Bacteria | p__Firmicutes | c__Clostridia | o__Clostridiales | f__Ruminococcaceae | g__Ruminococcus | s__ |
| denovo987 | 0.858479 | 4.18016 | 0.999858 | 4.180752 | 2.91E-05 | 0.000535 | k__Bacteria | p__Bacteroidetes | c__Bacteroidia | o__Bacteroidales | f__Bacteroidaceae | g__Bacteroides | s__ |

**Supplementary table 12.** Differential abundance of OTUs by Health deficit (FI) as a factor, adjusted for income level. Results are where q < 0.01

| **id** | **baseMean** | **log2FoldChange** | **lfcSE** | **stat** | **pvalue** | **padj** | **Kingdom** | **Phylum** | **Class** | **Order** | **Family** | **Genus** | **Species** |
| --- | --- | --- | --- | --- | --- | --- | --- | --- | --- | --- | --- | --- | --- |
| denovo153 | 29.64742 | 3.535193 | 0.479018 | 7.380083 | 1.58E-13 | 1.45E-10 | k__Bacteria | p__Firmicutes | c__Clostridia | o__Clostridiales | f__Lachnospiraceae | g__Dorea | s__ |
| denovo85 | 64.36995 | -6.20665 | 1.104294 | -5.62047 | 1.90E-08 | 8.73E-06 | k__Bacteria | p__Tenericutes | c__Mollicutes | o__RF39 | f__ | g__ | s__ |
| denovo116 | 68.31312 | -3.29075 | 0.598282 | -5.50034 | 3.79E-08 | 9.57E-06 | k__Bacteria | p__Bacteroidetes | c__Bacteroidia | o__Bacteroidales | f__Rikenellaceae | g__ | s__ |
| denovo150 | 27.76447 | -5.98841 | 1.092112 | -5.48334 | 4.17E-08 | 9.57E-06 | k__Bacteria | p__Firmicutes | c__Clostridia | o__Clostridiales | f__Ruminococcaceae | g__Ruminococcus | s__ |
| denovo114 | 18.73066 | -6.13064 | 1.133331 | -5.4094 | 6.32E-08 | 1.16E-05 | k__Bacteria | p__Bacteroidetes | c__Bacteroidia | o__Bacteroidales | f__Bacteroidaceae | g__Bacteroides | s__coprophilus |
| denovo119 | 54.23954 | -6.37406 | 1.19647 | -5.32739 | 9.96E-08 | 1.31E-05 | k__Bacteria | p__Tenericutes | c__Mollicutes | o__RF39 | f__ | g__ | s__ |
| denovo77 | 16.76355 | -5.56689 | 1.041144 | -5.3469 | 8.95E-08 | 1.31E-05 | k__Bacteria | p__Bacteroidetes | c__Bacteroidia | o__Bacteroidales | f__Prevotellaceae | g__Prevotella | s__ |
| denovo180 | 41.24453 | -2.74541 | 0.523427 | -5.24506 | 1.56E-07 | 1.79E-05 | k__Bacteria | p__Firmicutes | c__Clostridia | o__Clostridiales | f__ | g__ | s__ |
| denovo170 | 4.058749 | -8.3875 | 1.648061 | -5.08932 | 3.59E-07 | 3.66E-05 | k__Bacteria | p__Bacteroidetes | c__Bacteroidia | o__Bacteroidales | f__S24-7 | g__ | s__ |
| denovo18 | 1792.235 | 1.324223 | 0.265084 | 4.995491 | 5.87E-07 | 4.83E-05 | k__Bacteria | p__Bacteroidetes | c__Bacteroidia | o__Bacteroidales | f__Bacteroidaceae | g__Bacteroides | s__ |
| denovo218 | 39.92844 | -1.8813 | 0.377111 | -4.98873 | 6.08E-07 | 4.83E-05 | k__Bacteria | p__Firmicutes | c__Clostridia | o__Clostridiales | f__Lachnospiraceae | g__ | s__ |
| denovo76 | 117.5291 | -3.87855 | 0.778657 | -4.98108 | 6.32E-07 | 4.83E-05 | k__Bacteria | p__Firmicutes | c__Clostridia | o__Clostridiales | f__Ruminococcaceae | g__ | s__ |
| denovo449 | 15.16798 | 1.833584 | 0.386867 | 4.739569 | 2.14E-06 | 0.000151 | k__Bacteria | p__Bacteroidetes | c__Bacteroidia | o__Bacteroidales | f__Bacteroidaceae | g__Bacteroides | s__ |
| denovo24 | 590.8788 | -1.91748 | 0.40912 | -4.68685 | 2.77E-06 | 0.000182 | k__Bacteria | p__Bacteroidetes | c__Bacteroidia | o__Bacteroidales | f__Rikenellaceae | NA | NA |
| denovo721 | 2.180491 | -2.88565 | 0.625333 | -4.61458 | 3.94E-06 | 0.000241 | k__Bacteria | p__Firmicutes | c__Clostridia | o__Clostridiales | f__Ruminococcaceae | g__ | s__ |
| denovo182 | 24.83148 | 1.564693 | 0.356563 | 4.388267 | 1.14E-05 | 0.000655 | k__Bacteria | p__Firmicutes | c__Clostridia | o__Clostridiales | f__Ruminococcaceae | g__Ruminococcus | s__ |
| denovo192 | 7.042402 | -5.81954 | 1.334545 | -4.36069 | 1.30E-05 | 0.000661 | k__Bacteria | p__Bacteroidetes | c__Bacteroidia | o__Bacteroidales | f__Prevotellaceae | g__Prevotella | s__ |
| denovo7275 | 2.355044 | 2.422851 | 0.554949 | 4.365897 | 1.27E-05 | 0.000661 | k__Bacteria | p__Bacteroidetes | c__Bacteroidia | o__Bacteroidales | f__Bacteroidaceae | g__Bacteroides | s__fragilis |
| denovo1237 | 2.342218 | 2.250492 | 0.517544 | 4.348406 | 1.37E-05 | 0.000662 | k__Bacteria | p__Actinobacteria | c__Actinobacteria | o__Bifidobacteriales | f__Bifidobacteriaceae | g__Bifidobacterium | s__ |
| denovo1082 | 4.546245 | -3.23147 | 0.755827 | -4.27541 | 1.91E-05 | 0.000847 | k__Bacteria | p__Verrucomicrobia | c__Verrucomicrobiae | o__Verrucomicrobiales | f__Verrucomicrobiaceae | g__Akkermansia | s__muciniphila |
| denovo364 | 7.376329 | 3.102642 | 0.726339 | 4.271616 | 1.94E-05 | 0.000847 | k__Bacteria | p__Firmicutes | c__Clostridia | o__Clostridiales | f__Lachnospiraceae | g__[Ruminococcus] | s__ |
| denovo22 | 863.9209 | 1.44541 | 0.339944 | 4.251905 | 2.12E-05 | 0.000883 | k__Bacteria | p__Actinobacteria | c__Actinobacteria | o__Bifidobacteriales | f__Bifidobacteriaceae | g__Bifidobacterium | s__adolescentis |
| denovo233 | 8.613803 | -3.67283 | 0.911437 | -4.02972 | 5.58E-05 | 0.002205 | k__Bacteria | p__Firmicutes | c__Clostridia | o__Clostridiales | f__Christensenellaceae | g__ | s__ |
| denovo835 | 10.126 | -2.87502 | 0.714826 | -4.02199 | 5.77E-05 | 0.002205 | k__Bacteria | p__Bacteroidetes | c__Bacteroidia | o__Bacteroidales | f__Bacteroidaceae | g__Bacteroides | s__ |
| denovo139 | 40.89548 | -4.79057 | 1.195873 | -4.00592 | 6.18E-05 | 0.002266 | k__Bacteria | p__Tenericutes | c__Mollicutes | o__RF39 | f__ | g__ | s__ |
| denovo138 | 276.0556 | 1.323791 | 0.3363 | 3.936345 | 8.27E-05 | 0.0029 | k__Bacteria | p__Bacteroidetes | c__Bacteroidia | o__Bacteroidales | f__Bacteroidaceae | g__Bacteroides | s__ovatus |
| denovo80 | 47.33677 | -3.67897 | 0.936428 | -3.92873 | 8.54E-05 | 0.0029 | k__Bacteria | p__Firmicutes | c__Clostridia | o__Clostridiales | f__ | g__ | s__ |
| denovo236 | 5.25271 | -5.92898 | 1.513268 | -3.918 | 8.93E-05 | 0.002924 | k__Bacteria | p__Firmicutes | c__Clostridia | o__Clostridiales | f__ | g__ | s__ |
| denovo278 | 7.555797 | 1.946569 | 0.499207 | 3.899324 | 9.65E-05 | 0.00305 | k__Bacteria | p__Firmicutes | c__Erysipelotrichi | o__Erysipelotrichales | f__Erysipelotrichaceae | g__[Eubacterium] | s__dolichum |
| denovo581 | 8.763402 | 1.710544 | 0.440937 | 3.879336 | 0.000105 | 0.003202 | k__Bacteria | p__Bacteroidetes | c__Bacteroidia | o__Bacteroidales | f__Bacteroidaceae | g__Bacteroides | s__fragilis |
| denovo186 | 4.013346 | -4.82236 | 1.254007 | -3.84556 | 0.00012 | 0.003558 | k__Bacteria | p__Firmicutes | c__Clostridia | o__Clostridiales | f__Christensenellaceae | g__ | s__ |
| denovo546 | 17.70126 | -3.36314 | 0.886891 | -3.79206 | 0.000149 | 0.004281 | k__Bacteria | p__Firmicutes | c__Clostridia | o__Clostridiales | f__ | g__ | s__ |
| denovo427 | 3.765572 | -4.40395 | 1.170347 | -3.76294 | 0.000168 | 0.004666 | k__Bacteria | p__Verrucomicrobia | c__Opitutae | o__[Cerasicoccales] | f__[Cerasicoccaceae] | g__ | s__ |
| denovo217 | 21.46236 | 1.846102 | 0.50456 | 3.658838 | 0.000253 | 0.006833 | k__Bacteria | p__Firmicutes | c__Clostridia | o__Clostridiales | f__Ruminococcaceae | g__Oscillospira | s__ |
| denovo482 | 6.518275 | -1.81955 | 0.506792 | -3.59032 | 0.00033 | 0.008653 | k__Bacteria | p__Firmicutes | c__Clostridia | o__Clostridiales | f__ | g__ | s__ |
| denovo140 | 20.3891 | -3.61878 | 1.017429 | -3.55679 | 0.000375 | 0.009563 | k__Bacteria | p__Proteobacteria | c__Alphaproteobacteria | o__RF32 | f__ | g__ | s__ |

**Supplementary table 13.** Differential abundance of OTUs by Body Mass Index (BMI) as a factor. Results are where q < 0.01

| **id** | **baseMean** | **log2FoldChange** | **lfcSE** | **stat** | **pvalue** | **padj** | **Kingdom** | **Phylum** | **Class** | **Order** | **Family** | **Genus** | **Species** |
| --- | --- | --- | --- | --- | --- | --- | --- | --- | --- | --- | --- | --- | --- |
| denovo60 | 259.7595 | 1.821624 | 0.242815 | 7.502118 | 6.28E-14 | 7.14E-11 | k__Bacteria | p__Firmicutes | c__Bacilli | o__Lactobacillales | f__Streptococcaceae | g__Streptococcus | s__ |
| denovo281 | 13.34623 | -4.46448 | 0.609706 | -7.32234 | 2.44E-13 | 1.03E-10 | k__Bacteria | p__Bacteroidetes | c__Bacteroidia | o__Bacteroidales | f__Bacteroidaceae | g__Bacteroides | s__ |
| denovo69 | 70.76143 | 4.801434 | 0.657081 | 7.307218 | 2.73E-13 | 1.03E-10 | k__Bacteria | p__Bacteroidetes | c__Bacteroidia | o__Bacteroidales | f__Prevotellaceae | g__Prevotella | s__ |
| denovo361 | 23.27886 | 1.656636 | 0.242728 | 6.825064 | 8.79E-12 | 2.50E-09 | k__Bacteria | p__Firmicutes | c__Bacilli | o__Lactobacillales | f__Streptococcaceae | g__Streptococcus | s__ |
| denovo55 | 375.2713 | 1.198437 | 0.180602 | 6.635781 | 3.23E-11 | 7.34E-09 | k__Bacteria | p__Firmicutes | c__Clostridia | o__Clostridiales | f__Lachnospiraceae | g__[Ruminococcus] | s__ |
| denovo175 | 19.57981 | 6.731156 | 1.024582 | 6.569661 | 5.04E-11 | 9.56E-09 | k__Bacteria | p__Bacteroidetes | c__Bacteroidia | o__Bacteroidales | f__[Paraprevotellaceae] | g__[Prevotella] | s__ |
| denovo157 | 6.651653 | 6.649602 | 1.02447 | 6.49077 | 8.54E-11 | 1.39E-08 | k__Bacteria | p__Firmicutes | c__Clostridia | o__Clostridiales | f__Veillonellaceae | g__Megasphaera | s__ |
| denovo144 | 92.51834 | 1.282133 | 0.198833 | 6.44829 | 1.13E-10 | 1.61E-08 | k__Bacteria | p__Firmicutes | c__Clostridia | o__Clostridiales | f__Lachnospiraceae | g__Blautia | s__ |
| denovo107 | 116.9394 | 1.432392 | 0.230027 | 6.227069 | 4.75E-10 | 6.00E-08 | k__Bacteria | p__Firmicutes | c__Clostridia | o__Clostridiales | f__Lachnospiraceae | g__ | s__ |
| denovo114 | 34.13407 | -4.55688 | 0.737481 | -6.17898 | 6.45E-10 | 7.34E-08 | k__Bacteria | p__Bacteroidetes | c__Bacteroidia | o__Bacteroidales | f__Bacteroidaceae | g__Bacteroides | s__coprophilus |
| denovo98 | 31.21046 | -3.77735 | 0.616617 | -6.12592 | 9.02E-10 | 9.32E-08 | k__Bacteria | p__Bacteroidetes | c__Bacteroidia | o__Bacteroidales | f__Bacteroidaceae | g__Bacteroides | s__ |
| denovo125 | 62.35447 | 1.31751 | 0.21811 | 6.040575 | 1.54E-09 | 1.46E-07 | k__Bacteria | p__Firmicutes | c__Clostridia | o__Clostridiales | f__ | g__ | s__ |
| denovo92 | 19.70835 | 4.483635 | 0.745314 | 6.015762 | 1.79E-09 | 1.57E-07 | k__Bacteria | p__Bacteroidetes | c__Bacteroidia | o__Bacteroidales | f__ | g__ | s__ |
| denovo188 | 4.940244 | -4.74694 | 0.80448 | -5.90063 | 3.62E-09 | 2.82E-07 | k__Bacteria | p__Firmicutes | c__Clostridia | o__Clostridiales | f__Lachnospiraceae | g__Anaerostipes | s__ |
| denovo97 | 91.79009 | 0.974375 | 0.165256 | 5.896167 | 3.72E-09 | 2.82E-07 | k__Bacteria | p__Firmicutes | c__Clostridia | o__Clostridiales | f__Lachnospiraceae | g__Dorea | s__formicigenerans |
| denovo154 | 19.06663 | -3.83879 | 0.652915 | -5.87946 | 4.12E-09 | 2.92E-07 | k__Bacteria | p__Firmicutes | c__Clostridia | o__Clostridiales | f__ | g__ | s__ |
| denovo27 | 597.5102 | 0.941398 | 0.162312 | 5.799924 | 6.63E-09 | 4.44E-07 | k__Bacteria | p__Firmicutes | c__Clostridia | o__Clostridiales | f__Lachnospiraceae | g__[Ruminococcus] | s__gnavus |
| denovo13 | 1114.512 | 1.007203 | 0.175798 | 5.729315 | 1.01E-08 | 6.37E-07 | k__Bacteria | p__Firmicutes | c__Clostridia | o__Clostridiales | f__Lachnospiraceae | g__Blautia | s__ |
| denovo31 | 659.8157 | 0.989858 | 0.174996 | 5.656457 | 1.55E-08 | 9.25E-07 | k__Bacteria | p__Firmicutes | c__Clostridia | o__Clostridiales | f__Lachnospiraceae | g__Coprococcus | s__ |
| denovo100 | 212.8879 | 0.925456 | 0.171696 | 5.390087 | 7.04E-08 | 4.00E-06 | k__Bacteria | p__Firmicutes | c__Clostridia | o__Clostridiales | f__Lachnospiraceae | g__ | s__ |
| denovo99 | 36.89106 | 3.382759 | 0.640846 | 5.278586 | 1.30E-07 | 7.05E-06 | k__Bacteria | p__Firmicutes | c__Clostridia | o__Clostridiales | f__Veillonellaceae | g__Acidaminococcus | s__ |
| denovo461 | 2.944255 | 2.651028 | 0.529454 | 5.007095 | 5.53E-07 | 2.86E-05 | k__Bacteria | p__Firmicutes | c__Bacilli | o__Lactobacillales | f__Lactobacillaceae | g__Lactobacillus | s__zeae |
| denovo1026 | 0.924631 | 1.659591 | 0.33398 | 4.969131 | 6.73E-07 | 3.32E-05 | k__Bacteria | p__Firmicutes | c__Clostridia | o__Clostridiales | f__Lachnospiraceae | g__[Ruminococcus] | s__gnavus |
| denovo5 | 3110.987 | 0.848113 | 0.171937 | 4.932706 | 8.11E-07 | 3.84E-05 | k__Bacteria | p__Firmicutes | c__Clostridia | o__Clostridiales | f__ | g__ | s__ |
| denovo58 | 194.0177 | 0.964505 | 0.199752 | 4.828521 | 1.38E-06 | 6.26E-05 | k__Bacteria | p__Firmicutes | c__Clostridia | o__Clostridiales | f__Lachnospiraceae | g__Dorea | s__ |
| denovo761 | 2.915399 | -2.63405 | 0.546767 | -4.8175 | 1.45E-06 | 6.36E-05 | k__Bacteria | p__Firmicutes | c__Clostridia | o__Clostridiales | f__ | g__ | s__ |
| denovo425 | 1.694695 | 3.550059 | 0.738607 | 4.806423 | 1.54E-06 | 6.47E-05 | k__Bacteria | p__Firmicutes | c__Bacilli | o__Lactobacillales | f__Lactobacillaceae | g__Lactobacillus | s__ |
| denovo325 | 2.197993 | 4.634224 | 0.976672 | 4.744913 | 2.09E-06 | 8.18E-05 | k__Bacteria | p__Fusobacteria | c__Fusobacteriia | o__Fusobacteriales | f__Fusobacteriaceae | g__Fusobacterium | s__ |
| denovo35 | 441.8459 | 0.741575 | 0.156072 | 4.751484 | 2.02E-06 | 8.18E-05 | k__Bacteria | p__Firmicutes | c__Clostridia | o__Clostridiales | f__Lachnospiraceae | g__Blautia | s__ |
| denovo87 | 152.7627 | 1.033258 | 0.221495 | 4.664933 | 3.09E-06 | 0.000117 | k__Bacteria | p__Firmicutes | c__Clostridia | o__Clostridiales | f__Lachnospiraceae | g__ | s__ |
| denovo526 | 8.445699 | -1.29775 | 0.281702 | -4.60682 | 4.09E-06 | 0.00015 | k__Bacteria | p__Firmicutes | c__Clostridia | o__Clostridiales | f__Ruminococcaceae | g__Oscillospira | s__ |
| denovo4 | 1515.134 | 1.498784 | 0.326493 | 4.590555 | 4.42E-06 | 0.000157 | k__Bacteria | p__Proteobacteria | c__Gammaproteobacteria | o__Enterobacteriales | f__Enterobacteriaceae | g__ | s__ |
| denovo165 | 34.15303 | 1.095826 | 0.242029 | 4.527668 | 5.96E-06 | 0.000205 | k__Bacteria | p__Firmicutes | c__Clostridia | o__Clostridiales | f__Lachnospiraceae | g__ | s__ |
| denovo484 | 2.070841 | 3.016247 | 0.674183 | 4.473932 | 7.68E-06 | 0.000257 | k__Bacteria | p__Firmicutes | c__Clostridia | o__Clostridiales | f__Lachnospiraceae | g__Blautia | s__producta |
| denovo235 | 7.167199 | 1.604948 | 0.359902 | 4.459397 | 8.22E-06 | 0.000267 | k__Bacteria | p__Firmicutes | c__Bacilli | o__Lactobacillales | f__Enterococcaceae | g__Enterococcus | s__ |
| denovo213 | 34.76941 | 1.188475 | 0.267105 | 4.449466 | 8.61E-06 | 0.000272 | k__Bacteria | p__Firmicutes | c__Clostridia | o__Clostridiales | f__Ruminococcaceae | g__Ruminococcus | s__ |
| denovo582 | 1.221218 | 2.364772 | 0.532772 | 4.438621 | 9.05E-06 | 0.000278 | k__Bacteria | p__Firmicutes | c__Bacilli | o__Lactobacillales | f__Streptococcaceae | g__Streptococcus | s__ |
| denovo1263 | 0.759038 | 1.496632 | 0.337638 | 4.432651 | 9.31E-06 | 0.000279 | k__Bacteria | p__Firmicutes | c__Clostridia | o__Clostridiales | f__Lachnospiraceae | NA | NA |
| denovo336 | 21.30957 | -1.21519 | 0.274646 | -4.42457 | 9.66E-06 | 0.000282 | k__Bacteria | p__Firmicutes | c__Clostridia | o__Clostridiales | f__Ruminococcaceae | g__Oscillospira | s__ |
| denovo268 | 8.705168 | 3.531229 | 0.821741 | 4.297253 | 1.73E-05 | 0.000492 | k__Bacteria | p__Bacteroidetes | c__Bacteroidia | o__Bacteroidales | f__Rikenellaceae | g__ | s__ |
| denovo272 | 24.17765 | 1.161944 | 0.271918 | 4.273136 | 1.93E-05 | 0.000535 | k__Bacteria | p__Firmicutes | c__Clostridia | o__Clostridiales | f__Lachnospiraceae | g__ | s__ |
| denovo1155 | 3.332771 | 1.064906 | 0.249927 | 4.260867 | 2.04E-05 | 0.000538 | k__Bacteria | p__Firmicutes | c__Clostridia | o__Clostridiales | f__Lachnospiraceae | g__Blautia | s__ |
| denovo329 | 12.4673 | 1.275207 | 0.299228 | 4.261657 | 2.03E-05 | 0.000538 | k__Bacteria | p__Firmicutes | c__Clostridia | o__Clostridiales | f__Lachnospiraceae | g__Dorea | s__ |
| denovo20 | 634.9065 | 0.74652 | 0.176116 | 4.238788 | 2.25E-05 | 0.000574 | k__Bacteria | p__Firmicutes | c__Clostridia | o__Clostridiales | f__Lachnospiraceae | g__ | s__ |
| denovo25 | 828.1551 | 0.661414 | 0.156315 | 4.231299 | 2.32E-05 | 0.000574 | k__Bacteria | p__Firmicutes | c__Clostridia | o__Clostridiales | f__Lachnospiraceae | g__Blautia | s__ |
| denovo632 | 1.681633 | 2.100044 | 0.496034 | 4.233667 | 2.30E-05 | 0.000574 | k__Bacteria | p__Firmicutes | c__Bacilli | o__Lactobacillales | f__Streptococcaceae | g__Streptococcus | s__anginosus |
| denovo128 | 18.07581 | -3.20942 | 0.768471 | -4.17638 | 2.96E-05 | 0.000717 | k__Bacteria | p__Tenericutes | c__Mollicutes | o__Anaeroplasmatales | f__Anaeroplasmataceae | g__ | s__ |
| denovo78 | 64.06929 | -2.23794 | 0.539639 | -4.14711 | 3.37E-05 | 0.000798 | k__Bacteria | p__Firmicutes | c__Clostridia | o__Clostridiales | f__Lachnospiraceae | g__ | s__ |
| denovo52 | 199.815 | -1.25514 | 0.305917 | -4.10289 | 4.08E-05 | 0.000947 | k__Bacteria | p__Firmicutes | c__Clostridia | o__Clostridiales | f__Clostridiaceae | g__Clostridium | s__ |
| denovo1061 | 2.80303 | 3.620503 | 0.889253 | 4.071399 | 4.67E-05 | 0.001042 | k__Bacteria | p__Firmicutes | c__Clostridia | o__Clostridiales | f__Lachnospiraceae | g__Dorea | s__ |
| denovo753 | 11.58808 | 1.701669 | 0.417795 | 4.072977 | 4.64E-05 | 0.001042 | k__Bacteria | p__Firmicutes | c__Clostridia | o__Clostridiales | f__Lachnospiraceae | g__ | s__ |
| denovo543 | 1.645955 | 4.301465 | 1.060443 | 4.056289 | 4.99E-05 | 0.00109 | k__Bacteria | p__Firmicutes | c__Erysipelotrichi | o__Erysipelotrichales | f__Erysipelotrichaceae | g__[Eubacterium] | s__dolichum |
| denovo197 | 29.35305 | -1.02478 | 0.253894 | -4.03627 | 5.43E-05 | 0.001165 | k__Bacteria | p__Firmicutes | c__Clostridia | o__Clostridiales | f__Clostridiaceae | g__ | s__ |
| denovo662 | 10.3429 | -0.97253 | 0.243002 | -4.00213 | 6.28E-05 | 0.001322 | k__Bacteria | p__Bacteroidetes | c__Bacteroidia | o__Bacteroidales | f__Rikenellaceae | g__ | s__ |
| denovo22 | 845.2048 | 0.937356 | 0.234755 | 3.992914 | 6.53E-05 | 0.001349 | k__Bacteria | p__Actinobacteria | c__Actinobacteria | o__Bifidobacteriales | f__Bifidobacteriaceae | g__Bifidobacterium | s__adolescentis |
| denovo663 | 1.101371 | 2.918923 | 0.733909 | 3.977229 | 6.97E-05 | 0.001416 | k__Bacteria | p__Firmicutes | c__Bacilli | o__Lactobacillales | f__Lactobacillaceae | g__Lactobacillus | s__ |
| denovo505 | 2.987375 | 1.408293 | 0.35948 | 3.917584 | 8.94E-05 | 0.001784 | k__Bacteria | p__Actinobacteria | c__Coriobacteriia | o__Coriobacteriales | f__Coriobacteriaceae | g__ | s__ |
| denovo1126 | 1.23431 | 1.467979 | 0.37784 | 3.885193 | 0.000102 | 0.002004 | k__Bacteria | p__Firmicutes | c__Clostridia | o__Clostridiales | NA | NA | NA |
| denovo381 | 10.11231 | -1.39571 | 0.362857 | -3.84643 | 0.00012 | 0.00231 | k__Bacteria | p__Firmicutes | c__Clostridia | o__Clostridiales | f__Ruminococcaceae | g__ | s__ |
| denovo412 | 3.999003 | 1.341819 | 0.350518 | 3.828109 | 0.000129 | 0.002447 | k__Bacteria | p__Firmicutes | c__Clostridia | o__Clostridiales | f__[Mogibacteriaceae] | g__ | s__ |
| denovo248 | 14.14809 | 0.689449 | 0.181662 | 3.795239 | 0.000148 | 0.002749 | k__Bacteria | p__Firmicutes | c__Clostridia | o__Clostridiales | f__Christensenellaceae | g__ | s__ |
| denovo111 | 70.56119 | 0.724739 | 0.192256 | 3.769667 | 0.000163 | 0.002998 | k__Bacteria | p__Firmicutes | c__Clostridia | o__Clostridiales | f__Ruminococcaceae | g__Ruminococcus | s__ |
| denovo1451 | 0.995733 | 1.230605 | 0.328903 | 3.741545 | 0.000183 | 0.003301 | k__Bacteria | p__Firmicutes | c__Clostridia | o__Clostridiales | f__Lachnospiraceae | g__ | s__ |
| denovo1686 | 2.125497 | -1.77119 | 0.476937 | -3.71367 | 0.000204 | 0.003629 | k__Bacteria | p__Firmicutes | c__Clostridia | o__Clostridiales | f__Ruminococcaceae | g__ | s__ |
| denovo231 | 27.28781 | -0.64778 | 0.175198 | -3.69744 | 0.000218 | 0.00381 | k__Bacteria | p__Firmicutes | c__Clostridia | o__Clostridiales | f__Ruminococcaceae | g__Oscillospira | s__ |
| denovo923 | 0.707624 | 1.422132 | 0.387824 | 3.66695 | 0.000245 | 0.004229 | k__Bacteria | p__Actinobacteria | c__Coriobacteriia | o__Coriobacteriales | f__Coriobacteriaceae | g__Atopobium | s__ |
| denovo170 | 7.387564 | 4.981834 | 1.379681 | 3.610859 | 0.000305 | 0.004957 | k__Bacteria | p__Bacteroidetes | c__Bacteroidia | o__Bacteroidales | f__S24-7 | g__ | s__ |
| denovo265 | 3.651192 | -5.08054 | 1.406381 | -3.61249 | 0.000303 | 0.004957 | k__Bacteria | p__Tenericutes | c__Mollicutes | o__RF39 | f__ | g__ | s__ |
| denovo566 | 0.905076 | 2.801754 | 0.774973 | 3.615294 | 0.0003 | 0.004957 | k__Bacteria | p__Firmicutes | c__Bacilli | o__Lactobacillales | f__Lactobacillaceae | g__Lactobacillus | s__reuteri |
| denovo875 | 5.108031 | 0.849044 | 0.234631 | 3.618638 | 0.000296 | 0.004957 | k__Bacteria | p__Firmicutes | c__Clostridia | o__Clostridiales | f__Lachnospiraceae | g__Coprococcus | s__ |
| denovo133 | 107.6995 | 1.07838 | 0.299616 | 3.599202 | 0.000319 | 0.005112 | k__Bacteria | p__Firmicutes | c__Clostridia | o__Clostridiales | f__Veillonellaceae | g__Veillonella | s__dispar |
| denovo397 | 9.475785 | -0.7815 | 0.218082 | -3.58351 | 0.000339 | 0.005353 | k__Bacteria | p__Firmicutes | c__Clostridia | o__Clostridiales | f__Ruminococcaceae | g__ | s__ |
| denovo747 | 0.918348 | -1.12058 | 0.313661 | -3.57259 | 0.000353 | 0.005505 | k__Bacteria | p__Firmicutes | c__Clostridia | o__Clostridiales | f__Ruminococcaceae | g__ | s__ |
| denovo445 | 4.883143 | -1.49419 | 0.423585 | -3.52749 | 0.00042 | 0.006446 | k__Bacteria | p__Firmicutes | c__Clostridia | o__Clostridiales | f__Lachnospiraceae | g__Coprococcus | s__ |
| denovo132 | 17.92221 | -3.47129 | 0.985086 | -3.52385 | 0.000425 | 0.006448 | k__Bacteria | p__Tenericutes | c__Mollicutes | o__RF39 | f__ | g__ | s__ |
| denovo489 | 3.741129 | -1.21465 | 0.346812 | -3.50234 | 0.000461 | 0.006822 | k__Bacteria | p__Firmicutes | c__Clostridia | o__Clostridiales | f__Ruminococcaceae | g__ | s__ |
| denovo61 | 79.26717 | 1.651068 | 0.47148 | 3.501883 | 0.000462 | 0.006822 | k__Bacteria | p__Proteobacteria | c__Gammaproteobacteria | o__Enterobacteriales | f__Enterobacteriaceae | g__ | s__ |
| denovo1163 | 1.722063 | 1.050416 | 0.301162 | 3.487876 | 0.000487 | 0.007007 | k__Bacteria | p__Firmicutes | c__Clostridia | o__Clostridiales | f__Lachnospiraceae | g__ | s__ |
| denovo6 | 1855.755 | 0.805408 | 0.230905 | 3.48805 | 0.000487 | 0.007007 | k__Bacteria | p__Firmicutes | c__Clostridia | o__Clostridiales | f__Ruminococcaceae | g__Ruminococcus | s__ |
| denovo1713 | 1.707457 | 1.560513 | 0.45031 | 3.465419 | 0.000529 | 0.007524 | k__Bacteria | p__Bacteroidetes | c__Bacteroidia | o__Bacteroidales | f__Bacteroidaceae | g__Bacteroides | s__ |
| denovo284 | 15.06316 | -1.16785 | 0.337409 | -3.46124 | 0.000538 | 0.007548 | k__Bacteria | p__Firmicutes | c__Clostridia | o__Clostridiales | f__Ruminococcaceae | g__ | s__ |
| denovo1162 | 0.38126 | 1.483133 | 0.429161 | 3.455892 | 0.000548 | 0.007568 | k__Bacteria | p__Actinobacteria | c__Actinobacteria | o__Actinomycetales | f__Actinomycetaceae | g__Actinomyces | s__ |
| denovo96 | 104.4596 | 0.659148 | 0.19084 | 3.453929 | 0.000552 | 0.007568 | k__Bacteria | p__Firmicutes | c__Clostridia | o__Clostridiales | f__Lachnospiraceae | g__Anaerostipes | s__ |
| denovo23 | 658.2744 | 0.626964 | 0.183354 | 3.419424 | 0.000628 | 0.008494 | k__Bacteria | p__Firmicutes | c__Clostridia | o__Clostridiales | f__Lachnospiraceae | g__ | s__ |
| denovo384 | 4.46819 | -1.32237 | 0.387936 | -3.40874 | 0.000653 | 0.008529 | k__Bacteria | p__Firmicutes | c__Erysipelotrichi | o__Erysipelotrichales | f__Erysipelotrichaceae | g__ | s__ |
| denovo46 | 150.4325 | -1.6317 | 0.478091 | -3.41295 | 0.000643 | 0.008529 | k__Bacteria | p__Bacteroidetes | c__Bacteroidia | o__Bacteroidales | f__Bacteroidaceae | g__Bacteroides | s__ |
| denovo53 | 208.0393 | 0.785924 | 0.230431 | 3.410666 | 0.000648 | 0.008529 | k__Bacteria | p__Firmicutes | c__Clostridia | o__Clostridiales | f__ | g__ | s__ |
| denovo225 | 17.643 | 0.697939 | 0.205443 | 3.397246 | 0.000681 | 0.008795 | k__Bacteria | p__Firmicutes | c__Clostridia | o__Clostridiales | f__Lachnospiraceae | g__Dorea | s__ |
| denovo283 | 11.03495 | -1.1189 | 0.330696 | -3.38347 | 0.000716 | 0.009144 | k__Bacteria | p__Firmicutes | c__Clostridia | o__Clostridiales | f__Ruminococcaceae | g__Oscillospira | s__ |
| denovo89 | 156.2638 | 0.691907 | 0.206127 | 3.35671 | 0.000789 | 0.009965 | k__Bacteria | p__Firmicutes | c__Clostridia | o__Clostridiales | f__Lachnospiraceae | g__ | s__ |

**Supplementary table 14.** Differential abundance of OTUs by Body Mass Index (BMI) as a factor, adjusted for education level. Results are where q < 0.01

| **id** | **baseMean** | **log2FoldChange** | **lfcSE** | **stat** | **pvalue** | **padj** | **Kingdom** | **Phylum** | **Class** | **Order** | **Family** | **Genus** | **Species** |
| --- | --- | --- | --- | --- | --- | --- | --- | --- | --- | --- | --- | --- | --- |
| denovo154 | 13.00905 | -5.07018 | 0.686929 | -7.38094 | 1.57E-13 | 1.64E-10 | k__Bacteria | p__Firmicutes | c__Clostridia | o__Clostridiales | f__ | g__ | s__ |
| denovo92 | 17.32573 | 5.204928 | 0.79057 | 6.58377 | 4.59E-11 | 2.39E-08 | k__Bacteria | p__Bacteroidetes | c__Bacteroidia | o__Bacteroidales | f__ | g__ | s__ |
| denovo235 | 5.980658 | 2.264059 | 0.377502 | 5.997479 | 2.00E-09 | 6.97E-07 | k__Bacteria | p__Firmicutes | c__Bacilli | o__Lactobacillales | f__Enterococcaceae | g__Enterococcus | s__ |
| denovo157 | 10.1808 | 6.350748 | 1.091258 | 5.819656 | 5.90E-09 | 1.54E-06 | k__Bacteria | p__Firmicutes | c__Clostridia | o__Clostridiales | f__Veillonellaceae | g__Megasphaera | s__ |
| denovo60 | 252.1968 | 1.504366 | 0.262942 | 5.721293 | 1.06E-08 | 2.21E-06 | k__Bacteria | p__Firmicutes | c__Bacilli | o__Lactobacillales | f__Streptococcaceae | g__Streptococcus | s__ |
| denovo108 | 31.61188 | -5.64176 | 1.025915 | -5.49924 | 3.81E-08 | 5.68E-06 | k__Bacteria | p__Tenericutes | c__Mollicutes | o__RF39 | f__ | g__ | s__ |
| denovo128 | 51.53992 | -4.7128 | 0.854641 | -5.51436 | 3.50E-08 | 5.68E-06 | k__Bacteria | p__Tenericutes | c__Mollicutes | o__Anaeroplasmatales | f__Anaeroplasmataceae | g__ | s__ |
| denovo125 | 64.0238 | 1.276607 | 0.235087 | 5.430371 | 5.62E-08 | 7.33E-06 | k__Bacteria | p__Firmicutes | c__Clostridia | o__Clostridiales | f__ | g__ | s__ |
| denovo98 | 192.1876 | -3.95509 | 0.744538 | -5.31213 | 1.08E-07 | 1.26E-05 | k__Bacteria | p__Bacteroidetes | c__Bacteroidia | o__Bacteroidales | f__Bacteroidaceae | g__Bacteroides | s__ |
| denovo361 | 22.571 | 1.352032 | 0.259406 | 5.212033 | 1.87E-07 | 1.95E-05 | k__Bacteria | p__Firmicutes | c__Bacilli | o__Lactobacillales | f__Streptococcaceae | g__Streptococcus | s__ |
| denovo46 | 197.5845 | -2.69371 | 0.521897 | -5.16138 | 2.45E-07 | 2.32E-05 | k__Bacteria | p__Bacteroidetes | c__Bacteroidia | o__Bacteroidales | f__Bacteroidaceae | g__Bacteroides | s__ |
| denovo13 | 1109.903 | 0.983339 | 0.192937 | 5.096688 | 3.46E-07 | 2.77E-05 | k__Bacteria | p__Firmicutes | c__Clostridia | o__Clostridiales | f__Lachnospiraceae | g__Blautia | s__ |
| denovo249 | 6.775397 | -5.32348 | 1.042984 | -5.10409 | 3.32E-07 | 2.77E-05 | k__Bacteria | p__Firmicutes | c__Clostridia | o__Clostridiales | f__Christensenellaceae | g__ | s__ |
| denovo461 | 2.97498 | 2.930066 | 0.578218 | 5.067406 | 4.03E-07 | 3.00E-05 | k__Bacteria | p__Firmicutes | c__Bacilli | o__Lactobacillales | f__Lactobacillaceae | g__Lactobacillus | s__zeae |
| denovo761 | 3.14441 | -2.98336 | 0.597288 | -4.99484 | 5.89E-07 | 4.09E-05 | k__Bacteria | p__Firmicutes | c__Clostridia | o__Clostridiales | f__ | g__ | s__ |
| denovo167 | 24.90121 | 4.210416 | 0.882945 | 4.768606 | 1.86E-06 | 0.000121 | k__Bacteria | p__Bacteroidetes | c__Bacteroidia | o__Bacteroidales | f__S24-7 | g__ | s__ |
| denovo31 | 632.3344 | 0.896141 | 0.190347 | 4.707929 | 2.50E-06 | 0.000154 | k__Bacteria | p__Firmicutes | c__Clostridia | o__Clostridiales | f__Lachnospiraceae | g__Coprococcus | s__ |
| denovo107 | 121.2823 | 1.142781 | 0.245457 | 4.655737 | 3.23E-06 | 0.000168 | k__Bacteria | p__Firmicutes | c__Clostridia | o__Clostridiales | f__Lachnospiraceae | g__ | s__ |
| denovo2118 | 1.77589 | -1.70981 | 0.366879 | -4.66041 | 3.16E-06 | 0.000168 | k__Bacteria | p__Firmicutes | c__Clostridia | o__Clostridiales | f__Ruminococcaceae | g__Oscillospira | s__ |
| denovo22 | 823.1074 | 1.183219 | 0.253799 | 4.662041 | 3.13E-06 | 0.000168 | k__Bacteria | p__Actinobacteria | c__Actinobacteria | o__Bifidobacteriales | f__Bifidobacteriaceae | g__Bifidobacterium | s__adolescentis |
| denovo1026 | 0.856887 | 1.698732 | 0.366691 | 4.632597 | 3.61E-06 | 0.000179 | k__Bacteria | p__Firmicutes | c__Clostridia | o__Clostridiales | f__Lachnospiraceae | g__[Ruminococcus] | s__gnavus |
| denovo55 | 347.4023 | 0.893461 | 0.194882 | 4.584623 | 4.55E-06 | 0.000208 | k__Bacteria | p__Firmicutes | c__Clostridia | o__Clostridiales | f__Lachnospiraceae | g__[Ruminococcus] | s__ |
| denovo97 | 88.76361 | 0.824307 | 0.179879 | 4.582551 | 4.59E-06 | 0.000208 | k__Bacteria | p__Firmicutes | c__Clostridia | o__Clostridiales | f__Lachnospiraceae | g__Dorea | s__formicigenerans |
| denovo100 | 220.8351 | 0.826804 | 0.18488 | 4.472124 | 7.74E-06 | 0.000337 | k__Bacteria | p__Firmicutes | c__Clostridia | o__Clostridiales | f__Lachnospiraceae | g__ | s__ |
| denovo528 | 2.376504 | -3.56935 | 0.808735 | -4.41349 | 1.02E-05 | 0.000424 | k__Bacteria | p__Lentisphaerae | c__[Lentisphaeria] | o__Victivallales | f__Victivallaceae | g__ | s__ |
| denovo58 | 189.1894 | 0.943282 | 0.214501 | 4.397568 | 1.09E-05 | 0.000439 | k__Bacteria | p__Firmicutes | c__Clostridia | o__Clostridiales | f__Lachnospiraceae | g__Dorea | s__ |
| denovo99 | 91.28158 | 3.021447 | 0.720251 | 4.194991 | 2.73E-05 | 0.001054 | k__Bacteria | p__Firmicutes | c__Clostridia | o__Clostridiales | f__Veillonellaceae | g__Acidaminococcus | s__ |
| denovo175 | 19.83047 | 4.416988 | 1.062695 | 4.156401 | 3.23E-05 | 0.001163 | k__Bacteria | p__Bacteroidetes | c__Bacteroidia | o__Bacteroidales | f__[Paraprevotellaceae] | g__[Prevotella] | s__ |
| denovo218 | 47.52788 | -1.21263 | 0.291705 | -4.15704 | 3.22E-05 | 0.001163 | k__Bacteria | p__Firmicutes | c__Clostridia | o__Clostridiales | f__Lachnospiraceae | g__ | s__ |
| denovo188 | 3.43574 | -3.39715 | 0.819583 | -4.14498 | 3.40E-05 | 0.001182 | k__Bacteria | p__Firmicutes | c__Clostridia | o__Clostridiales | f__Lachnospiraceae | g__Anaerostipes | s__ |
| denovo389 | 5.710882 | -3.23076 | 0.789067 | -4.0944 | 4.23E-05 | 0.001424 | k__Bacteria | p__Firmicutes | c__Clostridia | o__Clostridiales | f__ | g__ | s__ |
| denovo432 | 4.183699 | -3.11064 | 0.762099 | -4.08167 | 4.47E-05 | 0.001457 | k__Bacteria | p__Firmicutes | c__Clostridia | o__Clostridiales | f__ | g__ | s__ |
| denovo4 | 1601.103 | 1.465296 | 0.361259 | 4.056077 | 4.99E-05 | 0.001513 | k__Bacteria | p__Proteobacteria | c__Gammaproteobacteria | o__Enterobacteriales | f__Enterobacteriaceae | g__ | s__ |
| denovo556 | 2.985484 | -3.02349 | 0.746154 | -4.0521 | 5.08E-05 | 0.001513 | k__Bacteria | p__Firmicutes | c__Clostridia | o__Clostridiales | f__Ruminococcaceae | g__ | s__ |
| denovo604 | 5.432027 | -1.37475 | 0.338673 | -4.05923 | 4.92E-05 | 0.001513 | k__Bacteria | p__Firmicutes | c__Clostridia | o__Clostridiales | f__ | g__ | s__ |
| denovo111 | 70.96254 | 0.843004 | 0.208983 | 4.033832 | 5.49E-05 | 0.001567 | k__Bacteria | p__Firmicutes | c__Clostridia | o__Clostridiales | f__Ruminococcaceae | g__Ruminococcus | s__ |
| denovo489 | 3.572508 | -1.5318 | 0.380172 | -4.02922 | 5.60E-05 | 0.001567 | k__Bacteria | p__Firmicutes | c__Clostridia | o__Clostridiales | f__Ruminococcaceae | g__ | s__ |
| denovo543 | 1.396711 | 4.578969 | 1.13775 | 4.024584 | 5.71E-05 | 0.001567 | k__Bacteria | p__Firmicutes | c__Erysipelotrichi | o__Erysipelotrichales | f__Erysipelotrichaceae | g__[Eubacterium] | s__dolichum |
| denovo610 | 3.096325 | -4.82767 | 1.204158 | -4.00917 | 6.09E-05 | 0.00163 | k__Bacteria | p__Cyanobacteria | c__4C0d-2 | o__YS2 | f__ | g__ | s__ |
| denovo197 | 29.76512 | -1.07293 | 0.274706 | -3.90574 | 9.39E-05 | 0.002367 | k__Bacteria | p__Firmicutes | c__Clostridia | o__Clostridiales | f__Clostridiaceae | g__ | s__ |
| denovo248 | 14.10279 | 0.768589 | 0.196961 | 3.902244 | 9.53E-05 | 0.002367 | k__Bacteria | p__Firmicutes | c__Clostridia | o__Clostridiales | f__Christensenellaceae | g__ | s__ |
| denovo27 | 578.2477 | 0.693175 | 0.177197 | 3.911881 | 9.16E-05 | 0.002367 | k__Bacteria | p__Firmicutes | c__Clostridia | o__Clostridiales | f__Lachnospiraceae | g__[Ruminococcus] | s__gnavus |
| denovo449 | 13.49362 | 1.071801 | 0.275961 | 3.883885 | 0.000103 | 0.002494 | k__Bacteria | p__Bacteroidetes | c__Bacteroidia | o__Bacteroidales | f__Bacteroidaceae | g__Bacteroides | s__ |
| denovo87 | 151.732 | 0.926681 | 0.241297 | 3.840423 | 0.000123 | 0.002911 | k__Bacteria | p__Firmicutes | c__Clostridia | o__Clostridiales | f__Lachnospiraceae | g__ | s__ |
| denovo557 | 2.081644 | -5.41775 | 1.413231 | -3.83359 | 0.000126 | 0.002927 | k__Bacteria | p__Cyanobacteria | c__4C0d-2 | o__YS2 | f__ | g__ | s__ |
| denovo93 | 65.14845 | -2.97023 | 0.777168 | -3.82186 | 0.000132 | 0.003003 | k__Bacteria | p__Tenericutes | c__Mollicutes | o__RF39 | f__ | g__ | s__ |
| denovo1263 | 0.759885 | 1.407261 | 0.369504 | 3.808516 | 0.00014 | 0.003069 | k__Bacteria | p__Firmicutes | c__Clostridia | o__Clostridiales | f__Lachnospiraceae | NA | NA |
| denovo213 | 34.77902 | 1.109478 | 0.292288 | 3.795835 | 0.000147 | 0.003069 | k__Bacteria | p__Firmicutes | c__Clostridia | o__Clostridiales | f__Ruminococcaceae | g__Ruminococcus | s__ |
| denovo445 | 5.052397 | -1.73352 | 0.4561 | -3.80076 | 0.000144 | 0.003069 | k__Bacteria | p__Firmicutes | c__Clostridia | o__Clostridiales | f__Lachnospiraceae | g__Coprococcus | s__ |
| denovo448 | 3.488349 | -2.12238 | 0.558075 | -3.80303 | 0.000143 | 0.003069 | k__Bacteria | p__Firmicutes | c__Clostridia | o__Clostridiales | f__Ruminococcaceae | g__ | s__ |
| denovo492 | 2.943697 | -2.9362 | 0.777102 | -3.7784 | 0.000158 | 0.003175 | k__Bacteria | p__Firmicutes | c__Clostridia | o__Clostridiales | f__ | g__ | s__ |
| denovo52 | 213.421 | -1.26961 | 0.336082 | -3.77767 | 0.000158 | 0.003175 | k__Bacteria | p__Firmicutes | c__Clostridia | o__Clostridiales | f__Clostridiaceae | g__Clostridium | s__ |
| denovo325 | 2.49542 | 3.953734 | 1.05186 | 3.758803 | 0.000171 | 0.003298 | k__Bacteria | p__Fusobacteria | c__Fusobacteriia | o__Fusobacteriales | f__Fusobacteriaceae | g__Fusobacterium | s__ |
| denovo78 | 141.7245 | -2.31246 | 0.615017 | -3.76 | 0.00017 | 0.003298 | k__Bacteria | p__Firmicutes | c__Clostridia | o__Clostridiales | f__Lachnospiraceae | g__ | s__ |
| denovo621 | 3.10866 | -1.87157 | 0.506226 | -3.69711 | 0.000218 | 0.004117 | k__Bacteria | p__Firmicutes | c__Clostridia | o__Clostridiales | f__Clostridiaceae | g__Clostridium | s__ |
| denovo69 | 130.8663 | 2.693593 | 0.729253 | 3.693634 | 0.000221 | 0.004117 | k__Bacteria | p__Bacteroidetes | c__Bacteroidia | o__Bacteroidales | f__Prevotellaceae | g__Prevotella | s__ |
| denovo1061 | 3.078671 | 3.588408 | 0.977077 | 3.672593 | 0.00024 | 0.004393 | k__Bacteria | p__Firmicutes | c__Clostridia | o__Clostridiales | f__Lachnospiraceae | g__Dorea | s__ |
| denovo20 | 634.21 | 0.702423 | 0.192007 | 3.658316 | 0.000254 | 0.004565 | k__Bacteria | p__Firmicutes | c__Clostridia | o__Clostridiales | f__Lachnospiraceae | g__ | s__ |
| denovo662 | 9.925187 | -0.94738 | 0.260677 | -3.63432 | 0.000279 | 0.004927 | k__Bacteria | p__Bacteroidetes | c__Bacteroidia | o__Bacteroidales | f__Rikenellaceae | g__ | s__ |
| denovo336 | 22.30167 | -1.08965 | 0.301152 | -3.61828 | 0.000297 | 0.005155 | k__Bacteria | p__Firmicutes | c__Clostridia | o__Clostridiales | f__Ruminococcaceae | g__Oscillospira | s__ |
| denovo634 | 1.433257 | -3.28542 | 0.909548 | -3.61214 | 0.000304 | 0.005192 | k__Bacteria | p__Firmicutes | c__Clostridia | o__Clostridiales | f__ | g__ | s__ |
| denovo1686 | 2.256564 | -1.87541 | 0.523924 | -3.57954 | 0.000344 | 0.00579 | k__Bacteria | p__Firmicutes | c__Clostridia | o__Clostridiales | f__Ruminococcaceae | g__ | s__ |
| denovo283 | 11.89076 | -1.26169 | 0.354566 | -3.5584 | 0.000373 | 0.006177 | k__Bacteria | p__Firmicutes | c__Clostridia | o__Clostridiales | f__Ruminococcaceae | g__Oscillospira | s__ |
| denovo484 | 1.894108 | 2.496827 | 0.711938 | 3.507086 | 0.000453 | 0.007383 | k__Bacteria | p__Firmicutes | c__Clostridia | o__Clostridiales | f__Lachnospiraceae | g__Blautia | s__producta |
| denovo25 | 805.0458 | 0.59549 | 0.169999 | 3.502896 | 0.00046 | 0.007385 | k__Bacteria | p__Firmicutes | c__Clostridia | o__Clostridiales | f__Lachnospiraceae | g__Blautia | s__ |
| denovo1126 | 1.228079 | 1.406639 | 0.402106 | 3.498177 | 0.000468 | 0.007403 | k__Bacteria | p__Firmicutes | c__Clostridia | o__Clostridiales | f__ | g__ | s__ |
| denovo170 | 3.823315 | 4.95264 | 1.424854 | 3.475893 | 0.000509 | 0.00785 | k__Bacteria | p__Bacteroidetes | c__Bacteroidia | o__Bacteroidales | f__S24-7 | g__ | s__ |
| denovo267 | 10.33428 | -2.17978 | 0.628772 | -3.46673 | 0.000527 | 0.00785 | k__Bacteria | p__Firmicutes | c__Clostridia | o__Clostridiales | f__Ruminococcaceae | g__ | s__ |
| denovo381 | 10.44341 | -1.37112 | 0.39551 | -3.46671 | 0.000527 | 0.00785 | k__Bacteria | p__Firmicutes | c__Clostridia | o__Clostridiales | f__Ruminococcaceae | g__ | s__ |
| denovo526 | 8.582718 | -1.07574 | 0.309885 | -3.47141 | 0.000518 | 0.00785 | k__Bacteria | p__Firmicutes | c__Clostridia | o__Clostridiales | f__Ruminococcaceae | g__Oscillospira | s__ |
| denovo6 | 1898.442 | 0.873858 | 0.252772 | 3.457104 | 0.000546 | 0.008021 | k__Bacteria | p__Firmicutes | c__Clostridia | o__Clostridiales | f__Ruminococcaceae | g__Ruminococcus | s__ |
| denovo61 | 97.338 | 1.809968 | 0.528191 | 3.426729 | 0.000611 | 0.00885 | k__Bacteria | p__Proteobacteria | c__Gammaproteobacteria | o__Enterobacteriales | f__Enterobacteriaceae | g__ | s__ |
| denovo5 | 3061.15 | 0.631044 | 0.185021 | 3.410653 | 0.000648 | 0.009259 | k__Bacteria | p__Firmicutes | c__Clostridia | o__Clostridiales | f__ | g__ | s__ |
| denovo960 | 1.040826 | 1.48098 | 0.435309 | 3.402132 | 0.000669 | 0.009424 | k__Bacteria | p__Firmicutes | c__Clostridia | o__Clostridiales | f__Ruminococcaceae | g__ | s__ |

**Supplementary table 15.** Differential abundance of OTUs by Body Mass Index (BMI) as a factor, adjusted for IMD level. Results are where q < 0.01

| X | baseMean | log2FoldChange | lfcSE | stat | pvalue | padj | Kingdom | Phylum | Class | Order | Family | Genus | Species |
| --- | --- | --- | --- | --- | --- | --- | --- | --- | --- | --- | --- | --- | --- |
| denovo100 | 212.8879 | 0.936397 | 0.17234 | 5.433437 | 5.53E-08 | 3.44E-06 | k__Bacteria | p__Firmicutes | c__Clostridia | o__Clostridiales | f__Lachnospiraceae | g__ | s__ |
| denovo1026 | 0.924631 | 1.715456 | 0.334574 | 5.127288 | 2.94E-07 | 1.59E-05 | k__Bacteria | p__Firmicutes | c__Clostridia | o__Clostridiales | f__Lachnospiraceae | g__[Ruminococcus] | s__gnavus |
| denovo1061 | 2.525756 | 3.310537 | 0.89027 | 3.718575 | 0.0002 | 0.003559 | k__Bacteria | p__Firmicutes | c__Clostridia | o__Clostridiales | f__Lachnospiraceae | g__Dorea | s__ |
| denovo107 | 116.9394 | 1.367205 | 0.230644 | 5.927779 | 3.07E-09 | 3.17E-07 | k__Bacteria | p__Firmicutes | c__Clostridia | o__Clostridiales | f__Lachnospiraceae | g__ | s__ |
| denovo108 | 17.88277 | -4.75247 | 0.916824 | -5.18363 | 2.18E-07 | 1.24E-05 | k__Bacteria | p__Tenericutes | c__Mollicutes | o__RF39 | f__ | g__ | s__ |
| denovo111 | 70.56119 | 0.723905 | 0.193361 | 3.743795 | 0.000181 | 0.003324 | k__Bacteria | p__Firmicutes | c__Clostridia | o__Clostridiales | f__Ruminococcaceae | g__Ruminococcus | s__ |
| denovo1126 | 1.23431 | 1.394654 | 0.379536 | 3.674629 | 0.000238 | 0.004042 | k__Bacteria | p__Firmicutes | c__Clostridia | o__Clostridiales | NA | NA | NA |
| denovo114 | 116.625 | -6.57612 | 0.771661 | -8.52203 | 1.57E-17 | 8.91E-15 | k__Bacteria | p__Bacteroidetes | c__Bacteroidia | o__Bacteroidales | f__Bacteroidaceae | g__Bacteroides | s__coprophilus |
| denovo1155 | 3.332771 | 1.060543 | 0.251404 | 4.218488 | 2.46E-05 | 0.000636 | k__Bacteria | p__Firmicutes | c__Clostridia | o__Clostridiales | f__Lachnospiraceae | g__Blautia | s__ |
| denovo1162 | 0.38126 | 1.44791 | 0.431256 | 3.357426 | 0.000787 | 0.009889 | k__Bacteria | p__Actinobacteria | c__Actinobacteria | o__Actinomycetales | f__Actinomycetaceae | g__Actinomyces | s__ |
| denovo1163 | 1.722063 | 1.035741 | 0.303174 | 3.41633 | 0.000635 | 0.008591 | k__Bacteria | p__Firmicutes | c__Clostridia | o__Clostridiales | f__Lachnospiraceae | g__ | s__ |
| denovo125 | 62.35447 | 1.383864 | 0.218856 | 6.323178 | 2.56E-10 | 2.91E-08 | k__Bacteria | p__Firmicutes | c__Clostridia | o__Clostridiales | f__ | g__ | s__ |
| denovo1263 | 0.759038 | 1.458728 | 0.337477 | 4.322446 | 1.54E-05 | 0.000462 | k__Bacteria | p__Firmicutes | c__Clostridia | o__Clostridiales | f__Lachnospiraceae | NA | NA |
| denovo128 | 23.25545 | -4.56459 | 0.776235 | -5.88042 | 4.09E-09 | 3.88E-07 | k__Bacteria | p__Tenericutes | c__Mollicutes | o__Anaeroplasmatales | f__Anaeroplasmataceae | g__ | s__ |
| denovo13 | 1114.512 | 0.985218 | 0.176742 | 5.574338 | 2.48E-08 | 1.66E-06 | k__Bacteria | p__Firmicutes | c__Clostridia | o__Clostridiales | f__Lachnospiraceae | g__Blautia | s__ |
| denovo133 | 149.8268 | 1.141938 | 0.316327 | 3.609987 | 0.000306 | 0.004879 | k__Bacteria | p__Firmicutes | c__Clostridia | o__Clostridiales | f__Veillonellaceae | g__Veillonella | s__dispar |
| denovo136 | 56.01438 | -1.39873 | 0.397161 | -3.52181 | 0.000429 | 0.006498 | k__Bacteria | p__Firmicutes | c__Clostridia | o__Clostridiales | f__ | g__ | s__ |
| denovo144 | 93.48644 | 0.786918 | 0.199833 | 3.937872 | 8.22E-05 | 0.001699 | k__Bacteria | p__Firmicutes | c__Clostridia | o__Clostridiales | f__Lachnospiraceae | g__Blautia | s__ |
| denovo1451 | 0.995733 | 1.229322 | 0.329473 | 3.73118 | 0.000191 | 0.00344 | k__Bacteria | p__Firmicutes | c__Clostridia | o__Clostridiales | f__Lachnospiraceae | g__ | s__ |
| denovo151 | 38.72738 | -1.97783 | 0.585866 | -3.37591 | 0.000736 | 0.009615 | k__Bacteria | p__Firmicutes | c__Clostridia | o__Clostridiales | f__ | g__ | s__ |
| denovo154 | 48.98751 | -4.02994 | 0.690739 | -5.83425 | 5.40E-09 | 4.62E-07 | k__Bacteria | p__Firmicutes | c__Clostridia | o__Clostridiales | f__ | g__ | s__ |
| denovo157 | 6.384882 | 6.665859 | 0.977974 | 6.815991 | 9.36E-12 | 1.77E-09 | k__Bacteria | p__Firmicutes | c__Clostridia | o__Clostridiales | f__Veillonellaceae | g__Megasphaera | s__ |
| denovo1686 | 2.125497 | -1.66313 | 0.478984 | -3.47221 | 0.000516 | 0.007232 | k__Bacteria | p__Firmicutes | c__Clostridia | o__Clostridiales | f__Ruminococcaceae | g__ | s__ |
| denovo175 | 11.94424 | 6.707939 | 1.006009 | 6.667871 | 2.60E-11 | 3.69E-09 | k__Bacteria | p__Bacteroidetes | c__Bacteroidia | o__Bacteroidales | f__[Paraprevotellaceae] | g__[Prevotella] | s__ |
| denovo188 | 3.485986 | -3.67638 | 0.775154 | -4.74277 | 2.11E-06 | 9.22E-05 | k__Bacteria | p__Firmicutes | c__Clostridia | o__Clostridiales | f__Lachnospiraceae | g__Anaerostipes | s__ |
| denovo192 | 7.750765 | 4.512146 | 1.075748 | 4.194427 | 2.74E-05 | 0.000687 | k__Bacteria | p__Bacteroidetes | c__Bacteroidia | o__Bacteroidales | f__Prevotellaceae | g__Prevotella | s__ |
| denovo197 | 29.35305 | -0.98955 | 0.25546 | -3.8736 | 0.000107 | 0.002139 | k__Bacteria | p__Firmicutes | c__Clostridia | o__Clostridiales | f__Clostridiaceae | g__ | s__ |
| denovo20 | 634.9065 | 0.772504 | 0.176956 | 4.365506 | 1.27E-05 | 0.000404 | k__Bacteria | p__Firmicutes | c__Clostridia | o__Clostridiales | f__Lachnospiraceae | g__ | s__ |
| denovo213 | 34.76941 | 1.186338 | 0.268158 | 4.424032 | 9.69E-06 | 0.000324 | k__Bacteria | p__Firmicutes | c__Clostridia | o__Clostridiales | f__Ruminococcaceae | g__Ruminococcus | s__ |
| denovo218 | 48.50766 | -1.15924 | 0.273009 | -4.24616 | 2.17E-05 | 0.000603 | k__Bacteria | p__Firmicutes | c__Clostridia | o__Clostridiales | f__Lachnospiraceae | g__ | s__ |
| denovo22 | 845.2048 | 0.91225 | 0.236204 | 3.862125 | 0.000112 | 0.002204 | k__Bacteria | p__Actinobacteria | c__Actinobacteria | o__Bifidobacteriales | f__Bifidobacteriaceae | g__Bifidobacterium | s__adolescentis |
| denovo225 | 17.643 | 0.692208 | 0.20646 | 3.352746 | 0.0008 | 0.009889 | k__Bacteria | p__Firmicutes | c__Clostridia | o__Clostridiales | f__Lachnospiraceae | g__Dorea | s__ |
| denovo228 | 4.467821 | -3.20008 | 0.951431 | -3.36344 | 0.00077 | 0.009834 | k__Bacteria | p__Firmicutes | c__Clostridia | o__Clostridiales | f__Veillonellaceae | g__Dialister | s__ |
| denovo231 | 27.28781 | -0.64135 | 0.176182 | -3.64028 | 0.000272 | 0.004488 | k__Bacteria | p__Firmicutes | c__Clostridia | o__Clostridiales | f__Ruminococcaceae | g__Oscillospira | s__ |
| denovo235 | 7.432881 | 2.125194 | 0.364806 | 5.825544 | 5.69E-09 | 4.62E-07 | k__Bacteria | p__Firmicutes | c__Bacilli | o__Lactobacillales | f__Enterococcaceae | g__Enterococcus | s__ |
| denovo24 | 615.119 | -1.04227 | 0.283112 | -3.68149 | 0.000232 | 0.003995 | k__Bacteria | p__Bacteroidetes | c__Bacteroidia | o__Bacteroidales | f__Rikenellaceae | NA | NA |
| denovo248 | 14.14809 | 0.702638 | 0.182336 | 3.853526 | 0.000116 | 0.002244 | k__Bacteria | p__Firmicutes | c__Clostridia | o__Clostridiales | f__Christensenellaceae | g__ | s__ |
| denovo25 | 828.1551 | 0.656911 | 0.157111 | 4.18118 | 2.90E-05 | 0.000687 | k__Bacteria | p__Firmicutes | c__Clostridia | o__Clostridiales | f__Lachnospiraceae | g__Blautia | s__ |
| denovo268 | 7.324316 | 2.912811 | 0.788273 | 3.695183 | 0.00022 | 0.003844 | k__Bacteria | p__Bacteroidetes | c__Bacteroidia | o__Bacteroidales | f__Rikenellaceae | g__ | s__ |
| denovo27 | 597.5102 | 0.939986 | 0.162249 | 5.793471 | 6.89E-09 | 5.23E-07 | k__Bacteria | p__Firmicutes | c__Clostridia | o__Clostridiales | f__Lachnospiraceae | g__[Ruminococcus] | s__gnavus |
| denovo272 | 24.17765 | 1.178918 | 0.273463 | 4.311061 | 1.62E-05 | 0.000474 | k__Bacteria | p__Firmicutes | c__Clostridia | o__Clostridiales | f__Lachnospiraceae | g__ | s__ |
| denovo281 | 9.658096 | -4.98692 | 0.581 | -8.58335 | 9.22E-18 | 8.91E-15 | k__Bacteria | p__Bacteroidetes | c__Bacteroidia | o__Bacteroidales | f__Bacteroidaceae | g__Bacteroides | s__ |
| denovo31 | 659.8157 | 0.954435 | 0.175878 | 5.426676 | 5.74E-08 | 3.44E-06 | k__Bacteria | p__Firmicutes | c__Clostridia | o__Clostridiales | f__Lachnospiraceae | g__Coprococcus | s__ |
| denovo325 | 3.053071 | 4.295353 | 0.985663 | 4.357831 | 1.31E-05 | 0.000404 | k__Bacteria | p__Fusobacteria | c__Fusobacteriia | o__Fusobacteriales | f__Fusobacteriaceae | g__Fusobacterium | s__ |
| denovo336 | 21.30957 | -1.08941 | 0.275699 | -3.95143 | 7.77E-05 | 0.001636 | k__Bacteria | p__Firmicutes | c__Clostridia | o__Clostridiales | f__Ruminococcaceae | g__Oscillospira | s__ |
| denovo337 | 9.742227 | -3.41909 | 0.938418 | -3.64346 | 0.000269 | 0.004488 | k__Bacteria | p__Firmicutes | c__Clostridia | o__Clostridiales | f__Ruminococcaceae | g__ | s__ |
| denovo35 | 441.8459 | 0.706546 | 0.156903 | 4.503063 | 6.70E-06 | 0.000254 | k__Bacteria | p__Firmicutes | c__Clostridia | o__Clostridiales | f__Lachnospiraceae | g__Blautia | s__ |
| denovo361 | 23.27886 | 1.669564 | 0.24417 | 6.837706 | 8.05E-12 | 1.77E-09 | k__Bacteria | p__Firmicutes | c__Bacilli | o__Lactobacillales | f__Streptococcaceae | g__Streptococcus | s__ |
| denovo381 | 10.11231 | -1.44097 | 0.36431 | -3.95535 | 7.64E-05 | 0.001636 | k__Bacteria | p__Firmicutes | c__Clostridia | o__Clostridiales | f__Ruminococcaceae | g__ | s__ |
| denovo384 | 4.46819 | -1.37334 | 0.388983 | -3.5306 | 0.000415 | 0.006371 | k__Bacteria | p__Firmicutes | c__Erysipelotrichi | o__Erysipelotrichales | f__Erysipelotrichaceae | g__ | s__ |
| denovo389 | 6.295801 | -3.10929 | 0.722958 | -4.30078 | 1.70E-05 | 0.000484 | k__Bacteria | p__Firmicutes | c__Clostridia | o__Clostridiales | f__ | g__ | s__ |
| denovo4 | 1766.999 | 1.158277 | 0.333852 | 3.46943 | 0.000522 | 0.007232 | k__Bacteria | p__Proteobacteria | c__Gammaproteobacteria | o__Enterobacteriales | f__Enterobacteriaceae | g__ | s__ |
| denovo406 | 2.685354 | -3.83979 | 1.140633 | -3.36637 | 0.000762 | 0.009834 | k__Bacteria | p__Tenericutes | c__Mollicutes | o__RF39 | f__ | g__ | s__ |
| denovo412 | 3.999003 | 1.271382 | 0.352759 | 3.60411 | 0.000313 | 0.004879 | k__Bacteria | p__Firmicutes | c__Clostridia | o__Clostridiales | f__[Mogibacteriaceae] | g__ | s__ |
| denovo425 | 1.525931 | 3.111379 | 0.736503 | 4.224527 | 2.39E-05 | 0.000633 | k__Bacteria | p__Firmicutes | c__Bacilli | o__Lactobacillales | f__Lactobacillaceae | g__Lactobacillus | s__ |
| denovo445 | 4.883143 | -1.44325 | 0.42593 | -3.38846 | 0.000703 | 0.009381 | k__Bacteria | p__Firmicutes | c__Clostridia | o__Clostridiales | f__Lachnospiraceae | g__Coprococcus | s__ |
| denovo448 | 3.343059 | -1.97433 | 0.509029 | -3.87861 | 0.000105 | 0.002133 | k__Bacteria | p__Firmicutes | c__Clostridia | o__Clostridiales | f__Ruminococcaceae | g__ | s__ |
| denovo449 | 12.58693 | 1.023813 | 0.254631 | 4.020773 | 5.80E-05 | 0.001293 | k__Bacteria | p__Bacteroidetes | c__Bacteroidia | o__Bacteroidales | f__Bacteroidaceae | g__Bacteroides | s__ |
| denovo453 | 2.159215 | -2.89654 | 0.830319 | -3.48847 | 0.000486 | 0.007173 | k__Bacteria | p__Lentisphaerae | c__[Lentisphaeria] | o__Victivallales | f__Victivallaceae | g__ | s__ |
| denovo46 | 223.0009 | -2.21566 | 0.494132 | -4.48394 | 7.33E-06 | 0.000269 | k__Bacteria | p__Bacteroidetes | c__Bacteroidia | o__Bacteroidales | f__Bacteroidaceae | g__Bacteroides | s__ |
| denovo461 | 2.895282 | 2.467748 | 0.523209 | 4.716565 | 2.40E-06 | 9.75E-05 | k__Bacteria | p__Firmicutes | c__Bacilli | o__Lactobacillales | f__Lactobacillaceae | g__Lactobacillus | s__zeae |
| denovo484 | 1.760985 | 2.728631 | 0.655051 | 4.165523 | 3.11E-05 | 0.000721 | k__Bacteria | p__Firmicutes | c__Clostridia | o__Clostridiales | f__Lachnospiraceae | g__Blautia | s__producta |
| denovo489 | 3.741129 | -1.1706 | 0.349062 | -3.35356 | 0.000798 | 0.009889 | k__Bacteria | p__Firmicutes | c__Clostridia | o__Clostridiales | f__Ruminococcaceae | g__ | s__ |
| denovo5 | 3110.987 | 0.828039 | 0.17259 | 4.797715 | 1.60E-06 | 7.30E-05 | k__Bacteria | p__Firmicutes | c__Clostridia | o__Clostridiales | f__ | g__ | s__ |
| denovo52 | 199.815 | -1.24625 | 0.307516 | -4.05263 | 5.06E-05 | 0.001152 | k__Bacteria | p__Firmicutes | c__Clostridia | o__Clostridiales | f__Clostridiaceae | g__Clostridium | s__ |
| denovo526 | 8.445699 | -1.25841 | 0.283248 | -4.44276 | 8.88E-06 | 0.000306 | k__Bacteria | p__Firmicutes | c__Clostridia | o__Clostridiales | f__Ruminococcaceae | g__Oscillospira | s__ |
| denovo528 | 1.89304 | -3.53905 | 0.750381 | -4.71634 | 2.40E-06 | 9.75E-05 | k__Bacteria | p__Lentisphaerae | c__[Lentisphaeria] | o__Victivallales | f__Victivallaceae | g__ | s__ |
| denovo543 | 1.4876 | 3.951512 | 1.032417 | 3.827439 | 0.000129 | 0.002413 | k__Bacteria | p__Firmicutes | c__Erysipelotrichi | o__Erysipelotrichales | f__Erysipelotrichaceae | g__[Eubacterium] | s__dolichum |
| denovo55 | 375.2713 | 1.201525 | 0.18133 | 6.626163 | 3.45E-11 | 4.35E-09 | k__Bacteria | p__Firmicutes | c__Clostridia | o__Clostridiales | f__Lachnospiraceae | g__[Ruminococcus] | s__ |
| denovo556 | 2.278388 | -2.35199 | 0.676926 | -3.47452 | 0.000512 | 0.007232 | k__Bacteria | p__Firmicutes | c__Clostridia | o__Clostridiales | f__Ruminococcaceae | g__ | s__ |
| denovo566 | 0.905076 | 2.704535 | 0.774837 | 3.490454 | 0.000482 | 0.007173 | k__Bacteria | p__Firmicutes | c__Bacilli | o__Lactobacillales | f__Lactobacillaceae | g__Lactobacillus | s__reuteri |
| denovo58 | 194.0177 | 0.997119 | 0.200958 | 4.961833 | 6.98E-07 | 3.61E-05 | k__Bacteria | p__Firmicutes | c__Clostridia | o__Clostridiales | f__Lachnospiraceae | g__Dorea | s__ |
| denovo582 | 1.361827 | 2.66982 | 0.543188 | 4.915092 | 8.87E-07 | 4.39E-05 | k__Bacteria | p__Firmicutes | c__Bacilli | o__Lactobacillales | f__Streptococcaceae | g__Streptococcus | s__ |
| denovo6 | 1855.755 | 0.78564 | 0.232037 | 3.385846 | 0.00071 | 0.009381 | k__Bacteria | p__Firmicutes | c__Clostridia | o__Clostridiales | f__Ruminococcaceae | g__Ruminococcus | s__ |
| denovo60 | 259.7595 | 1.869097 | 0.243829 | 7.665619 | 1.78E-14 | 5.06E-12 | k__Bacteria | p__Firmicutes | c__Bacilli | o__Lactobacillales | f__Streptococcaceae | g__Streptococcus | s__ |
| denovo604 | 5.262125 | -1.33495 | 0.31526 | -4.23445 | 2.29E-05 | 0.00062 | k__Bacteria | p__Firmicutes | c__Clostridia | o__Clostridiales | f__ | g__ | s__ |
| denovo61 | 164.9699 | 1.745457 | 0.502149 | 3.475974 | 0.000509 | 0.007232 | k__Bacteria | p__Proteobacteria | c__Gammaproteobacteria | o__Enterobacteriales | f__Enterobacteriaceae | g__ | s__ |
| denovo621 | 3.983084 | -2.19205 | 0.490131 | -4.47236 | 7.74E-06 | 0.000275 | k__Bacteria | p__Firmicutes | c__Clostridia | o__Clostridiales | f__Clostridiaceae | g__Clostridium | s__ |
| denovo632 | 1.681633 | 1.9827 | 0.496126 | 3.996368 | 6.43E-05 | 0.001406 | k__Bacteria | p__Firmicutes | c__Bacilli | o__Lactobacillales | f__Streptococcaceae | g__Streptococcus | s__anginosus |
| denovo662 | 10.3429 | -1.02324 | 0.244309 | -4.18829 | 2.81E-05 | 0.000687 | k__Bacteria | p__Bacteroidetes | c__Bacteroidia | o__Bacteroidales | f__Rikenellaceae | g__ | s__ |
| denovo663 | 1.152532 | 3.089368 | 0.738212 | 4.184933 | 2.85E-05 | 0.000687 | k__Bacteria | p__Firmicutes | c__Bacilli | o__Lactobacillales | f__Lactobacillaceae | g__Lactobacillus | s__ |
| denovo69 | 61.65626 | 5.178598 | 0.652116 | 7.941228 | 2.00E-15 | 7.59E-13 | k__Bacteria | p__Bacteroidetes | c__Bacteroidia | o__Bacteroidales | f__Prevotellaceae | g__Prevotella | s__ |
| denovo747 | 0.918348 | -1.13753 | 0.315582 | -3.60455 | 0.000313 | 0.004879 | k__Bacteria | p__Firmicutes | c__Clostridia | o__Clostridiales | f__Ruminococcaceae | g__ | s__ |
| denovo761 | 2.915399 | -2.39807 | 0.5497 | -4.3625 | 1.29E-05 | 0.000404 | k__Bacteria | p__Firmicutes | c__Clostridia | o__Clostridiales | f__ | g__ | s__ |
| denovo78 | 106.9658 | -2.14032 | 0.55881 | -3.83013 | 0.000128 | 0.002413 | k__Bacteria | p__Firmicutes | c__Clostridia | o__Clostridiales | f__Lachnospiraceae | g__ | s__ |
| denovo87 | 152.7627 | 1.047253 | 0.222619 | 4.704238 | 2.55E-06 | 9.99E-05 | k__Bacteria | p__Firmicutes | c__Clostridia | o__Clostridiales | f__Lachnospiraceae | g__ | s__ |
| denovo875 | 5.108031 | 0.819539 | 0.235824 | 3.47521 | 0.00051 | 0.007232 | k__Bacteria | p__Firmicutes | c__Clostridia | o__Clostridiales | f__Lachnospiraceae | g__Coprococcus | s__ |
| denovo92 | 14.40418 | 3.428549 | 0.709515 | 4.832246 | 1.35E-06 | 6.40E-05 | k__Bacteria | p__Bacteroidetes | c__Bacteroidia | o__Bacteroidales | f__ | g__ | s__ |
| denovo923 | 0.707624 | 1.416376 | 0.390078 | 3.631011 | 0.000282 | 0.004586 | k__Bacteria | p__Actinobacteria | c__Coriobacteriia | o__Coriobacteriales | f__Coriobacteriaceae | g__Atopobium | s__ |
| denovo97 | 91.79009 | 0.958361 | 0.166189 | 5.766684 | 8.08E-09 | 5.75E-07 | k__Bacteria | p__Firmicutes | c__Clostridia | o__Clostridiales | f__Lachnospiraceae | g__Dorea | s__formicigenerans |
| denovo98 | 22.31652 | -3.99475 | 0.59624 | -6.69989 | 2.09E-11 | 3.39E-09 | k__Bacteria | p__Bacteroidetes | c__Bacteroidia | o__Bacteroidales | f__Bacteroidaceae | g__Bacteroides | s__ |
| denovo99 | 107.349 | 2.341177 | 0.682435 | 3.430621 | 0.000602 | 0.008249 | k__Bacteria | p__Firmicutes | c__Clostridia | o__Clostridiales | f__Veillonellaceae | g__Acidaminococcus | s__ |

**Supplementary table 16.** Differential abundance of OTUs by Body Mass Index (BMI) as a factor, adjusted for income level. Results are where q < 0.01

| **id** | **baseMean** | **log2FoldChange** | **lfcSE** | **stat** | **pvalue** | **padj** | **Kingdom** | **Phylum** | **Class** | **Order** | **Family** | **Genus** | **Species** |
| --- | --- | --- | --- | --- | --- | --- | --- | --- | --- | --- | --- | --- | --- |
| denovo154 | 8.55906 | -5.57806 | 0.930267 | -5.99619 | 2.02E-09 | 1.88E-06 | k__Bacteria | p__Firmicutes | c__Clostridia | o__Clostridiales | f__ | g__ | s__ |
| denovo255 | 8.983343 | -7.76211 | 1.487678 | -5.2176 | 1.81E-07 | 8.44E-05 | k__Bacteria | p__Bacteroidetes | c__Bacteroidia | o__Bacteroidales | f__Prevotellaceae | g__Prevotella | s__stercorea |
| denovo55 | 352.4599 | 1.413748 | 0.279002 | 5.067159 | 4.04E-07 | 0.000125 | k__Bacteria | p__Firmicutes | c__Clostridia | o__Clostridiales | f__Lachnospiraceae | g__[Ruminococcus] | s__ |
| denovo819 | 33.31392 | 1.630114 | 0.347516 | 4.690763 | 2.72E-06 | 0.000634 | k__Bacteria | p__Firmicutes | c__Clostridia | o__Clostridiales | f__Lachnospiraceae | g__[Ruminococcus] | s__ |
| denovo128 | 47.14058 | -5.84382 | 1.259159 | -4.64105 | 3.47E-06 | 0.000645 | k__Bacteria | p__Tenericutes | c__Mollicutes | o__Anaeroplasmatales | f__Anaeroplasmataceae | g__ | s__ |
| denovo761 | 2.651249 | -3.40711 | 0.764593 | -4.45611 | 8.35E-06 | 0.001295 | k__Bacteria | p__Firmicutes | c__Clostridia | o__Clostridiales | f__ | g__ | s__ |
| denovo111 | 77.44245 | 1.308469 | 0.300354 | 4.356428 | 1.32E-05 | 0.001532 | k__Bacteria | p__Firmicutes | c__Clostridia | o__Clostridiales | f__Ruminococcaceae | g__Ruminococcus | s__ |
| denovo125 | 69.78263 | 1.499914 | 0.344705 | 4.351298 | 1.35E-05 | 0.001532 | k__Bacteria | p__Firmicutes | c__Clostridia | o__Clostridiales | f__ | g__ | s__ |
| denovo528 | 2.023885 | -4.83018 | 1.115122 | -4.33152 | 1.48E-05 | 0.001532 | k__Bacteria | p__Lentisphaerae | c__[Lentisphaeria] | o__Victivallales | f__Victivallaceae | g__ | s__ |
| denovo107 | 124.0357 | 1.480725 | 0.347906 | 4.256103 | 2.08E-05 | 0.001913 | k__Bacteria | p__Firmicutes | c__Clostridia | o__Clostridiales | f__Lachnospiraceae | g__ | s__ |
| denovo461 | 2.519643 | 3.286675 | 0.775615 | 4.23751 | 2.26E-05 | 0.001913 | k__Bacteria | p__Firmicutes | c__Bacilli | o__Lactobacillales | f__Lactobacillaceae | g__Lactobacillus | s__zeae |
| denovo361 | 23.00901 | 1.592889 | 0.381909 | 4.170862 | 3.03E-05 | 0.002354 | k__Bacteria | p__Firmicutes | c__Bacilli | o__Lactobacillales | f__Streptococcaceae | g__Streptococcus | s__ |
| denovo610 | 3.306005 | -6.44018 | 1.557131 | -4.13593 | 3.54E-05 | 0.002532 | k__Bacteria | p__Cyanobacteria | c__4C0d-2 | o__YS2 | f__ | g__ | s__ |
| denovo309 | 1.545091 | -6.78042 | 1.69803 | -3.99311 | 6.52E-05 | 0.004048 | k__Bacteria | p__Proteobacteria | c__Betaproteobacteria | o__Burkholderiales | f__Comamonadaceae | g__Comamonas | s__ |
| denovo8 | 2391.59 | 2.618494 | 0.653871 | 4.004602 | 6.21E-05 | 0.004048 | k__Bacteria | p__Bacteroidetes | c__Bacteroidia | o__Bacteroidales | f__Prevotellaceae | g__Prevotella | s__copri |
| denovo235 | 4.603946 | 2.056881 | 0.519791 | 3.957132 | 7.59E-05 | 0.004414 | k__Bacteria | p__Firmicutes | c__Bacilli | o__Lactobacillales | f__Enterococcaceae | g__Enterococcus | s__ |
| denovo197 | 30.00007 | -1.47964 | 0.377402 | -3.9206 | 8.83E-05 | 0.004837 | k__Bacteria | p__Firmicutes | c__Clostridia | o__Clostridiales | f__Clostridiaceae | g__ | s__ |
| denovo15 | 3227.579 | 1.079024 | 0.280923 | 3.840995 | 0.000123 | 0.006004 | k__Bacteria | p__Bacteroidetes | c__Bacteroidia | o__Bacteroidales | f__Bacteroidaceae | g__Bacteroides | s__ |
| denovo31 | 666.37 | 1.059562 | 0.275459 | 3.846528 | 0.00012 | 0.006004 | k__Bacteria | p__Firmicutes | c__Clostridia | o__Clostridiales | f__Lachnospiraceae | g__Coprococcus | s__ |
| denovo1026 | 0.840954 | 1.978864 | 0.523615 | 3.779234 | 0.000157 | 0.007323 | k__Bacteria | p__Firmicutes | c__Clostridia | o__Clostridiales | f__Lachnospiraceae | g__[Ruminococcus] | s__gnavus |
| denovo22 | 863.9209 | 1.358626 | 0.365281 | 3.719404 | 0.0002 | 0.008853 | k__Bacteria | p__Actinobacteria | c__Actinobacteria | o__Bifidobacteriales | f__Bifidobacteriaceae | g__Bifidobacterium | s__adolescentis |
